# A fractionation method to identify qauntitative changes in protein expression mediated by IGF-1 on the proteome of murine C2C12 myoblasts

**DOI:** 10.1186/1477-5956-7-28

**Published:** 2009-08-11

**Authors:** Charles C King, Kathyrn Bouic, Theodore Friedmann

**Affiliations:** 1 Department of Pediatrics, UCSD School of Medicine, La Jolla, CA 92093, USA

## Abstract

Although much is known about signal transduction downstream of insulin-like growth factor-1 (IGF-1), relatively little is known about the global changes in protein expression induced by this hormone. In this study, the acute effects of IGF-1 on the proteome of murine C2C12 cells were examined. Cells were treated with IGF-1 for up to 24 hours, lysed, and fractionated into cytosolic, nuclear, and insoluble portions. Proteins from the cytosolic fraction were further separated using a new batch ion-exchange chromatography method to reduce sample complexity, followed by two-dimensional (2D) electrophoresis, and identification of selected proteins by mass spectrometry. PDQuest software was utilized to identify and catalogue temporal changes in protein expression during IGF-1 stimulation. In response to IGF-1 stimulation, expression of 23 proteins increased at least three-fold and expression of 17 proteins decreased at least three-fold compared with control un-stimulated C2C12 cells. Changes in expression of selected proteins from each group, including Rho-GDI, cofillin, RAD50, enolase, IκB kinase b (IκBKb) and Hsp70 were confirmed by Western blotting. Additionally, the position of 136 'landmark' proteins whose expression levels and physicochemical properties did not change appreciably or consistently during IGF-1 treatment were mapped and identified. This characterization of large-scale changes in protein expression in response to growth factor stimulation of C2C12 cells will further help to establish a comprehensive understanding of the networks and pathways involved in the action of IGF-1.

## Introduction

The growth factor IGF-1 is one of the major components of the mammalian hypothalamic-pituitary growth axis. Isomers of IGF constitute the principal effector molecules that regulate the body-wide action of growth hormone by inducing the proliferation, growth, cell turnover and function of many tissues, including skeletal muscle [1]. IGF-1 represents one of several alternatively spliced products of the *igf-1 *gene and plays important roles in numerous aspects of the proliferation and growth of many tissues [2-4]. The hypertrophic effects of IGF-1 in murine skeletal muscle result from increased protein synthesis in existing myofibers combined with an activation of growth and differentiation of muscle satellite cells. These findings have led to studies examining the molecular effects of IGF-1 treatment, including potential protective and regenerative effects of exogenous IGF-1 in a variety of animal models of degenerative musculoskeletal diseases and defects, including Duchenne's muscular dystrophy and age-related muscle degeneration, cancer models, and lymphocyte activation [5-8]. These studies have demonstrated that treatment of cells with functional IGF-1 protein or transduction of mouse or rat skeletal muscle cells with vectors encoding IGF-1 induces pleiotrophic effects in many cellular pathways that result in muscle hypertrophy, enhanced muscle contractility and protection from age-related muscle wasting.

Global gene expression studies in a variety of target tissues have identified a number of genes that are either up- or down-regulated by IGF-1 [9-13]. Particularly in the case of IGF-1-induced up-regulation, genes identified include those with mitogenic and differentiation functions, such as mitogen-actvated protein kinase (MAPK) and phosphatidylinositol 3-OH kinase (PI3K) signaling pathways [14], a variety of muscle functions, intracellular signaling, cell cycle, transcriptional and translational functions, cellular respiration, and mitochondrial functions [15]. We have recently examined the effects of IGF-1 on global gene expression in cultured murine C2C12 myoblasts and have identified families of genes whose expression is up- or down-regulated by exposure to IGF-1 for up to 4 hours [16]. These aberrantly-expressed genes fall into a variety of functional families, with the most impressive changes occurring in genes related to steroid biogenesis and fatty acid metabolism.

We have previously used a microarray-based approach to identify changes in expression of genes sets in C2C12 cells in response to IGF-1 treatment. To determine if these transcriptional effects are reflected in alterations of the encoded proteins, we have now examined the effect of IGF-1 on the proteome of C2C12 cells using two-dimensional (2D) electrophoresis and mass spectrometry. In contrast to extensive transcriptional profiling studies of IGF-1 effects, there is very little information available on the proteomic consequences of these transcriptional effects of IGF-1 or of possible biochemical and metabolic changes in IGF-1-treated cells or tissues. It is therefore not yet understood how IGF-1-induced transcriptional changes affect the functions of the gene products and the complex systems effects of IGF-1 or other growth factors. In the present study we have characterized the functional effects of IGF-1 on the cellular proteome and describe in this report the acute response of murine C2C12 skeletal muscle myoblasts to IGF-1. We have carried out an extensive analysis of the proteomes of cultured murine myoblast C2C12 cells exposed acutely to high levels of IGF-1, and identify by mass spectrometry 23 proteins with increased expression, 17 proteins with decreased expression and 136 proteins whose expression was unchanged after treatment of C2C12 cells with IGF-1. Furthermore, we used Western blotting methods to confirm altered levels of a set of the most significantly changed proteins during the 24 hour incubation period. Among the interesting protein changes were increased expression of RAD50, enolase, IκBKb, and Hsp70 and decreased levels of cofilin and Rho-GDI.

## Materials and methods

### Reagents

IGF-1 was purchased from Invitrogen (Carlsbad, CA). All other materials were reagent grade. Antibodies to Rad50, enolase, IκBKb and RhoGDI were from Santa Cruz Biotechnology (Santa Cruz, CA) and the Hsp70 antibody was from BD Biosciences (San Jose, CA). The cofilin antibody was a generous gift from Dr. Gary Bokoch.

### Cell Culture

C2C12 cells were maintained in Dulbecco's modified Eagle's medium containing high glucose (DMEM; Invitrogen) with 10% heat-inactivated fetal bovine serum (Omega Scientific Inc., Tarzana, CA). For these studies, cells (seeded at 1.2 × 10^6 ^cells/10-cm dish) were grown to 70–80% confluence, transferred to serum-free media for 18 h, and treated for 1, 2, 4, or 8 hours with IGF-1 at a final concentration of 100 μM IGF-1.

### Cell Fractionation

Control or IGF-1-stimulated cells were washed three times in PBS, and re-suspended in 0.25 ml Lysis Buffer A (20 mM HEPES, pH 7.9, 10 mM KCl, 0.1 mM EDTA, 1.5 mM μM phenylmethylsulfonyl fluoride (PMSF), 1 mM vanadate, 40 μg/ml leupeptin, and 1 μM MgCl_2_, 300 microcystin) at 4°C. After 30 min, Triton X-100 was added to a final concentration of 0.5% (v/v). Samples were centrifuged at 13,000 × *g *for 5 min at 4°C, and the resulting supernatant-containing soluble cytoplasmic and membrane proteins was removed. The pellet was re-suspended in 0.1 ml Lysis Buffer B (20 mM HEPES, pH 7.9, 420 mM NaCl, 0.1 mM EDTA, 1.5 mM MgCl_2_, and 25% glycerol containing 300 μM PMSF, 1 mM vanadate, 40 μg/ml leupeptin, 1 μM microcystin, and 150 U DNase 1) at 4°C and sonicated (Branson bath sonicator, Danbury, CT). Again, the lysates were centrifuged at 13,000 × *g *for 5 min at 4°C, and the resulting supernatant-containing proteins from the nucleus and other membrane-bound organelles was stored at -80°C for future studies. The detergent-insoluble pellet was re-suspended in 0.1 ml Lysis Buffer C (5 M urea, 2 M thiourea, 2% CHAPS, 2% SB-3, 40 mM Tris, pH 7.5, 10% glycerol, 150 IU/ml aprotinin, 2 μg/ml leupeptin, and 1 mM PMSF).

### Batch Ion-Exchange, de-salting, and concentration

Cytoplasmic proteins were further batch separated into four fractions by adsorption onto slurries of Q Sepharose High Performance beads (GE Healthcare, Chalfont St. Giles, United Kingdom). A 1:1 slurry of beads was washed twice in an excess of Binding Buffer (50 mM Tris, pH 7.5, 1 mM EDTA, 1 mM DTT, and 5 mM MgCl_2_) and centrifuged at 1,700 × g for 2 min. Approximately, 0.5 ml of C2C12 cytoplasmic proteins (3–4 μg/μl) was added to the washed beads and mixed at 4°C for 5 min. The beads were pelleted by gentle centrifugation at 1,700 × g for 2 min, and the resulting supernatant (Flow Through, FT) was removed. The beads were re-suspended in Binding Buffer containing 200 mM NaCl, mixed at 4°C for 5 min, and the beads were pelleted by centrifugation at 1,700 × g for 2 min. The resulting supernatant (200 mM fraction) was removed. The beads were re-suspended in Binding Buffer containing 400 mM NaCl, mixed at 4°C for 5 min, and the beads were pelleted by centrifugation at 1,700 × g for 2 min. The resulting supernatant (400 mM fraction) was removed. An identical elution of proteins was performed in the presence of 650 mM NaCl, followed by a final elution of 1 M NaCl that yielded very few proteins. A large majority of proteins observed in the 1 M elution were found in the 650 mM elution. Therefore, the 1 M elution fractions were omitted from further analysis. Samples were desalted and concentrated using a chloroform/methanol precipitation [17] followed by re-suspension in 0.2 ml 9 M Urea/4% CHAPS. Subsequently, Bradford assays (Pierce, Rockford, IL.) were performed to determine the new protein concentration.

### Two-dimensional electrophoresis

Samples containing 75–200 μg of fractionated cytoplasmic proteins were diluted in 2D electrophoresis buffer (2 M thiourea, 5 M urea, 0.25% CHAPS, 0.25% Tween-20, 0.25% SB-3, 10% isopropanol, and 12.5% water-saturated butanol) containing 15 mg DTT and 0.5% ampholytes (pI ranges 3–10 or 5–8; Bio-Rad; Hercules, CA) to a final volume of 0.2 ml, and passively absorbed onto immobilized pH gradient strips (pI ranges 5–8 or 3–10 NL (non-linear); Bio-Rad) for 4 hours at 20°C followed by active rehydration (50 V at 20°C) for 8 hours. Isoelectric focusing was performed for 35,000 V-h on a Protean IEF System (Bio-Rad), electrophoresis with the Criterion gel system (Bio-Rad). 2D gels were stained with colloidal Coomassie or silver and imaged on a VersaDoc 4000 (Bio-Rad).

### Analysis and quantification

Gels were analyzed using the PDQuest software version 7.4.0 (Bio-Rad). Selected spots from individual gel images were first matched to at least two other replicate gels at each time point using the 'classic gel match' algorithm function. Refined maps for individual time points were created through use of landmarking and manual matching. Once a master gel was created for each time point, higher analysis set matching was performed to identify qualitative changes. Quantitative changes were determined by normalization followed by matching triplicate samples from individual time points to other time points. For each analysis, the 2D gel from the unstimulated C2C12 sample was set as the standard. Based on these settings, 'Analysis Sets' for each of the individual time points was created for a representative gel using the normalized parameters to identify qualitative changes and a 2-fold increase/decrease to identify quantitative changes. Subsequently, proteins that consistently displayed a 3-fold or greater increase or decrease in expression in the cytosol over three separate experiments were validated and reported.

### MALDI-TOF

Spots containing proteins of interest were excised into 96 well plates using an integrated ExQuest spot cutter (Bio-Rad). Spots were washed three times in 50% acetonitrile/10 mM NH_4_HCO_3 _followed by a brief dehydration in 100% acetonitrile. Proteins were incubated overnight in 10% acetonitrile/10 mM NH_4_HCO_3 _with 0.05 μg trypsin (Roche; Indianapolis, IN). Isolated peptides were washed and concentrated in C_18 _ZipTips (Millipore; Billerica, MA) according to the manufacturer's protocol. Samples were directly eluted onto a 100 spot platform using a buffer containing 4-alpha hydroxy cinnamic acid (Agilent; Santa Clara, CA), 50% acetonitrile, 10 mM diammonium citrate, and 0.1% trifluoroacetic acid. MALDI-TOF and subsequent MS/MS analysis of specific peptides was performed on an Applied Biosystems Q-Star XL hybrid mass spectrometer. MALDI-TOF fingerprint data was analyzed with the online database at Rockefeller University .

## Results

### 1. Fractionation of cytoplasmic proteins decreases 2D map complexity and increases the total number of visualized proteins

The serum-depleted C2C12 cells were treated with a single treatment of IGF-1 designed to mimic a therapeutic dose as described in Materials and Methods, fractionated into cytosolic (C), nuclear (N), and detergent-insoluble (I) fractions, and the expression profile was analyzed. These early time points were chosen to identify the most robust and reproducible acute effects of IGF-1 and to avoid the likely multiple genetic and metabolic effects of enhanced cell growth, replication, and differentiation. In control experiments, cells that remained in low serum for the 24 hour IGF-1 treatment did not show significant differences in protein expression from cells isolated at the beginning of the treatment, indicating that the potential effects of C2C12 differentiation were minimal. Our initial findings from gene expression studies, identified marked effects of IGF-1 on major steroid and fatty acid pathways, suggesting that prolonged effects seen after long term IGF-1 exposure may result from complex interactions between multiple genetic, proteomic and metabolic pathways that will be difficult to interpret at later times. Although all three fractions showed numerous changes in protein expression, we report here the alerations only in soluble proteins. To facilitate analysis of the protein mixtures, particularly the components of the nuclear and detergent-insoluble fractions, we have used a new protein fractionation method (Methods and Materials). Protein recovery after batch ion-exchange and desalt/concentration was greater than 95% as measured by BCA assay (data not presented). To test the possibility of selective protein depletion of subclasses of proteins, SDS-PAGE followed by silver staining and quantitation by Quantity One (Bio-Rad) was used to examine C2C12 cytoplasmic proteins before and after protein desalting and concentration (Additional File 1). The general protein patterns of samples before and after were very similar, suggesting that little significant selective protein losses had occurred under our experimental conditions. These data indicate that batch ion-exchange of cytoplasmic proteins provides a reproducible method to simplify sample preparation for 2D electrophoresis analysis of complex protein mixtures.

### 2. Generation of a protein expression profile of soluble proteins from C2C12 cells

After prolonged (24 hour) incubation with IGF-1, many C2C12 cells displayed dramatic changes in morphology and became detached from the cell culture dish, suggesting a major degree of cell loss and death through still uncharacterized mechanisms. Cells at this stage were not included in the subsequent analysis. Protein expression was normalized to the total number of valid spots in each gel using PDQuest and composite expression maps were generated for cytosolic proteins eluted with 0, 200, 400, or 650 mM NaCl.

**Figure 1:**
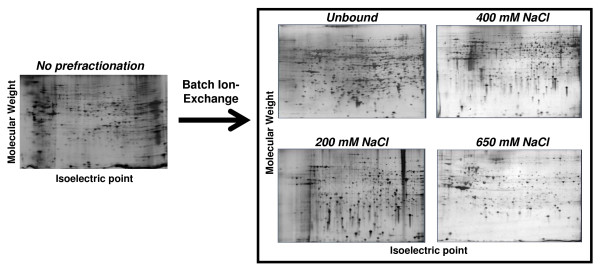
**Batch Ion-Exchange of soluble C2C12 proteins increases the total number of spots visualized 2.5-fold**. Serum-starved C2C12 cells were stimulated for 2 hours with 100 nM IGF-1 and lysed in detergent-free buffer to generate soluble proteins. 100 ug of protein was separated by 2D electrophoresis followed by silver staining (left). Another 100 ug of protein was further fractionated by batch ion-exchange followed by desalting/concentration [17] before 2D electrophoresis and silver staining. The total amount of protein detected was analyzed using PDQuest software (Bio-Rad).

As shown in Figure 1, approximately 700 to 1300 distinct spots were visualized on gels of each fraction, with an average of 850 spots per gel. The results from analysis study provide a molecular fingerprint of the positional location of proteins expressed in C2C12 cells. One hundred and thirty-six spots were selected from the 0 mM elution, 200 mM elution, 400 mM elution, and 650 mM elution of soluble C2C12 lysates excised, prepared for mass spectrometry, and analyzed on a Q-Star XL-hybrid mass spectrometer (Applied Biosystems) using MALDI-TOF as described in Methods. Approximately 4% of all visualized proteins from the combined supernatant gels were initially used to create the expression profile and are listed in **Table S1**; Additional File 2. The proteins are divided into their corresponding functional classes. Additional Files 3, 4, 5, and 6 show the position of all proteins identified in this study. **Table S2**; Additional File 7 provides all MALDI TOF data generated for each protein. PDQuest was used to determine whether a population of the identified landmark proteins remained constant throughout each experiment. Fifty two proteins that were significantly isolated from other proteins on 2D gels were selected and total and normalized values for the protein were compared to each other in three replicate experiments. In each case, the variance of each landmark protein did not change relative to itself or the total normalized value of all valid spots within the dataset. Taken together, these results indicate that the landmark proteins did not change relative to treatment with IGF-1 in triplicate samples.

**Figure 2:**
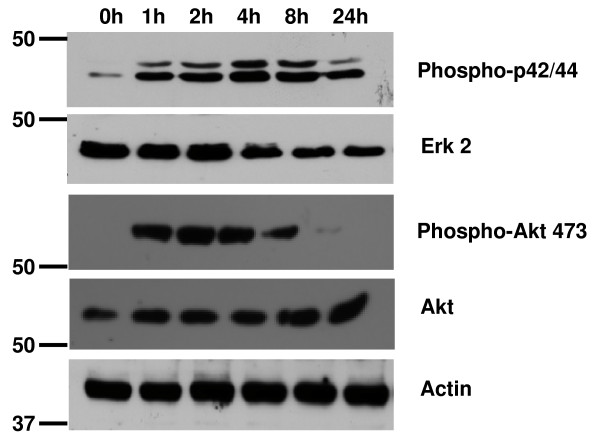
**IGF-1 treatment of C2C12 cells activates MAP kinase and PI 3-kinase signaling pathways**. C2C12 cells were serum-starved for 18 hours prior to stimulation with IGF-1 (100 nM) for 0, 1, 2, 4, 8, or 24 hours. Cells were lysed in detergent containing buffer and 20 ug of protein from each time point was separated on a 10% SDS polyacrylamide gel. Western blots were performed using phosphospecific antibodies to Erk (p42/p44) and Akt (p473) as well as antibodies that detect total protein. Actin is shown as a loading control.

To identify proteins whose expression was up- or down-regulated by IGF-1 treatment, triplicate samples of cytoplasmic proteins from duplicate preparations of serum-starved C2C12 cells incubated with IGF-1 for 0, 1, 2, 4, or 8 hours and fractionated proteins were separated by 2D electrophoresis and visualized by silver staining. To confirm effective IGF-1 stimulation, C2C12 cell lysates were probed for activation of signaling proteins downstream of the IGF-1 receptor that are known to be activated by IGF-1 (Figure 2). In serum-deprived cells, activation of both MAPK and Akt were effectively suppressed, as determined by Western blotting for phospho-Erk and phospho-Akt 473. Upon incubation with IGF-1, cells become activated and display increased Erk and Akt phosphorylation. The degree of phosphorylation is sustained over 8 hours and then both Erk and Akt phosphorylation decrease by 24 hours.

**Figure 3:**
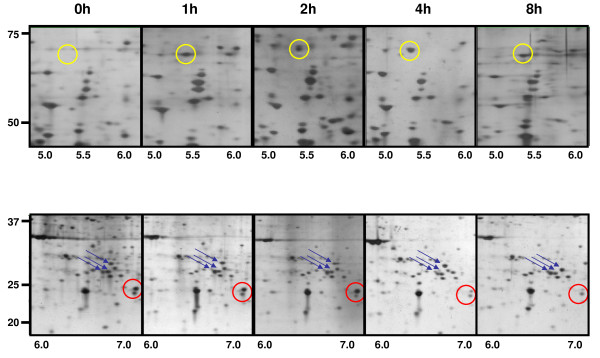
**Analysis of protein expression changes during IGF-1 stimulation**. A) Visualization of significant and reproducible changes in protein expression in IGF-treated C2C12 analyzed as above. *(Top panel) *Expression of proteins highlighted by a yellow circles increase after an 8 hour treatment with IGF-1. *(Bottom panel) *Proteins with red circles are ones that disappear after 8 hour IGF-1 treatment, while proteins within the blue arrows shift position in response to IGF-1 stimulation (ie, possible post-translational modifications including phosphorylation, acetylation, methylation).

Figure 3 demonstrates that IGF-1 treatment of C2C12 cells induced numerous changes in protein expression in each of the four fractions. To illustrate such changes, the data from the 200 mM NaCl elution of proteins in the cytoplasmic fraction of C2C12 cells are shown. Overall, the amounts of most of the observed proteins did not change after IGF-1 treatment of C2C12 cells. A minority of proteins demonstrate increases or decreases. PDQuest analysis of selected spots followed by generation of mass spectrometric data allowed us to identify proteins that were quantitatively increased (Table 1) or decreased (Table 2) after IGF-1 stimulation for each of the four elution conditions. Figure 3 represents a magnified region of two different sections of 2D gels at each of the 5 time points and demonstrates the most frequently observed types of changes in protein expression analyzed in these studies. In the top panels (yellow circles), expression of an unidentified protein appears after 1 hour and is sustained for 8 hours after IGF-1 treatment. In the bottom panels, expression of an unidentified protein decreases after IGF-1 treatment (red circle). Additionally in these panels, shifts in both the intensity and localization of a group of proteins are observed (blue arrows). Each of these spots was identified as the molecular chaperone Mot66. During the time course of the experiment, the intensity of the individual spots changes with one spot appearing after 2 hours with IGF-1 (marked with an arrow). The observed shifts in position of proteins could represent splice variants and/or posttranslational modifications rather than increases or decreases in absolute amounts of proteins These changes represent a significant challenge to the analysis and interpretation of the data (see discussion).

**Table 1:** Proteins whose expression is consistently increased after 8 hour treatment with IGF-1.

**Spot Number**	**Protein Name**	**NCBI gi #**	**Fold Increase**	**SC (%)**	**Z-score**	**MW; pI (theoretical)**	**MW; pI (actual)**
	**Adaptor Protein:**						

1	CASK interacting protein 1	161168996	3.8 ± 0.2	11	2.29	141.42/9.5	120/8.5

2	Wbscr1 protein	15928652	3.9 ± 0.4	41	1.93	25.22/7.8	27/7.5

	**Cell surface receptor:**						

							

3	Stabilin 1	26352640	4.0 ± 0.4	17	2.09	49.21/8.0	55/8.0

	**Cytoskeletal/Structural:**						

							

4	GRIP and coiled-coil domain-containing protein 2	32469711	3.2 ± 0.1	12	1.75	195.40/5.1	170/5.8

5	Rho interacting protein 3	56205310	3.3 ± 0.1	12	1.27	115.70/6.0	140/6.5

6	Vimentin	55408	5.3 ± 0.6	23	1.93	53.72/5.1	55/5.6

							

	**DNA Binding protein:**						

7	RAD50	1575575	5.0 ± 0.4	14	1.59	154.61/6.5	80/7.8

							

	**Enzyme: Hydratase**						

8	Enolase (phosphopyruvate hydratase) (EC 4.2.1.11) alpha	109803	4.7 ± 0.3	29	1.90	47.45/6.4	50/5.7

							

	**Enzyme: Isomerase**						

9	Triose-phosphate isomerase	1363194	3.8 ± 0.1	27	2.01	27.02/6.9	30/7.5

							

	**Enzyme: Sulphotransferase:**						

10	N-deacetylase/N-sulfotransferase 4	11344505	3.7 ± 0.3	10	1.74	101.24/6.9	100/6.5

							

	**Extracellular matrix:**						

11	Collagen alpha-1(XVII) chain	41688459	3.5 ± 0.4	8	1.38	148.48/9.0	160/7.2

							

	**Heat shock protein (Chaperone):**						

12	Heat shock protein 8	42542422	3.3 ± 0.2	21	1.39	71.08/5.3	78/5.3

13	Heat shock protein A/p66 mot	34784211/5736598	4.1 ± 0.4	14		73.80/5.9	62/5.2

							

	**Phosphotransferase:**						

14	FLT-1 (Tyrosine-protein kinase receptor FLT)	2809069	3.1 ± 0.2	8	1.53	151.71/9.1	160/10

15	Ikbkb protein	22902306	3.6 ± 0.4	22	1.60	87.91/5.7	80/7.8

							

	**Reductase:**						

16	pyrroline-5-carboxylate reductase-like	119508439	4.1 ± 0.4	27	1.32	29.13/6.8	36/7.9

							

	**Ribonuclease:**						

17	Plenty-of-prolines-101; POP101; SH3-philo-protein	3153821	5.6 ± 1.0	20	1.68	101.21/11.9	110/6.2

							

	**Transcription factor:**						

18	NFKB-repressing factor-Transcription factor NRF	12861044	3.0 ± 0.1	13	1.49	77.71/9.1	33/4.5

							

	**Unclassified/Unknown function:**						

19	Cep290	26353954	4.1 ± 0.3	33	2.08	93.89/7.6	95/7.6

20	mKIAA0209 protein/dedicator of cytokinesis 2	50510415	3.2 ± 0.1	16	1.45	182.35/6.7	125/7.5

21	mKIAA1486 protein	81896693	3.0 ± 0.3	13	1.95	71.19/9.1	63/8.5

22	nasopharyngeal epithelium specific protein 1		3.4 ± 0.1	30	2.08	65.98/9.0	75/9.0

23	progesterone-induced blocking factor 1 isoform a	46852193	3.3 ± .03	21	1.73	89.88/5.9	90/6.8

Note 1: Protein classification is according to the Human Protein Reference Database .

Note 2: SC%: Percentage of sequence coverage.

**Figure 4:**
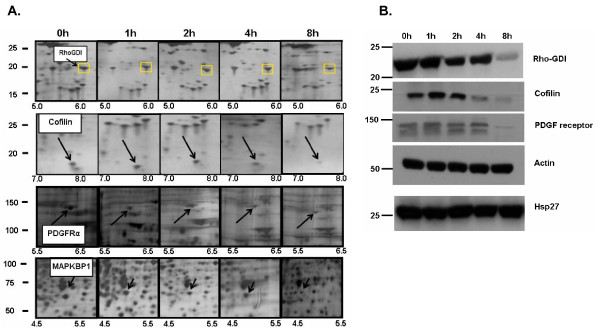
**Expression of Rho-GDI, Cofilin, PDGF receptor α, and MAPKBP-1 decreases upon IGF-1 stimulation of C2C12 cells**. A) Triplicate samples from three different C2C12 cell preparations were stimulated with IGF-1 (100 ng/ml) for up to 8 hours, fractionated using batch ion-exchange, separated by 2D electrophoresis, and proteins were detected with silver stain. The protein highlighted by a yellow square (Rho-GDI, Cofilin, PDGF receptor α, and MAPKBP-1) decrease in expression after IGF-1 treatment. PDQuest analysis indicates a quantitative change in the protein based on total quantitation of the entire gel. B) Western blot analysis confirms the sustained decrease in protein expression for Rho-GDI, cofilin, and the PDGF receptor.

**Figure 5:**
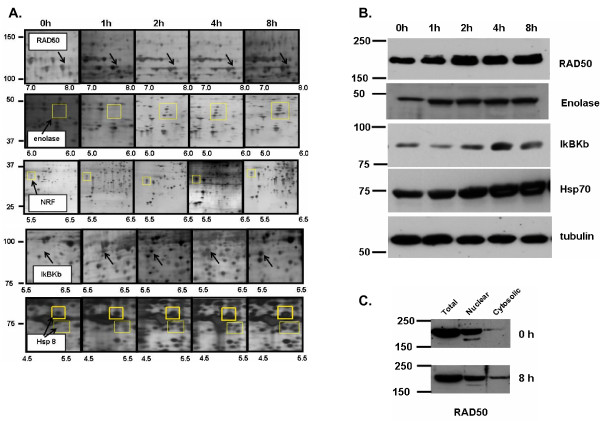
**Expression of Rad50, enolase, IκB kinase b, and Hsp70 increases upon IGF-1 stimulation of C2C12 cells**. A) Triplicate samples from three different C2C12 cell preparations were stimulated with IGF-1 (100 ng/ml) for up to 8 hours, fractionated using batch ion-exchange, separated by 2D electrophoresis, and proteins were detected with silver stain. The protein highlighted by a yellow square (Rad50, enolase, IκB kinase b, and Hsp70) increase in expression after IGF-1 treatment. PDQuest analysis indicates a quantitative change in the protein based on total quantitation of the entire gel. B) Western blot analysis confirms the sustained increase in protein expression for Rad50, enolase, IκB kinase b, and Hsp70. C) RAD50 accumulates in the cytosolic fraction of IGF-1 stimulated C2C12 cells.

**Figure 6:**
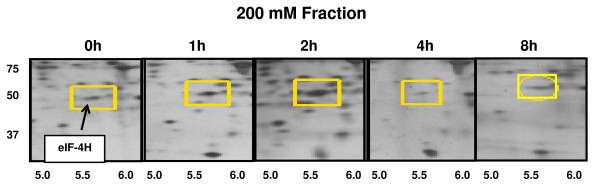
**Triplicate samples from three different C2C12 cell preparations were stimulated with IGF-1 (100 ng/ml) for up to 8 hours, fractionated using batch ion-exchange, separated by 2D electrophoresis, and proteins were detected with silver stain**. The protein highlighted by a yellow square (eIF-4H) rapidly increases in expression after 1 hour IGF-1 treatment, but then decreases at 4 and 8 hours.

Note 3: Spot Number refers to the location of the individual proteins on 2D gels as shown in Figures 3–6. When a protein was identified in more than one Batch IEX fraction, it is marked multiple gels.

Note 4: Supplemental Table 2 contains all MALDI TOF data generated for each protein.

Note 5: Only proteins with probability score of 1.0e+000 were included in the table.

**Table 2:** Proteins whose expression is consistently decreased after 8 hour treatment with IGF-1.

**Spot Number**	**Protein Name**	**NCBI gi #**	**Fold Decrease**	**SC (%)**	**Z-Score**	**MW; pI (theoretical)**	**MW; pI (actual)**
	**Cell surface receptor:**						

24	Platelet-derived growth factor receptor, alpha precursor (PDGF-R-alpha)	2506800	3.8 ± 0.3	11	1.30	123.68/5.0	125/4.8

							

	**Cytoskeletal protein:**						

24A	cofilin	6680924	4.9 ± 0.1	37	2.01	18.55/8.5	25/5.6

							

	**DNA topoisomerase activity:**						

25	Topoisomerase (DNA) II beta	32451610	3.6 ± 0.2	11	1.82	182.80/8.7	140/8.0

							

	**GTPase activating protein:**						

26	Rac GTPase activating protein; GAB-associated CDC42	28569544	3.4 ± 0.3	8	1.62	191.74/6.6	180/7.0

27	Rho GDP-dissociation inhibitor 1	31982030	3.6 ± 0.2	34	2.30	23.41/5.1	30/5.4

							

	**Heat shock protein (Chaperone):**						

28	Heat shock protein 1 (chaperonin)	16741093	5.8 ± 0.2	18	1.46	59.02/5.5	60/5.7

							

	**Protein Phosphatase:**						

29	secreted embryonic phosphatase	16580151	4.1 ± 0.4	25	1.27	55.23/6.3	60/7.8

							

	**Structural constituent of ribosome:**						

30	Stmn1 protein	32449851	3.2 ± 0.3	32	1.60	17.26/5.7	18/7.6

							

	**Transcription factor:**						

31	myelin transcription factor 1-like	6679000	3.9 ± 0.1	20	2.05	137.62/4.8	140/5.2

32	zinc finger protein 100	50510803	4.7 ± 0.4	8	1.99	104.8/9.3	100/8.5

							

	**Transport/Cargo:**						

33	Sorting nexin 13	38614111	3.8 ± 0.2	6	1.13	111.54/6.2	120/7.0

							

	**Ubiquitin proteasome system protein:**						

34	ubiquitin fusion degradation 1 like	31981443	4.3 ± 0.1	22	1.83	34.75/6.0	29/6.4

35	Ubiquitin-conjugating enzyme E2 N	46577661	3.2 ± 0.1	24	2.22	17.14/6.1	18/6.5

							

	**Unclassified/Unknown function:**						

36	Mapkbp1	47124622	4.0 ± 0.1	9	1.11	164.70/6.3	60/7.5

37	Phosphatidylethanolamine-BP 1	1517864	3.2 ± 0.3	17	2.31	20.83/5.2	22/5.2

38	Stromal antigen 2	41940887	3.8 ± 0.1	21	1.66	135.13/5.2	190/6.2

39	Tpr	14329713	3.7 ± 0.2	18	1.40	139.30/5.3	110/5.3

Note 1: Protein classification is according to the Human Protein Reference Database .

Note 2: SC%: Percentage of sequence coverage.

Note 3: Spot Number refers to the location of the individual proteins on 2D gels as shown in Figures 3–6. When a protein was identified in more than one Batch IEX fraction, it is marked multiple gels.

Note 4: Supplemental Table 2 contains all MALDI TOF data generated for each protein.

Note 5: Only proteins with probability score of 1.0e+000 were included in the table.

Figure 4A shows changes in protein expression of four selected proteins from different regions of 2D gels from the 200 mM NaCl elution. Western blot analysis of Rho-GDI, cofilin, and PDGF receptor α from cell extracts from IGF-1-stimulated cells (Figure 4B, **top panels**) confirm the decreased expression after IGF-1 treatment identified from the mass spectroscopic analysis. As a control, Western blots for two proteins whose expression remained constant during the 8 hour IGF-1 treatment (actin and Hsp27) are shown (Figure 4B, **bottom panels**). When quantified, data from western blots indicate Rho-GDI expression levels decrease 5.1-fold, cofilin levels drop 8.3-fold, and PDGF receptor α levels decrease by 4-fold over the 8 hour time course. These observed decreases in protein expression are similar to those observed in the 2D gels. Averaged analysis of the spots on the 2D gels indicates a 4.3-fold decrease for Rho-GDI, a 6-fold decrease for cofilin, and a 4-fold decrease for PDGF receptor α. Figure 5A shows regions of 2D gels in which expression of other proteins including Rad50, enolase, IκBKb, and Hsp70 increased after treatment with IGF-1. Western blots for these proteins confirmed the change in expression (Figure 5B) when compared with actin and Hsp27 expression (Figure 4B). Rad50 levels increased 2.6 fold, enolase increased 3.2 fold, IκBKb levels increased 3.8 fold, and Hsp70 levels increased 2.3 fold. Again, the fold increase as determined by Western blotting was similar to levels measured by PDQuest on the 2D gels with Rad50 and Hsp70 increasing 2-fold and enolase and IκBKb increasing almost 4-fold.

RAD50 is primarily a nuclear protein and its appearance in the cytosolic fraction of C2C12 cells suggested a number of possibilities could be happening in cells including nuclear lysis, cells undergoing apoptosis/necrosis, or possibly degradation of the protein. To determine whether more RAD50 was in the cytosolic fraction after IGF-1 treatment, Western blot analysis was performed on whole cell lysates, nuclear fractions, and cytosolic fractions (Figure 5C). In the absence of stimulus, there was a small, but reproducible amount of RAD50 in the cytosol. After 8 hours with IGF-1, the amount of RAD50 in the cytosol had dramatically increased. The total amount of the protein detected by Western blot did not appreciably change, indicating that IGF-1 treatment induced a redistribution of RAD50 from the nuclear fraction to the cytosolic fraction. The significance of RAD50 redistribution is addressed in the Discussion.

In addition to these stable alterations of protein expression, our analysis also identified 92 proteins that demonstrated significant but transient and in some cases multi-phasic changes in expression after IGF-1 treatment. One example of this complex kind of changed protein expression is shown in Figure 6 for the transcription factor eIF-4H. At early times up to 2 hours after IGF-1 treatment, the level of eIF-4H protein increases, but then falls over the following 4 hours. The mechanisms responsible for such complex regulation will be important to clarify in subsequent phases of this study.

## Discussion

In these studies, we report initial results of a comprehensive study of the effects of IGF-1 on the proteome of mouse C2C12 myoblast cells in culture. For this analysis we have developed an improved fractionation technique to separate proteins into cytoplasmic, nuclear, and detergent insoluble fractions and then to sub-fractionate the largest population of soluble proteins by batch ion-exchange chromatography into four separate elution batches. The batch elution was followed by desalting and protein concentration, thereby making the proteins amenable to 2D electrophoresis. The present revised sub-fractionation scheme has proven to be effective for increasing the total number of visualized spots in subsequent 2D gel analysis by 3-fold to over 3900. However, as expected with any fractionation technique, multiple spots were mapped to identical positions in different elutions from the Q-Sepherose. When these ~650 spots were removed from the analysis, the net increase in unique spots observed from our fractionation protocol was increased 2.5-fold to over 3200. The loss of protein was minimal in 6 independent experiments using this procedure and our data have not shown a major degree of selective protein loss. Within the 200 mM elution fraction, we identified 23 proteins whose expression increased and 17 proteins whose expression decreased suggesting IGF-1 signaling regulated the transcription of many different genes.

In a parallel series of experiments, changes in the mRNA expression profiles of C2C12 cells was analyzed to determine the effects of IGF-1 on regulation of gene expression. The results of a thorough analysis of all of the microarray data and the expression data did not reveal any strong correlation between the two sets of data (R. Bhasker and T. Friedmann, personal communication). The lack of correlation between microarray and proteomic data has been observed by other groups [18-21] and may be explained by regulated translation, microRNAs, or alterations/regulation of proteosomal or other degradation.

To test the degree of the sample variability that inevitably arises from different cell preparations, we routinely analyzed triplicate gels from three different experiments of IGF-1 stimulation of C2C12 cells at each of the 5 time points. Overall, the levels and positions of 90% of the proteins visualized at a single time point (nine different 2D gels) did not change after IGF-1 treatment. Of the 90% of proteins whose expression remained constant at a single time point, the variability between spots from triplicate samples at different times of IGF-1 treatment was less than 8% as determined using the matching algorithm of PDQuest (data not shown). Taken together, these data suggest that expression of most proteins does not dramatically change upon IGF-1 treatment. It is common in most batch ion-exchange methods for high-abundance proteins to be found in multiple elution fractions protein separation. In our studies, approximately 18% of the proteins observed were seen in multiple gels representing different fractionation pools and not surprisingly, mass spectrometric methods revealed that most such proteins were high abundance proteins such as actin and multiple isoforms of heat shock proteins (data not shown). In 2D electrophoresis and computer aided analytic protocols, elution of the same landmark protein into multiple fractions helps to align very refined 2D maps which can be subsequently be used to track low abundance proteins and post-translational modifications.

A major concern regarding 2D-gel analysis of proteins under these conditions is the likelihood that some apparently single spots will in fact contain different proteins with similar electrophoretic and charge properties. We have been able to obviate that possibility to at least some extent by using the landmarks described in Table 1 to identify distinct proteins the run in similar positions on 2D gels but that elute at different salt concentrations (Additional File 8). Nevertheless, the protein overlap problem was frequently observed in these studies and remains a serious problem in proteomic studies of all kinds, making it likely that many changes in stimulus-induced protein expression may be masked during analysis. Although fraction of complicated samples partially resolves this issue, more advanced separation techniques must be developed to completely address this issue.

The present studies focus on the effect of IGF-1 on changes in expression of fractionated cytosolic proteins. A complicating factor in these studies is that proteins often redistribute from the cytosol to various other intracellular locations including organelles (nucleus, mitochondria), membranes, and cavelolae. Fractionation of these sub-cellular populations of proteins may result in an erroneous identification of a change in expression when in fact the total levels of protein do not change. This is demonstrated in Figure 2 (second panel; Erk2) that redistributed to the nucleus (data not shown) and RAD50 that accumulates in the cytosol (Figure 5C). Whether these changes observed in protein localization represent responses to IGF-1 or to cell processing remains to be determined. However, these findings emphasize the need for a balanced yet rigorous analytical biochemical analysis of candidate protein expression from total cell lysates and multiple fractions. Analysis of total, nuclear, and cytosolic fractions of enolase, IκBKb, PDGF receptor, cofilin, and Rho-GDI identified these proteins as cytosolic and were not found appreciably in the nucleus. Therefore, the change in protein expression is likely due to a direct effect of IGF-1 (data not shown). However, significant amounts of Hsp70 were found in both the nuclear and cytosolic fractions of C2C12 cells. The amount of nuclear Hsp70 did not appreciably decrease as cytosolic levels increased, indicating that changes in expression of this protein also respond to IGF-1. It remains to be determined whether changes in expression of the other proteins identified in this study can be attributed to alterations in subcellular distribution.

The present identification of up- or down-regulated proteins emphasizes the proteins that showed a consistent temporal increase or decrease in expression over the entire time course of these studies. Attempts to determine whether any of these proteins are directly linked to IGF-1 signaling or part of a larger protein/signal transduction network did not yield any plausible links, suggesting proteins whose expression changes in response to IGF-1 remain to be identified. The proteins identified only represent a portion of the changes that we observed in the IGF-1-treated cells. The present report does not include a complete analysis of the proteins that demonstrate complex, multiphasic changes in absolute amount or in their pI or apparent electrophoretic mobility (Figure 3A, **lower panel**). Nevertheless, we have identified a number of proteins that are increased robustly to IGF-1 at early time points (1–2 hours) but that then are subsequently decreased in the later time points and have returned to basal levels by 8 and 24 hours (see Figure 6). These proteins may represent attractive candidates for critical signaling molecules that play important roles in the action of IGF-1. Conversely, other proteins show strong but apparently delayed down-regulation by IGF-1. It remains to be determined if these changes are direct effects of IGF-1 or are the result of secondary metabolites, are cell death or loss or other mechanisms. Mass spectrometry analysis of a number of these proteins indicates that they represent modified variants of the same protein (data not shown). Our future studies will be aimed at determining to what extent these variations represent splice variants or arise from post-translational protein modifications. Alterations to the focusing patterns on 2D gels at specific time points during IGF-1 treatment may help identify changes in protein expression, subcellular localization, and/or signaling.

Two other laboratories have examined the proteome of C2C12 cells, one using a 2D electrophoresis/MALDI MS approach [22], the other using a LC MS/MS approach [23]. The focus of both studies was to identify proteins in C2C12 cells that have regulated expression during muscle development. In the first study, Tannu *et al. *identified the positional location of proteins in 2D gels whose expression changed during myogenic differentiation. Given the different experimental conditions and focus on landmark proteins in the current study, it is not surprising that many of the 106 proteins identified by Tannu were not identified here. In the second study, Kislinger et al. used a LC MS/MS approach to identify proteins that change in expression upon C2C12 differentiation [23]. Comparing proteins identified in the Tannu and Kislinger studies (see Kinlinger Supplemental Data, p.940) to our study finds that actin, 60 kD heat shock protein, and mitochondrial ATP synthase levels remain constant. Abundant proteins often identified in many studies and cell types, including actin, myosin heavy/light chain, protein disulfide isomerases, glucose regulated protein, and ATP synthase subunits were also identified in all three studies. Interestingly, the expression levels of these proteins remained constant during the 8 hour time course in our studies, but all increased during the 5 day differentiation protocol used by Tannu and Kislinger. Additionally, in our studies, we identified vimentin, enolase, and heat shock protein 8 as proteins whose expression increased during an 8 hour treatment with IGF-1. The same proteins identified by Tannu, decreased (vimentin) or remained the same during myogenic differentiation. Whether these differences represent changes that are specifically initiated during differentiation or during IGF-1 stimulation remains to be determined.

In their previous studies, Tannu and Kislinger specifically tracked individual proteins that are up- or down-regulated by IGF-1 revealed a mixture of enzyme classes [22,23]. As in our present study, the changes in the levels and properties of these proteins may be related to both cell culture conditions and post-translational modifications. Of particular interest are the identification in this study of the up-regulation of PDGF by IGF-1 and the identification in another study of the regulation of PDGF expression by insulin in C2C12 cells [24]. Because of the similarities in signal responses to insulin and IGF-1, it seems possible that this growth factor is an important target for IGF-1 action in these cells. In addition, we are particularly interested to examine further the changes in the levels of several cytoskeletal regulators such as Rho-GDI and cofilin, a protein associated with the cytoskeleton that binds actin and reversibly controls polymerization and depolymerization in a pH-sensitive manner. The ability of cofilin to regulate actin polymerization is known to be regulated by reversible phosphorylation, suggesting this protein may also be a candidate target protein for IGF-1 action.

Traditional approaches to detection and identification of performance-enhancing substances and methods have generally focused on assays for the performance-enhancing substances themselves. However, a potentially more powerful approach may involve the identification of qualitative and quantitative drug-induced signature changes in gene and protein expression and in the metabolome of treated tissues. Development of a panel of such biomarkers that collectively can be shown to represent a reliable signature for exposure to exgenous IGF-1 or other agents such as a variety of growth hormones, steroids, etc., could represent a useful addition to the anti-doping effort.

## Competing interests

The authors declare that they have no competing interests.

## Authors' contributions

CK conceived and designed the study, designed the experiments, performed the mass spectrometry, Western blot, statistical analysis and manuscript draft preparation. KB performed the 2D gels and spot analysis. TF conceived and designed the study and drafted the manuscript. All authors edited the manuscript and approved the final version.

## Supplementary Material

Additional file 1 **Silver stain of cytosolic, membrane, and detergent insoluble proteins before and after desalting and concentration**. The data provided represent the fidelity of recovery of proteins after desalting and concentrating. Click here for file

Additional file 2 **Landmark proteins identified in fractionated C2C12 cells**. The data provided represent MALDI-TOF information from each protein identified in these studies. Click here for file

Additional file 3 **Silver stain of a representative 2D gel from the unbound elution of unstimulated C2C12 cells**. The data provided show the positional location of proteins identified by MALDI TOF from the unbound elution of unstimulated C2C12 cells. Click here for file

Additional file 4 **Silver stain of a representative 2D gel from the 200 mM elution of unstimulated C2C12 cells**. The data provided show the positional location of proteins identified by MALDI TOF from the 200 mM elution of unstimulated C2C12 cells. Click here for file

Additional file 5 **Silver stain of a representative 2D gel from the 400 mM elution of unstimulated C2C12 cells**. The data provided show the positional location of proteins identified by MALDI TOF from the 400 mM elution of unstimulated C2C12 cells. Click here for file

Additional file 6 **Silver stain of a representative 2D gel from the 650 mM elution of unstimulated C2C12 cells**. The data provided show the positional location of proteins identified by MALDI TOF from the 650 mM elution of unstimulated C2C12 cells. Click here for file

Additional file 7 **MALDI-TOF data from each protein identified in these studies**. The data provided represents all MALDI-TOF information from each protein identified in these studies. Click here for file

Additional file 8 **BIEX helps resolve cytosolic proteins with similar isoelectric points and apparent molecular weight**. The data provided show that proteins that focus at the same molecular weight and isoelectric point can be resolved after batch ion exchange chromatography. Click here for file
