# Supplementary figures and images for: A fractionation method to identify qauntitative changes in protein expression mediated by IGF-1 on the proteome of murine C2C12 myoblasts
Source: Proteome Sci. 2009 Aug 11;7:28. doi: 10.1186/1477-5956-7-28 (PMC2732595; doi:10.1186/1477-5956-7-28)

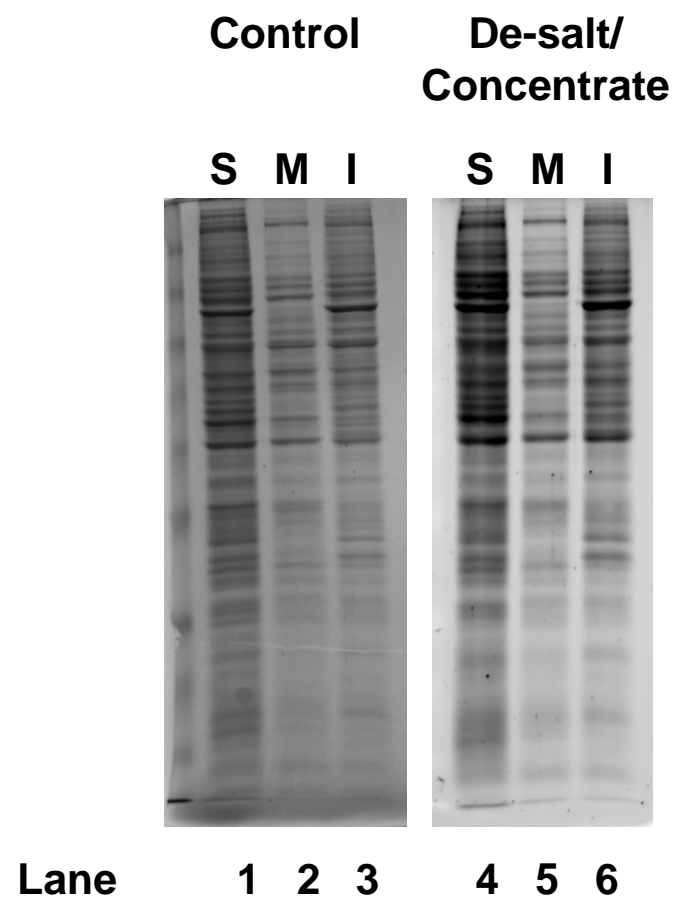

Supplement: Additional file 1 — Silver stain of cytosolic, membrane, and detergent insoluble proteins before and after desalting and concentration. The data provided represent the fidelity of recovery of proteins after desalting and concentrating. [file 1477-5956-7-28-S1.pdf]

**Molecular Weight**

***Flow Through***

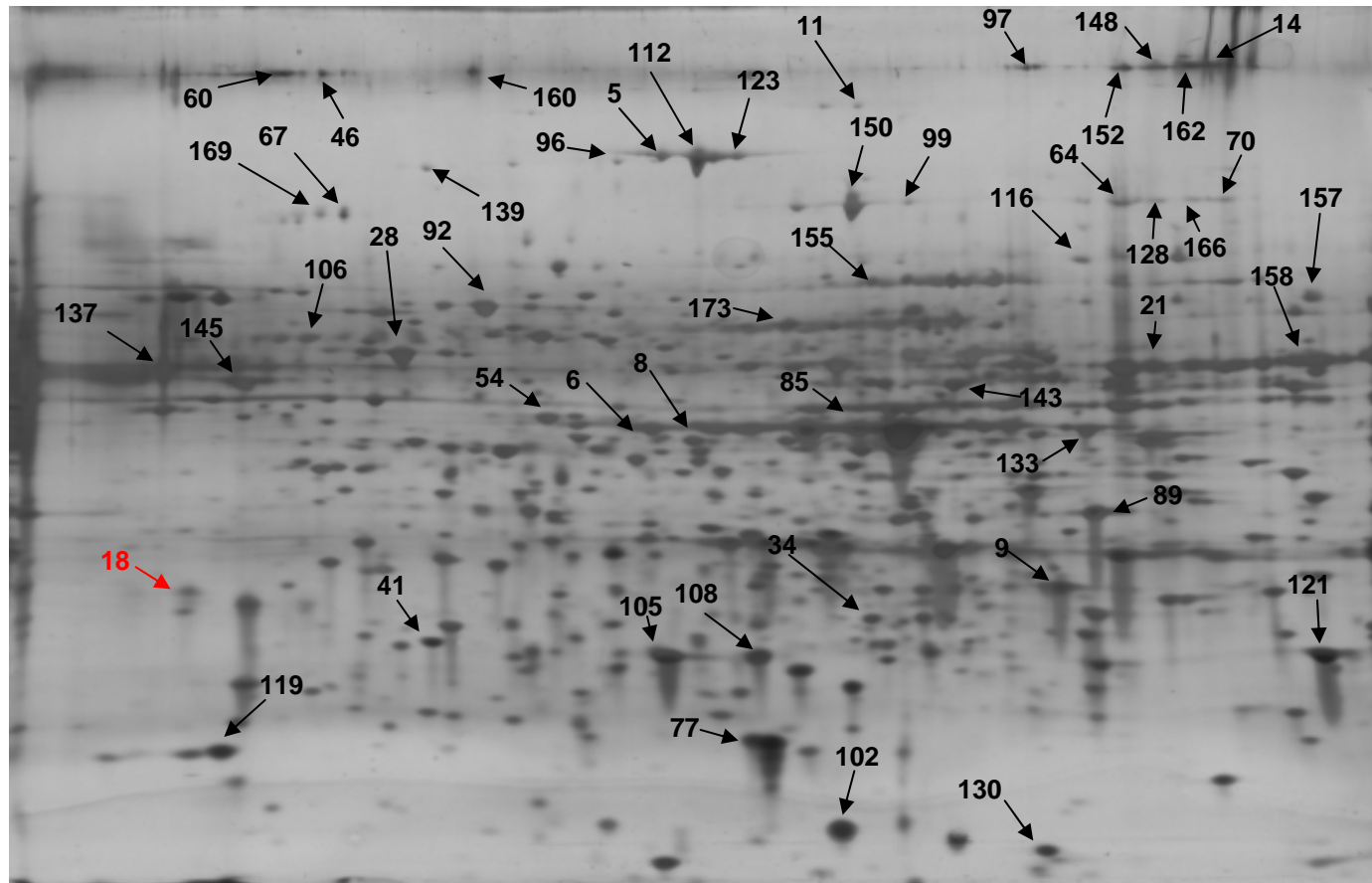

**Isoelectric point**

Supplement: Additional file 3 — Silver stain of a representative 2D gel from the unbound elution of unstimulated C2C12 cells. The data provided show the positional location of proteins identified by MALDI TOF from the unbound elution of unstimulated C2C12 cells. [file 1477-5956-7-28-S3.pdf]

**Molecular Weight**

**200 mM NaCl**

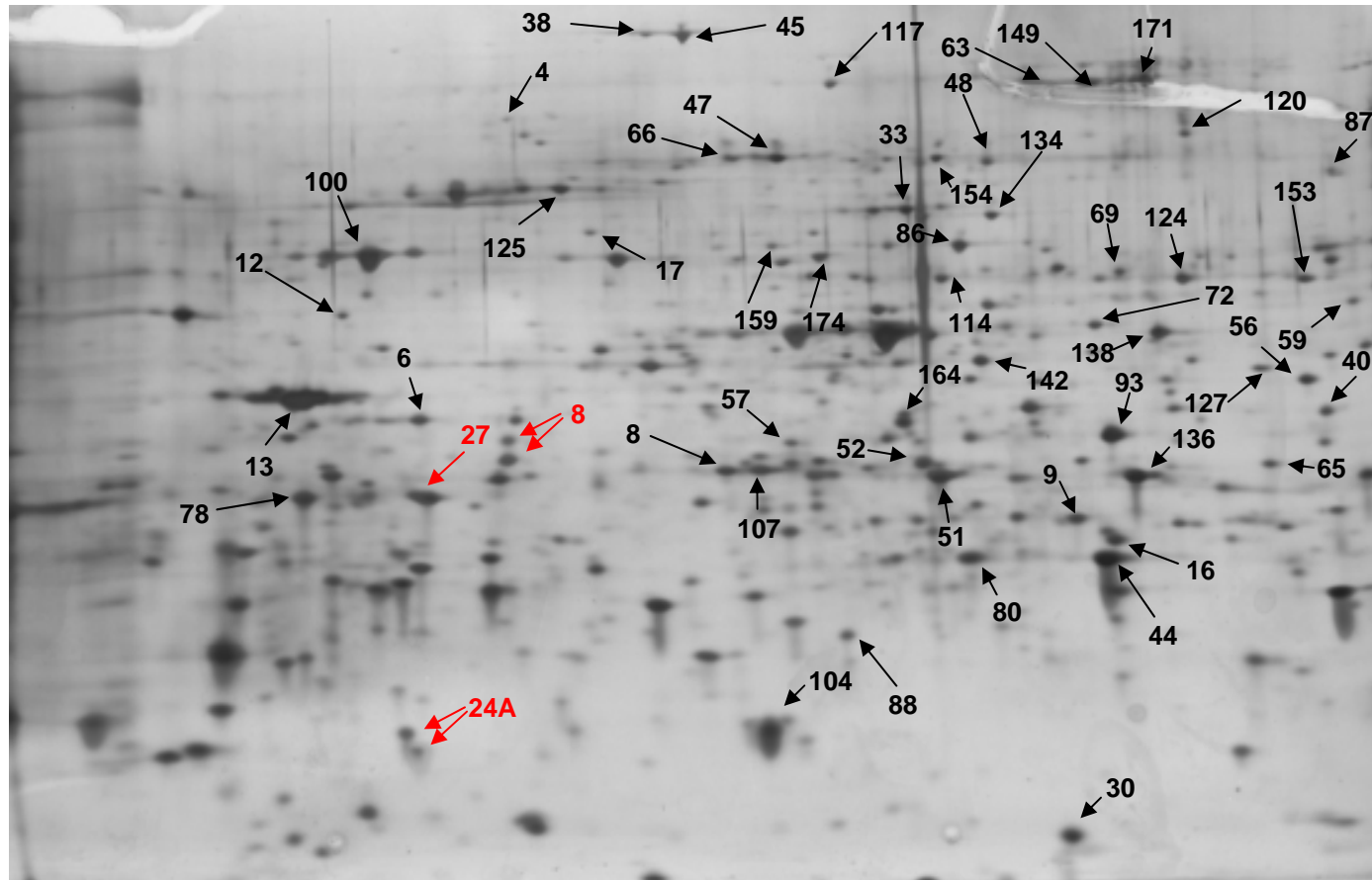

**Isoelectric point**

Supplement: Additional file 4 — Silver stain of a representative 2D gel from the 200 mM elution of unstimulated C2C12 cells. The data provided show the positional location of proteins identified by MALDI TOF from the 200 mM elution of unstimulated C2C12 cells. [file 1477-5956-7-28-S4.pdf]

*400 mM NaCl*

**Molecular Weight**

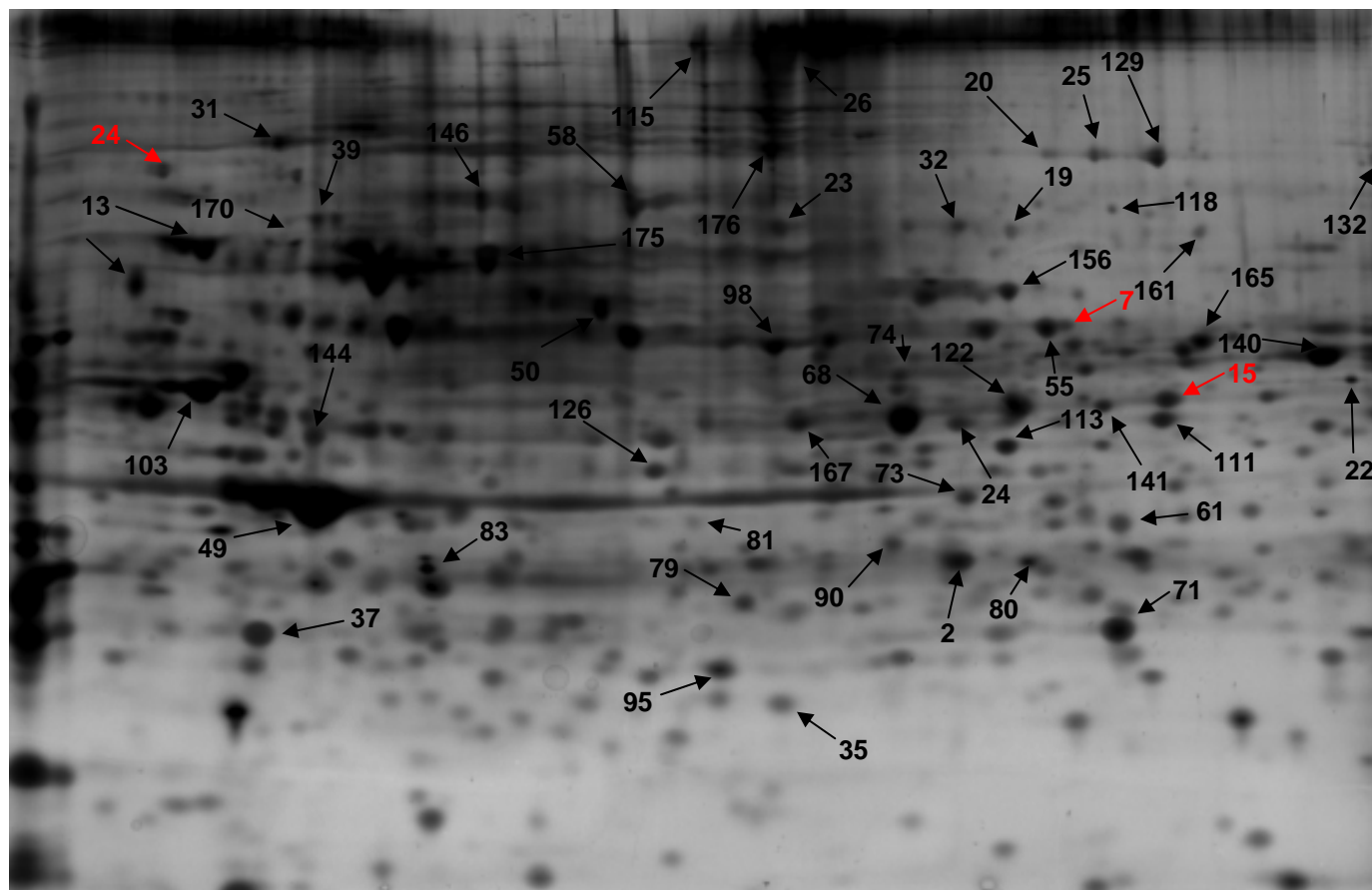

**Isoelectric point**

Supplement: Additional file 5 — Silver stain of a representative 2D gel from the 400 mM elution of unstimulated C2C12 cells. The data provided show the positional location of proteins identified by MALDI TOF from the 400 mM elution of unstimulated C2C12 cells. [file 1477-5956-7-28-S5.pdf]

**Molecular Weight**

**650 mM NaCl**

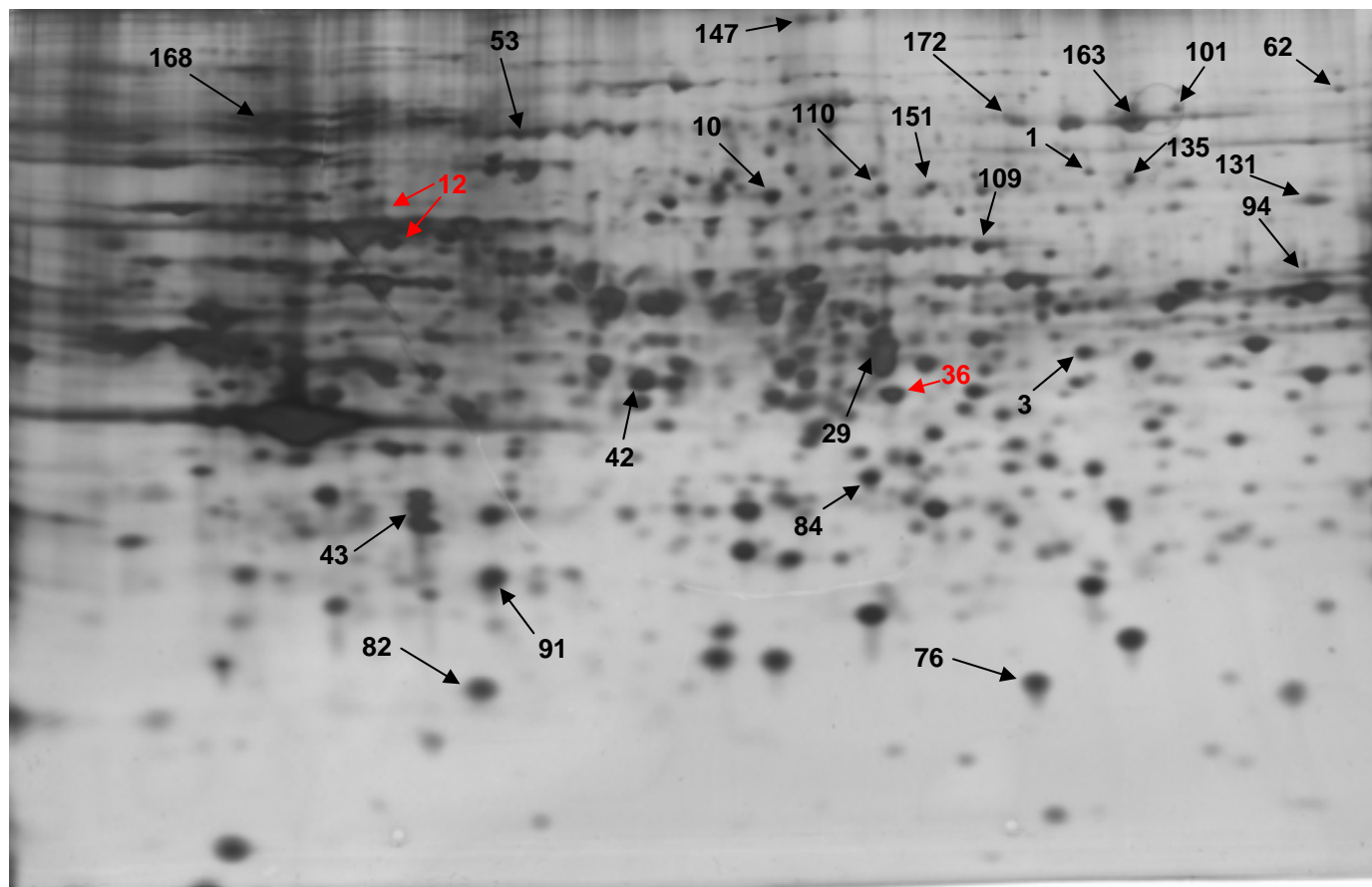

**Isoelectric point**

Supplement: Additional file 6 — Silver stain of a representative 2D gel from the 650 mM elution of unstimulated C2C12 cells. The data provided show the positional location of proteins identified by MALDI TOF from the 650 mM elution of unstimulated C2C12 cells. [file 1477-5956-7-28-S6.pdf]

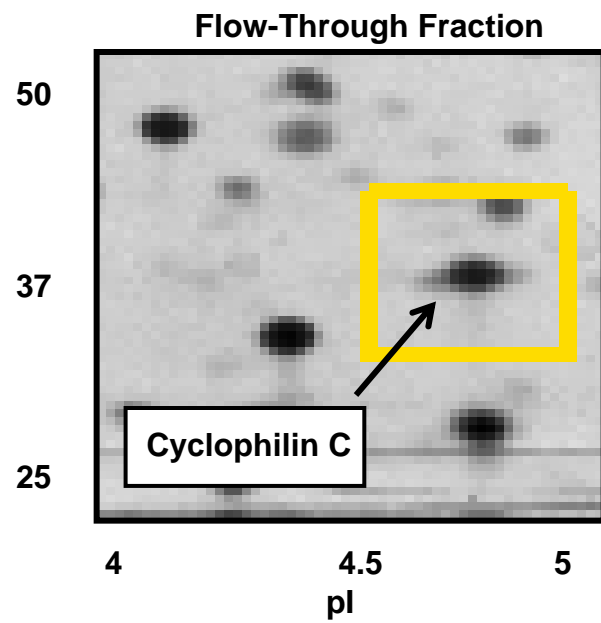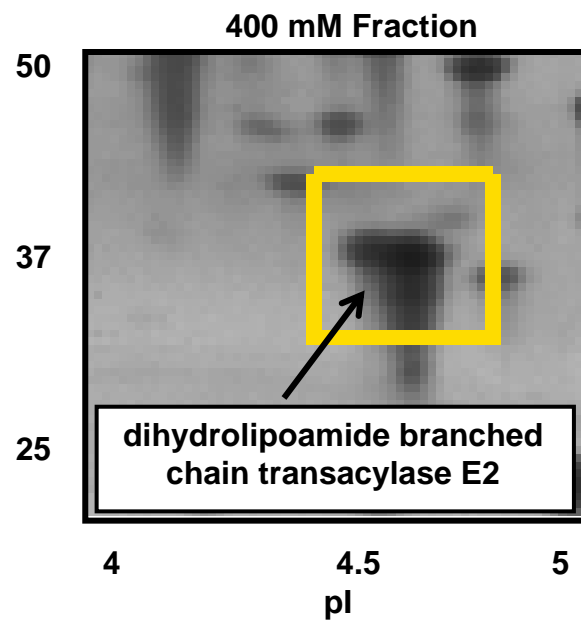

Supplement: Additional file 8 — BIEX helps resolve cytosolic proteins with similar isoelectric points and apparent molecular weight. The data provided show that proteins that focus at the same molecular weight and isoelectric point can be resolved after batch ion exchange chromatography. [file 1477-5956-7-28-S8.pdf]
