# Supplementary material for: A fractionation method to identify qauntitative changes in protein expression mediated by IGF-1 on the proteome of murine C2C12 myoblasts
Source: Proteome Sci. 2009 Aug 11;7:28. doi: 10.1186/1477-5956-7-28 (PMC2732595; doi:10.1186/1477-5956-7-28)
Supplement: Additional file 2 — Landmark proteins identified in fractionated C2C12 cells. The data provided represent MALDI-TOF information from each protein identified in these studies. [file 1477-5956-7-28-S2.doc]

**Additional File 2.** Landmark proteins identified in fractionated C2C12 cells

| **Spot Number** | **Protein Name** | **NCBI gi #** | **SC (%)** | **Z-score** | **MW; pI (theoretical)** | **MW; pI (actual)** |
| --- | --- | --- | --- | --- | --- | --- |
|  | **Acyltransferase:** |  |  |  |  |  |
| 40 | Dihydrolipoamide branched chain transacylase E2 | 6753610 | 15 | 1.99 | 53.48/9.1 | 55/8.9 |
|  |  |  |  |  |  |  |
|  | **Adaptor Protein:** |  |  |  |  |  |
| 41 | Prohibitin | 541732 | 38 | 2.23 | 29.86/5.6 | 25/5.2 |
|  |  |  |  |  |  |  |
|  | **Aminotransferase:** |  |  |  |  |  |
| 42 | Ornithine aminotransferase | 14198116 | 24 | 1.99 | 48.74/6.2 | 50/6.3 |
|  |  |  |  |  |  |  |
|  | **Calcium Binding Protein:** |  |  |  |  |  |
| 43 | Annexin A3 | [5902786](http://www.ncbi.nlm.nih.gov/htbin-post/Entrez/query?form=6&db=p&Dopt=g&uid=5902786) | 36 | 2.32 | 36.52/5.3 | 36/5.2 |
| 44 | lipocortin I | 113945 | 36 | 2.15 | 39.00/7.0 | 35/7.7 |
|  |  |  |  |  |  |  |
|  | **Cell Adhesion/Junction:** |  |  |  |  |  |
| 45 | Cingulin | 38077877 | 15 | 2.31 | 150.71/5.9 | 180/6.3 |
| 46 | Nrxn3 protein | 38173745 | 9 | 1.55 | 177.16/5.7 | 160/5.4 |
| 47 | Protocadherin gamma subfamily C | 18087749 | 7 | 1.83 | 102.00/5.1 | 130/6.5 |
|  |  |  |  |  |  |  |
|  | **Cyclase:** |  |  |  |  |  |
| 48 | Adenylate cyclase type 8 | 2492893 | 7 | 1.98 | 141.56/6.5 | 155/7.2 |
|  |  |  |  |  |  |  |
|  | **Cytoskeletal/Structural:** |  |  |  |  |  |
| 49 | actin, gamma | 809561 | 25 | 1.37 | 41.43/5.6 | 42/5.2 |
| 50 | Actin-binding protein MIPP | 12642544 | 14 | 1.24 | 66.64/5.3 | 68/5.2 |
| 51 | Actin-regulatory protein CAP-G | 729023 | 22 | 1.80 | 39.51/6.7 | 43/7.2 |
| 52 | Capg protein | 18605629 | 19 | 1.75 | 39.04/6.5 | 46/7.1 |
| 53 | CAST1/ERC2 splicing variant-4 | 38231916 | 18 | 1.80 | 110.38/6.6 | 120/6.1 |
| 54 | Cortactin | 2498955 | 27 | 2.23 | 61.41/5.2 | 50/5.3 |
| 55 | Dystonin | 26332046 | 25 | 1.82 | 60.27/10.1 | 68/7.8 |
| 56 | Dystonin isoform 1a | 111154082 | 11 | 2.06 | 79.98/9.8 | 70/8.5 |
| 57 | Fascin | 2498358 | 17 | 1.95 | 55.13/6.2 | 55/6.8 |
| 58 | mKIAA0445 protein | 39104485 | 13 | 1.40 | 180.04/5.5 | 120/6.3 |
| 59 | Mtap7d1 protein | 18043435 | 24 | 1.74 | 60.05/10.2 | 80/9.5 |
| 60 | Myosin, heavy polypeptide 2, skeletal muscle, adult | 14250231 | 9 | 1.37 | 184.04/5.7 | 160/5.2 |
| 61 | Myosin-Va | 547968 | 13 | 1.75 | 217.23/9.1 | 40/8.0 |
| 62 | Nebulin | 1470066 | 16 | 1.46 | 158.66/9.3 | 140/9.5 |
| 63 | Rab6-interacting protein 2 isoform A | 13445784 | 17 | 1.84 | 112.03/6.1 | 160/7.6 |
|  |  |  |  |  |  |  |
|  | **DNA Binding protein:** |  |  |  |  |  |
| 64 | DNA replication licensing factor MCM5 | 1705525 | 16 | 1.65 | 83.13/9.3 | 110/8.0 |
| 65 | Histone H1 variant | 24111224 | 19 | 1.79 | 44.42/11.7 | 45/9.0 |
| 66 | Zinc finger, MYM-type 3 | 9790027 | 12 | 1.67 | 156.74/6.0 | 140/6.5 |
| 67 | Synaptonemal complex protein 1 | 1661003 | 17 | 1.37 | 116.57/5.7 | 105/5.2 |
| 68 | Ureb1 protein | 32484199 | 18 | 1.87 | 52.29/6.0 | 52/7.1 |
| 69 | Zinc finger and BTB domain containing 41 homolog | 27369918 | 10 | 2.12 | 78.76/8.7 | 90/7.9 |
| 70 | zinc finger protein 445 | 45593859 | 18 | 1.75 | 117.1/9.9 | 105/8.5 |
|  |  |  |  |  |  |  |
|  | **Enzyme: Dehydrogenase** |  |  |  |  |  |
| 71 | glyceraldehyde-3-phosphate dehydrogenase | 6679937 | 29 | 1.74 | 36.06/8.7 | 32/8.2 |
| 72 | hydroxysteroid (17-beta) dehydrogenase 4 | 1213008 | 17 | 1.81 | 80.02/9.0 | 82/8.0 |
| 73 | malate dehydrogenase | 387129 | 20 | 2.03 | 36.63/6.2 | 35/7.1 |
| 74 | Oxalosuccinate decarboxylase | 6647554 | 28 | 1.98 | 47.04/6.4 | 60/7.1 |
|  |  |  |  |  |  |  |
|  | **Enzyme: Isomerase** |  |  |  |  |  |
| 75 | Glucose regulated protein | 23958822 | 24 | 1.61 | 57.12/5.8 | 60/5.9 |
| 76 | Peptidyl-prolyl cis-trans isomerase A (PPIase) | 38090011 | 58 | 1.88 | 14.52/8.0 | 20/7.8 |
| 77 | Peptidylprolyl isomerase C | 19483859 | 32 | 2.42 | 19.96/6.2 | 19/5.8 |
| 78 | Protein disulfide-isomerase (EC 5.3.4.1) ERp61 precursor | 1083311 | 24 | 2.33 | 57.12/5.8 | 55/5.2 |
| 79 | Isopentenyl-diphosphate delta isomerase | 21703726 | 30 | 2.015 | 26.61/5.8 | 25/6.3 |
|  |  |  |  |  |  |  |
|  | **Enzyme: Mutase** |  |  |  |  |  |
| 80 | Pgam1 protein | 12805529 | 50 | 2.37 | 28.93/6.7 | 34/7.3 |
| 81 | phosphomannomutase 2 | 8393988 | 40 | 2.33 | 27.98/6.0 | 32/6.3 |
|  |  |  |  |  |  |  |
|  | **Enzyme: Peroxidase** |  |  |  |  |  |
| 82 | Peroxiredoxin-2 | 2499469 | 28 | 2.22 | 21.93/5.2 | 22/5.0 |
| 83 | Peroxiredoxin-6 | 30267702 | 41 | 2.04 | 24.97/5.7 | 27/5.7 |
| 84 | Proliferation-associated protein 1 | 6755100 | 18 | 1.46 | 44.02/6.4 | 45/7.0 |
|  |  |  |  |  |  |  |
|  | **Enzyme: Phospholipase** |  |  |  |  |  |
| 85 | Phospholipase C-alpha | 200397 | 24 | 1.79 | 57.12/5.8 | 50/6.8 |
| 86 | Phospholipase D1 (PLD 1) | 13124458 | 16 | 1.88 | 124.75/9.1 | 115/7.5 |
| 87 | phospholipase C, eta 1 | 34147246 | 11 | 1.64 | 184.86/7.0 | 160/9.2 |
|  |  |  |  |  |  |  |
|  | **Enzyme Reductase:** |  |  |  |  |  |
| 88 | L-xylulose reductase | 50400594 | 33 | 1.84 | 25.93/6.8 | 28/6.9 |
| 89 | Pyrroline-5-carboxylate reductase-like | 12833245 | 27 | 1.32 | 29.13/6.8 | 33/8.1 |
| 90 | Sepiapterin reductase (SPR) | 2498952 | 36 | 2.05 | 28.21/5.6 | 29/7.1 |
|  |  |  |  |  |  |  |
|  | **Enzyme: Synthase** |  |  |  |  |  |
| 91 | Spermidine synthase | 13542723 | 12 | 1.97 | 33.98/5.3 | 32/5.6 |
|  |  |  |  |  |  |  |
|  | **Extracellular matrix:** |  |  |  |  |  |
| 92 | Collagen alpha 2(IV) | 115350 | 7 | 1.38 | 168.57/9.2 | 105/5.2 |
|  |  |  |  |  |  |  |
|  | **G protein coupled receptor:** |  |  |  |  |  |
| 93 | Somatostatin receptor type 1 (SS1R) (SRIF-2) | 401125 | 19 | 1.61 | 43.44/9.3 | 48/7.9 |
|  |  |  |  |  |  |  |
|  | **GTPase:** |  |  |  |  |  |
| 94 | GTP binding protein 4 | 31560110 | 18 | 1.89 | 74.49/9.7 | 77/10.0 |
| 95 | RAB11a, member RAS oncogene family | 31980840 | 35 | 1.91 | 24.49/6.1 | 20/7.2 |
|  |  |  |  |  |  |  |
|  | **GTPase activating protein:** |  |  |  |  |  |
| 96 | Iqgap2 protein | 31807202 | 10 | 1.75 | 98.58/5.1 | 130/6.5 |
|  |  |  |  |  |  |  |
|  | **Guanine nucleotide exchange factor:** |  |  |  |  |  |
| 97 | Guanine nucleotide exchange factor 1 | 6755268 | 12 | 1.46 | 194.23/7.1 | 170/8.0 |
| 98 | Ngef protein | 24660296 | 17 | 1.02 | 82.69/5.7 | 82/6.6 |
| 99 | Vav2 oncogene | 6678555 | 15 | 1.49 | 101.21/6.4 | 110/7.2 |
| 100 | golgi-specific brefeldin A-resistance factor 1 | 26333343 | 15 | 1.73 | 83.90/5.6 | 88/5.4 |
|  |  |  |  |  |  |  |
|  | **Guanylate cyclase:** |  |  |  |  |  |
| 101 | Guanylate cyclase 2E | 1706241 | 15 | 2.05 | 121.73/8.7 | 125/9.0 |
|  |  |  |  |  |  |  |
|  | **Heat shock protein (Chaperone):** |  |  |  |  |  |
| 102 | Cyclophilin C | 1000036 | 32 | 2.15 | 19.96/6.2 | 18/6.8 |
| 103 | Heat shock protein 60 | [38091331](http://www.ncbi.nlm.nih.gov/htbin-post/Entrez/query?form=6&db=p&Dopt=g&uid=38091331) | 21 | 2.05 | 60.71/5.6 | 60/5.0 |
| 104 | Heat-shock protein beta-1 (HSP 27) | 17390597 | 47 | 1.67 | 23.05/6.1 | 26/6.5 |
| 105 | HSP27-related protein | 1083369 | 28 | 1.13 | 22.94/6.4 | 23/6.1 |
| 106 | MAMA/CyCAP precursor | 1363194 | 12 | 1.29 | 65.39/5.0 | 68/5.0 |
|  |  |  |  | 2.28 |  |  |
|  | **Hydratase:** |  |  |  |  |  |
| 107 | Enolase 3 | 6679651 | 19 | 1.68 | 47.35/6.7 | 45/6.5 |
|  |  |  |  |  |  |  |
|  | **Hydrolase:** |  |  |  |  |  |
| 108 | NG,NG-dimethylarginine dimethylaminohydrolase 2 | 13097101 | 22 | 1.85 | 25.52/5.7 | 23/6.4 |
|  |  |  |  |  |  |  |
|  | **Ion Channel:** |  |  |  |  |  |
| 109 | Gprin1 protein, GRIN1 | 34784265 | 16 | 1.65 | 96.11/8.5 | 90/7.9 |
| 110 | Potassium voltage gated channel, Shab-related subfamily, member 1 | 31560819 | 19 | 2.27 | 96.4 3/8.8 | 100/7.5 |
|  |  |  |  |  |  |  |
|  | **Metallopeptidase activity:** |  |  |  |  |  |
| 111 | Matrix metalloproteinase-12 | 461771 | 20 | 1.69 | 54.05/9.3 | 55/8.5 |
|  |  |  |  |  |  |  |
|  | **Motor protein:** |  |  |  |  |  |
| 112 | kinesin heavy chain | 52797 | 17 | 1.56 | 117.90/5.8 | 130/6.5 |
|  |  |  |  |  |  |  |
|  | **Oxidioreductase:** |  |  |  |  |  |
| 113 | Aldh2 protein | 13529509 | 14 | 1.96 | 57.03/7.7 | 52/7.9 |
|  | **Phosphoprotein:** |  |  |  |  |  |
| 114 | CASK-A | 3087816 | 22 | 2.19 | 75.94/6.0 | 85/7.0 |
|  |  |  |  |  |  |  |
|  | **Phosphotransferase:** |  |  |  |  |  |
| 115 | Citron, Citron-K kinase, Cit protein | 29881616 | 17 | 1.23 | 189.35/6.1 | 200/6.5 |
| 116 | [PTK2 protein tyrosine kinase 2 beta](http://www.ncbi.nlm.nih.gov/htbin-post/Entrez/query?form=6&db=p&Dopt=g&uid=27369678) | [27369678](http://www.ncbi.nlm.nih.gov/htbin-post/Entrez/query?form=6&db=p&Dopt=g&uid=27369678) | 12 | 1.48 | 112.37/5.7 | 95/8.2 |
| 117 | Rho-associated protein kinase 2 | 47605990 | 15 | 2.04 | 161.71/5.7 | 170/6.9 |
| 118 | Testicular protein | 735904 | 25 | 1.97 | 120.2/5.7 | 110/7.8 |
| 119 | UMP-CMP kinase (Cytidylate kinase) | 23821758 | 38 | 2.04 | 22.38/5.7 | 23/4.5 |
|  |  |  |  |  |  |  |
|  | **Protease Inhibitor:** |  |  |  |  |  |
| 120 | Spink5 protein/RIKEN cDNA 2310065D10 | 57282595 | 26 | 1.81 | 118.23/9.4 | 140/8.6 |
|  |  |  |  |  |  |  |
|  |  |  |  |  |  |  |
|  | **Protein binding:** |  |  |  |  |  |
| 121 | Fibrinogen B-beta-chain | 15593264 | 26 | 1.81 | 27.26/8.5 | 30/10.0 |
| 122 | Fibrinogen, B beta polypeptide | 21619364 | 14 | 1.15 | 55.42/6.7 | 55/7.5 |
|  |  |  |  |  |  |  |
|  | **Protein Phosphatase:** |  |  |  |  |  |
| 123 | RPTPmam4 isoform III | 13378310 | 14 | 2.17 | 164.81/6.4 | 145/7.0 |
| 124 | Protein tyrosine phosphatase, receptor type, R | 6755248 | 12 | 1.93 | 74.76/8.3 | 85/8.2 |
| 125 | Sac domain-containing inositol phosphatase 3 | 19527220 | 7 | 1.49 | 104.17/6.5 | 110/6.0 |
|  |  |  |  |  |  |  |
|  | **Reductase:** |  |  |  |  |  |
| 126 | Carbonyl reductase 3 | 20380344 | 21 | 1.66 | 31.33/6.2 | 42/6.2 |
|  |  |  |  |  |  |  |
|  | **Ribonuclease:** |  |  |  |  |  |
| 127 | serine/arginine repetitive matrix 2 | 28972153 | 13 | 1.33 | 51.47/11.9 | 65/9.5 |
|  |  |  |  |  |  |  |
|  | **RNA binding protein:** |  |  |  |  |  |
| 128 | KIAA1141 | 50510803 | 13 | 1.52 | 103.76/9.7 | 110/8.2 |
| 129 | Prpf8 protein | 21961512 | 10 | 1.54 | 140.13/8.9 | 130/8.5 |
| 130 | Rbm3 protein | 13879226 | 44 | 2.04 | 16.59/6.8 | 15/7.2 |
| 131 | Zinc finger protein 100 | 50510803 | 12 | 1.80 | 104.80/9.3 | 98/9.5 |
|  |  |  |  |  |  |  |
|  | **RNA helicase:** |  |  |  |  |  |
| 132 | DDX46 | 26330506 | 11 | 1.94 | 93.29/10.1 | 120/10.0 |
|  |  |  |  |  |  |  |
|  | **Transaldolase:** |  |  |  |  |  |
| 133 | transaldolase 1 | 33859640 | 26 | 2.02 | 37.54/6.6 | 40/7.5 |
|  |  |  |  |  |  |  |
|  | **Transcription factor:** |  |  |  |  |  |
| 134 | heterogeneous nuclear ribonucleoprotein M isoform a | 38074254 | 14 | 1.66 | 136.35/9.1 | 135/7.5 |
| 135 | Piwi/Argonaute family protein meIF2C4 | 22830893 | 22 | 1.91 | 98.13/9.6 | 101/8.3 |
|  |  |  |  |  |  |  |
|  | **Transcription regulatory protein:** |  |  |  |  |  |
| 136 | histone deacetylase 2 | 87162464 | 27 | 1.53 | 35.13/8.4 | 40/8.2 |
| 137 | KIAA1610 protein | 37360474 | 12 | 1.89 | 63.63/4.4 | 60/4.5 |
| 138 | MLL3-like protein | 14626492 | 26 | 2.21 | 78.94/9.6 | 80/9.5 |
| 139 | Period protein Per3 | 7513766 | 8 | 1.67 | 122.73/5.9 | 120/6.0 |
| 140 | SNF2 histone linker PHD RING helicase isoform A | 32480768 | 10 | 1.14 | 187.61/7.6 | 80/10.0 |
|  |  |  |  |  |  |  |
|  | **Transferase:** |  |  |  |  |  |
| 141 | 2'-5'oligoadenylate synthetase 1F | 21553117 | 30 | 1.85 | 42.88/7.1 | 50/8.0 |
|  |  |  |  |  |  |  |
|  | **Translation Regulatory Protein:** |  |  |  |  |  |
| 142 | TUFM protein, Tu translation elongation factor, mitochondrial | 38173913 | 24 | 2.16 | 47.74/7.9 | 62/7.8 |
|  |  |  |  |  |  |  |
|  | **Transport/Cargo:** |  |  |  |  |  |
| 143 | ARF binding protein 2 | 38614377 | 10 | 1.67 | 66.49/6.9 | 50/7.0 |
| 144 | [ATP synthase, H+ transporting mitochondrial F1 complex, beta subunit](http://www.ncbi.nlm.nih.gov/htbin-post/Entrez/query?form=6&db=p&Dopt=g&uid=28302366) | [28302366](http://www.ncbi.nlm.nih.gov/htbin-post/Entrez/query?form=6&db=p&Dopt=g&uid=28302366) | 27 | 2.35 | 56.28/5.2 | 56/5.2 |
| 145 | Atp5b protein | 23272966 | 22 | 2.33 | 56.55/5.2 | 55/5.0 |
| 146 | Sorting nexin 19 | 38649129 | 12 | 1.40 | 111.16/5.0 | 110/5.8 |
|  |  |  |  |  |  |  |
|  | **Ubiquitin proteasome system protein:** |  |  |  |  |  |
| 147 | LaXp180 protein | 20819700 | 15 | 1.48 | 184.40/5.3 | 200/6.8 |
| 148 | Valosin-containing protein p97/p47 complex-interacting protein p135 | 42559967 | 13 | 1.52 | 135.74/6.7 | 160/8.5 |
|  |  |  |  |  |  |  |
|  | **Unclassified/Unknown function:** |  |  |  |  |  |
| 149 | calmodulin regulated spectrin-associated protein 1 | 26330506 | 7 | 1.31 | 133.57/6.8 | 160/7.5 |
| 150 | Cobll1 protein | 44890604 | 13 | 1.59 | 111.50/6.6 | 110/6.8 |
| 151 | Disheveled associated activator of morphogenesis 1 | 34098511 | 12 | 1.66 | 123.1/7.1 | 105/7.5 |
| 152 | Epidermal growth factor receptor kinase substrate 8 | 2833214 | 13 | 1.48 | 92.06/7.2 | 150/8.5 |
| 153 | Gene model 1568 | 44890499 | 15 | 1.78 | 80.37/11.1 | 92/10.0 |
| 154 | Grp94 neighboring nucleotidase variant 3 | 50727145 | 5 | 1.61 | 152.52/9.3 | 150/7.2 |
| 155 | Guanylate binding protein 7 | 134031973 | 22 | 1.80 | 72.66/6.0 | 95/6.8 |
| 156 | High density lipoprotein binding protein | 23273831 | 18 | 1.73 | 142.30/6.4 | 90/7.5 |
| 157 | hypothetical protein LOC66625 isoform 1 | 28495127 | 12 | 1.91 | 93.29/10.1 | 90/10.0 |
| 158 | Leucine-rich repeats and WD repeat domain containing 1 | 166851848 | 16 | 1.69 | 72.77/8.8 | 68/10.0 |
| 159 | low density lipoprotein-related protein 12 | 38074578 | 7 | 1.15 | 133.57/6.8 | 110/6.5 |
| 160 | KIAA0635 protein | 50510561 | 18 | 1.38 | 132.18/6.0 | 158/5.9 |
| 161 | Ncapg2 | 38489966 | 11 | 1.65 | 98.32/8.7 | 100/8.5 |
| 162 | Multidrug resistance-associated protein 7A and 7B | 20271160 | 9 | 1.22 | 161.28/7.1 | 160/8.9 |
| 163 | Olfactory receptor 18 | 38089777 | 17 | 1.89 | 108.67/9.5 | 125/8.6 |
| 164 | PDZ domain-containing RING finger protein 4 | 149266793 | 14 | 2.06 | 57.68/6.2 | 55/7.0 |
| 165 | pORF2 | 16508047 | 19 | 1.99 | 150.56/9.8 | 80/9.0 |
| 166 | Proline arginine rich coiled coil 1 | 31542192 | 14 | 2.23 | 93.67/10.3 | 110/7.9 |
| 167 | RIKEN cDNA 1700021E15 | 38082287 | 20 | 1.94 | 52.16/5.7 | 53/6.5 |
| 168 | RIKEN cDNA 4631416I11 | 38079496 | 16 | 1.81 | 124.74/5.0 | 125/5.1 |
| 169 | RIKEN cDNA C820005L12 | 27370214 | 12 | 1.66 | 97.22/5.1 | 100/5.0 |
| 170 | SAPS domain family, member 2 isoform 1 | 25453071 | 7 | 1.59 | 101.87/4.7 | 100/5.0 |
| 171 | Syntaxin binding protein 5-like | 27369591 | 9 | 1.76 | 113.05/6.7 | 120/6.5 |
| 172 | Thrombospondin type-1 domain-containing protein 7A precursor | 50510729 | 6 | 1.93 | 191.17/7.2 | 130/7.5 |
| 173 | Tmcc1 protein | 20987344 | 6 | 1.94 | 73.47/6.1 | 65/6.3 |
| 174 | TPA: testase-7 | 33186788 | 11 | 1.80 | 83.34/6.8 | 100/6.7 |
| 175 | Zinc finger, DHHC domain containing 13 | 27229145 | 10 | 1.94 | 71.96/9.1 | 75/6.0 |
| 176 | Zmym2 protein | 28175571 | 13 | 1.83 | 116.45/6.2 | 125/6.5 |

Note 1: Protein classification is according to the Human Protein Reference Database ([http://www.hprd.org](http://www.hprd.org/)).

Note 2: SC%: Percentage of sequence coverage.

Note 3: Spot Number refers to the location of the individual proteins on 2D gels as shown in Figures 3-6. When a protein was identified in more than one Batch IEX fraction, it is marked multiple gels.

Note 4: Supplemental Table 2 contains all MALDI TOF data generated for each protein.

Note 5: Only proteins with probability score of 1.0e+000 were included in the table.
