# Supplementary material for: A fractionation method to identify qauntitative changes in protein expression mediated by IGF-1 on the proteome of murine C2C12 myoblasts
Source: Proteome Sci. 2009 Aug 11;7:28. doi: 10.1186/1477-5956-7-28 (PMC2732595; doi:10.1186/1477-5956-7-28)
Supplement: Additional file 7 — MALDI-TOF data from each protein identified in these studies. The data provided represents all MALDI-TOF information from each protein identified in these studies. [file 1477-5956-7-28-S7.pdf]

Protein Name	Measured Mass (Da)	Calculated Mass (Da)	Error	Peptide Start	Peptide End	Missed cleavage	Sequence
2'-5'oligoadenylate synthetase 1F	1103.609	1103.597	0.012	287	295	1	TKVSEYLHK
2'-5'oligoadenylate synthetase 1F	1119.63	1119.592	0.038	97	105	1	GEFIEEIRK
2'-5'oligoadenylate synthetase 1F	1513.743	1513.757	-0.014	46	58	1	CFQGATHPVRVSR
2'-5'oligoadenylate synthetase 1F	1525.733	1525.861	-0.128	313	327	0	NVAGTNLLGWGLLAK
2'-5'oligoadenylate synthetase 1F	1531.751	1531.766	-0.016	162	173	1	NNKYAELYLYNK
2'-5'oligoadenylate synthetase 1F	1541.754	1541.765	-0.011	106	117	0	HLCQLQDEKPFK
2'-5'oligoadenylate synthetase 1F	1552.741	1552.771	-0.03	276	286	1	IYWTVYYDFRK
2'-5'oligoadenylate synthetase 1F	1588.753	1588.827	-0.074	174	186	0	IYAQLIHECTTLK
2'-5'oligoadenylate synthetase 1F	1593.669	1593.753	-0.084	19	32	0	DHLLPDSSFHAEAR
6-phosphogluconolactonase	1182.625	1182.512	0.113	73	81	0	WTLGFCDER
6-phosphogluconolactonase	1369.823	1369.76	0.063	246	257	1	LLSVPFEKHSTL
6-phosphogluconolactonase	1481.839	1481.762	0.077	57	72	0	DLPAAAAPAGPASFAR
6-phosphogluconolactonase	1579.83	1579.745	0.085	232	245	0	TGALCWFLDEAAAR
6-phosphogluconolactonase	1600.948	1600.893	0.055	171	185	0	IVAPISDSPKPPPQR
6-phosphogluconolactonase	1766.896	1766.862	0.034	82	96	0	LVPFDHAESTYGLYR
6-phosphogluconolactonase	1849.05	1849.03	0.02	215	231	1	ILEDKEGTLPAALVQPR
78 kDa glucose-regulated protein precursor (GRP 78) (Heat shock 70 kDa protein 5)	1208.618	1208.559	0.059	593	602	1	***ETMEKAVEEK***
78 kDa glucose-regulated protein precursor (GRP 78) (Heat shock 70 kDa protein 5)	1312.69	1312.612	0.078	328	337	0	FEELNMDLFR
78 kDa glucose-regulated protein precursor (GRP 78) (Heat shock 70 kDa protein 5)	1658.808	1658.887	-0.079	199	214	0	IINEPTAAAIAYGLDK
78 kDa glucose-regulated protein precursor (GRP 78) (Heat shock 70 kDa protein 5)	1810.886	1810.92	-0.034	140	155	1	***TFAPEEISAMVLTKMK***
78 kDa glucose-regulated protein precursor (GRP 78) (Heat shock 70 kDa protein 5)	1835.926	1835.926	0	449	465	0	SQIFSTASDNQPTVTIK
78 kDa glucose-regulated protein precursor (GRP 78) (Heat shock 70 kDa protein 5)	1932.979	1933.005	-0.026	476	493	0	DNHLLGTFDLTGIPPAPR
78 kDa glucose-regulated protein precursor (GRP 78) (Heat shock 70 kDa protein 5)	1963.973	1964.02	-0.047	448	465	1	KSQIFSTASDNQPTVTIK
78 kDa glucose-regulated protein precursor (GRP 78) (Heat shock 70 kDa protein 5)	1998.005	1998.078	-0.073	494	511	0	GVPQIEVTFEIDVNGILR
Actin regulatory protein CAP-G	1374.869	1374.725	0.144	311	323	0	QAALQVADGFISR
Actin regulatory protein CAP-G	1484.87	1484.805	0.064	192	204	2	ARDLALAIRDSER
Actin regulatory protein CAP-G	1570.894	1570.842	0.052	194	207	2	DLALAIRDSERQGK
Actin regulatory protein CAP-G	1600.877	1600.82	0.057	326	339	0	YSPNTQVEILPQGR
Actin regulatory protein CAP-G	1839.881	1839.926	-0.045	113	128	2	GLKYYREGGVESAFHK
Actin regulatory protein CAP-G	1887.905	1887.962	-0.057	324	339	1	MRYSPNTQVEILPQGR
Actin regulatory protein CAP-G	1903.89	1903.956	-0.066	324	339	1	***MRYSPNTQVEILPQGR***
Actin regulatory protein CAP-G	1917.767	1917.856	-0.089	97	112	0	EVQGNESDLFMSYFPR
Actin regulatory protein CAP-G	1933.724	1933.851	-0.127	97	112	0	***EVQGNESDLFMSYFPR***
actin, gamma	975.496	975.441	0.055	12	21	0	AGFAGDDAPR
actin, gamma	1208.626	1208.552	0.074	1	11	0	***LVIDNGSGMCK***
actin, gamma	1638.865	1638.828	0.037	171	184	1	***LDLAGRDLTDYLMK***
actin, gamma	1789.923	1789.884	0.039	232	247	0	SYELPDGQVITIGNER
actin, gamma	1953.105	1953.056	0.049	89	106	0	VAPEEHPVLLTEAPLNPK
actin, gamma	2358.191	2358.151	0.04	284	305	1	***KDLYANTVLSGGTTMYPGIADR***
Actin-binding protein IPP	956.51	956.544	-0.034	228	235	0	FPLLPSQR
Actin-binding protein IPP	984.485	984.458	0.027	1	8	1	MSKEEYAK
Actin-binding protein IPP	986.498	986.383	0.115	277	283	0	EYCEVCK
Actin-binding protein IPP	986.498	986.383	0.115	258	264	0	EYCEVCK
Actin-binding protein IPP	1219.567	1219.601	-0.034	417	427	0	WEVVGSMAVSR
Actin-binding protein IPP	1312.665	1312.67	-0.005	465	475	1	***WSPLPPMGTRR***
Actin-binding protein IPP	1312.665	1312.67	-0.005	464	474	1	***RWSPLPPMGTR***
Actin-binding protein IPP	1339.697	1339.677	0.02	454	464	1	SFEVYDPLSKR
Actin-binding protein IPP	1426.734	1426.677	0.057	369	380	0	QWTTVASMNHPR
Actin-binding protein IPP	1835.925	1835.926	-0.001	334	352	0	CGLGVAVVGGMVYAIGGEK
Actin-binding protein IPP	1852.912	1852.942	-0.03	250	264	1	VALQTLLKEYCEVCK
Adenylate cyclase type 8	985.516	985.57	-0.055	36	43	0	LLWQTAVR

Protein Name	Measured Mass (Da)	Calculated Mass (Da)	Error	Peptide Start	Peptide End	Missed cleavage	Sequence
Adenylate cyclase type 8	1428.803	1428.756	0.046	1208	1220	0	QLLNENSNSGIIK
Adenylate cyclase type 8	1445.848	1445.779	0.069	179	191	0	SEVVMNVLDVLTK
Adenylate cyclase type 8	1655.809	1655.807	0.002	1033	1048	0	***TIGSTYMAVSGLSPEK***
Adenylate cyclase type 8	1768.896	1768.891	0.005	587	601	0	QPEESLLCLPEDIVK
Adenylate cyclase type 8	1973.969	1973.994	-0.025	1008	1024	0	LLNEIIADFDELLGEDR
aldehyde dehydrogenase 2	989.494	989.493	0.001	349	357	0	VVGNPFDSR
aldehyde dehydrogenase 2	1131.573	1131.582	-0.009	87	96	0	AAFQLGSPWR
aldehyde dehydrogenase 2	1469.77	1469.755	0.015	397	409	0	GYFIQPTVFGDVK
aldehyde dehydrogenase 2	1530.768	1530.735	0.033	162	174	0	TIPIDGDFFSYTR
aldehyde dehydrogenase 2	1598.801	1598.782	0.019	495	508	0	ELGEYGLQAYTEVK
aldehyde dehydrogenase 2	1773.817	1773.82	-0.003	327	340	0	TFVQENVYDEFVER
Annexin A3	942.484	942.444	0.04	211	217	0	LTFDEYR
Annexin A3	1071.601	1071.628	-0.027	40	48	0	TLINILTER
Annexin A3	1084.608	1084.635	-0.027	155	164	1	ALLTLADGRR
Annexin A3	1491.793	1491.76	0.033	127	138	1	EISQAYYTVYKK
Annexin A3	1613.843	1613.841	0.002	280	292	1	SEIDLLDIRHEFK
Annexin A3	1703.82	1703.857	-0.037	105	120	0	GTGTDEDALIEILTTR
Annexin A3	1712.788	1712.784	0.004	138	153	1	KSLGDDISSETSGDFR
Annexin A3	1712.788	1712.784	0.004	139	154	1	SLGDDISSETSGDFRK
Annexin A3	1771.93	1771.967	-0.037	33	48	1	GLGTDEKTLINILTER
Annexin A3	2022	2022.005	-0.005	10	28	1	GTIKDYPGFSPSVDAEAIR
Annexin A3	2066.027	2066.019	0.008	102	120	1	***SMKGTGTDEDALIEILTTR***
Annexin A3	2181.965	2181.959	0.005	294	312	0	HYGYSLYSAIQSDTSGDYR
ARF binding protein 2	1005.527	1005.461	0.066	222	229	1	EEQEKSEK
ARF binding protein 2	1130.679	1130.648	0.031	175	184	0	ILPPPSPWPK
ARF binding protein 2	1136.682	1136.601	0.081	151	159	1	IRDAYQMLK
ARF binding protein 2	1312.786	1312.663	0.123	246	255	1	***VLREMLSMYR***
ARF binding protein 2	1792.877	1792.793	0.084	36	51	0	***ATDPSMAEQDWSAIQK***
ARF binding protein 2	1835.868	1835.904	-0.036	576	592	0	LTFNQGGQPFSEVGEVK
ATP synthase, H+ transporting mitochondrial F1 complex, beta subunit	1087.597	1087.627	-0.03	189	198	0	VVDLLAPYAK
ATP synthase, H+ transporting mitochondrial F1 complex, beta subunit	1277.668	1277.628	0.04	110	121	0	***TIAMDGTEGLVR***
ATP synthase, H+ transporting mitochondrial F1 complex, beta subunit	1405.73	1405.673	0.057	226	239	0	AHGGYSVFAGVGER
ATP synthase, H+ transporting mitochondrial F1 complex, beta subunit	1590.831	1590.803	0.028	110	124	1	***TIAMDGTEGLVRGQK***
ATP synthase, H+ transporting mitochondrial F1 complex, beta subunit	1600.828	1600.802	0.026	265	279	0	VALVYGQMNEPPGAR
ATP synthase, H+ transporting mitochondrial F1 complex, beta subunit	1616.81	1616.797	0.013	265	279	0	***VALVYGQMNEPPGAR***
ATP synthase, H+ transporting mitochondrial F1 complex, beta subunit	1649.915	1649.909	0.006	95	109	0	LVLEVAQHLGESTVR
ATP synthase, H+ transporting mitochondrial F1 complex, beta subunit	1795.912	1795.949	-0.037	144	159	1	***IMNVIGEPIDERGPIK***
ATP synthase, H+ transporting mitochondrial F1 complex, beta subunit	1857.882	1857.867	0.015	407	422	0	***IMDPNIVGNEHYDVAR***
ATP synthase, H+ transporting mitochondrial F1 complex, beta subunit	1918.082	1918.088	-0.006	125	143	1	VLDSGAPIKIPVGPETLGR
ATP synthase, H+ transporting mitochondrial F1 complex, beta subunit	1987.029	1987.025	0.004	388	406	0	AIAELGIYPAVDPLDSTSR
ATP synthase, H+ transporting mitochondrial F1 complex, beta subunit	2008.063	2008.069	-0.006	92	109	1	DSRLVLEVAQHLGESTVR
Atp5b protein	1087.597	1087.627	-0.03	193	202	0	VVDLLAPYAK
Atp5b protein	1277.668	1277.628	0.04	114	125	0	***TIAMDGTEGLVR***
Atp5b protein	1405.73	1405.673	0.057	230	243	0	AHGGYSVFAGVGER
Atp5b protein	1590.831	1590.803	0.028	114	128	1	***TIAMDGTEGLVRGQK***
Atp5b protein	1600.828	1600.802	0.026	269	283	0	VALVYGQMNEPPGAR
Atp5b protein	1616.81	1616.797	0.013	269	283	0	***VALVYGQMNEPPGAR***
Atp5b protein	1649.915	1649.909	0.006	99	113	0	***LVLEVAQHLGESTVR***
Atp5b protein	1795.912	1795.949	-0.037	148	163	1	IMNVIGEPIDERGPIK
Atp5b protein	1857.882	1857.867	0.015	411	426	0	***IMDPNIVGNEHYDVAR***
Atp5b protein	1918.082	1918.088	-0.006	129	147	1	VLDSGAPIKIPVGPETLGR

Protein Name	Measured Mass (Da)	Calculated Mass (Da)	Error	Peptide Start	Peptide End	Missed cleavage	Sequence
Atp5b protein	1987.029	1987.025	0.004	392	410	0	AIAELGIYPAVDPLDSTSR
Atp5b protein	2008.063	2008.069	-0.006	96	113	1	DSRLVLEVAQHLGESTVR
calmodulin regulated spectrin-associated protein 1	940.604	940.468	0.136	778	785	0	TFVLSSCK
calmodulin regulated spectrin-associated protein 1	983.633	983.528	0.105	659	668	0	SPTVPTPGTK
calmodulin regulated spectrin-associated protein 1	1295.816	1295.719	0.097	177	189	0	TSPQAPGLVASIR
calmodulin regulated spectrin-associated protein 1	1301.773	1301.707	0.066	830	841	0	ASLIEVDLSDLK
calmodulin regulated spectrin-associated protein 1	1325.836	1325.755	0.081	1023	1034	0	VESLEALPILSR
calmodulin regulated spectrin-associated protein 1	1411.868	1411.73	0.138	729	741	0	TPTPSVETLPQSR
calmodulin regulated spectrin-associated protein 1	1552.954	1552.82	0.134	948	961	0	QQQALEEQGLGKPK
CapG protein	972.492	972.535	-0.043	183	190	1	SNILERNK
CapG protein	1374.746	1374.725	0.021	308	320	0	QAALQVADGFISR
CapG protein	1378.719	1378.662	0.057	116	127	1	YREGGVESAFHK
CapG protein	1600.829	1600.82	0.009	323	336	0	YSPNTQVEILPQGR
CapG protein	1887.84	1887.962	-0.122	321	336	1	MRYSPNTQVEILPQGR
CapG protein	1903.921	1903.956	-0.035	321	336	1	***MRYSPNTQVEILPQGR***
CapG protein	1933.836	1933.851	-0.015	97	112	0	***EVQGNESDLFMSYFPR***
Carbonyl reductase 3	1466.826	1466.783	0.043	45	58	0	AAVQQLQAEGLSPR
Carbonyl reductase 3	1534.896	1534.773	0.123	29	42	1	FSGDVVLTARDEAR
Carbonyl reductase 3	1582.847	1582.773	0.074	59	71	0	FHQLDIDDPQSIR
Carbonyl reductase 3	1662.913	1662.868	0.045	28	42	2	KFSGDVVLTARDEAR
Carbonyl reductase 3	1679.917	1679.906	0.011	43	58	1	GRAAVQQLQAEGLSPR
Carbonyl reductase 3	1779.928	1779.911	0.016	120	134	0	***NVCTELLPIMKPHGR***
CASK interacting protein 1	1520.863	1520.731	0.132	904	920	0	ASDLAGSVDTGSAGSVK
CASK interacting protein 1	1533.889	1533.781	0.108	921	936	0	***SIAAMLELSSIGGGGR***
CASK interacting protein 1	1541.821	1541.767	0.054	69	81	0	GMRPLHYAAWQGR
CASK interacting protein 1	1619.876	1619.826	0.05	292	306	0	AGDIITVLEQHPDGR
CASK interacting protein 1	1655.896	1655.774	0.122	1308	1322	0	QEDGQGPRPSSIEEK
CASK interacting protein 1	1676.891	1676.832	0.059	903	920	1	RASDLAGSVDTGSAGSVK
CASK interacting protein 1	1769.908	1769.93	-0.022	814	830	0	DELLVPAAAGPYATVQR
CASK interacting protein 1	1831.967	1831.928	0.039	367	385	1	KPFAGGDRSGSLSNVAGGR
CASK interacting protein 1	1831.967	1831.865	0.102	644	660	0	SQEYLLDEGMAPGTPPK
CASK interacting protein 1	1847.993	1847.86	0.133	644	660	0	***SQEYLLDEGMAPGTPPK***
CASK interacting protein 1	1898.995	1898.932	0.063	66	81	1	DNKGMRPLHYAAWQGR
CASK interacting protein 1	1926.037	1926.031	0.005	814	831	1	DELLVPAAAGPYATVQRR
CASK interacting protein 1	1940.052	1940.022	0.03	464	480	0	ANLAVWLSMIGLAQYYK
CASK interacting protein 1	1940.052	1940.022	0.03	464	480	0	ANLAVWLSMIGLAQYYK
CASK-A	1705.832	1705.867	-0.035	28	41	2	RCINRETGQQFAVK
CASK-A	1869.911	1869.979	-0.068	538	554	0	EINGISVANQTVEQLQK
CASK-A	1960.95	1961.028	-0.078	490	506	1	LVQFQKNTDEPMGITLK
CASK-A	1960.95	1960.957	-0.007	235	250	2	YKMNPRQWSHISESAK
CASK-A	1998.962	1999.04	-0.078	630	647	1	ITEQKEVPPTSSALLACR
CASK-A	2005.958	2006.056	-0.098	42	60	1	IVDVAKFTSSPGLSTEDLK
CASK-A	2162.029	2162.068	-0.039	233	250	3	***GKYKMNPRQWSHISESAK***
CASK-A	2183.102	2183.17	-0.068	555	572	3	MLREMRGSITFKIVPSYR
CASK-A	2272.105	2272.131	-0.026	518	537	1	***IMHGGMIHRQGTLHVGDEIR***
CASK-A	2288.107	2288.125	-0.019	518	537	1	***IMHGGMIHRQGTLHVGDEIR***
CASK-A	2343.105	2343.125	-0.021	346	366	0	AVSQVLDSLEEIHALTDCSEK
CAST1/ERC2 splicing variant-4	1125.671	1125.596	0.075	747	755	1	IAELERHMK
CAST1/ERC2 splicing variant-4	1132.653	1132.59	0.062	199	208	1	EEAARMSVLK
CAST1/ERC2 splicing variant-4	1147.673	1147.579	0.094	843	851	0	***QLEEILEMK***
CAST1/ERC2 splicing variant-4	1147.673	1147.59	0.083	282	290	1	KTLEEMELR

Protein Name	Measured Mass (Da)	Calculated Mass (Da)	Error	Peptide Start	Peptide End	Missed cleavage	Sequence
CAST1/ERC2 splicing variant-4	1149.657	1149.602	0.054	612	620	1	LEEIESFRK
CAST1/ERC2 splicing variant-4	1152.679	1152.625	0.054	363	372	1	RSQLQPEPAK
CAST1/ERC2 splicing variant-4	1156.68	1156.656	0.024	756	765	2	DQNKKVANLK
CAST1/ERC2 splicing variant-4	1174.679	1174.619	0.06	819	829	0	LASTQQSLAEK
CAST1/ERC2 splicing variant-4	1191.673	1191.616	0.057	878	887	1	***KTQEEVMALK***
CAST1/ERC2 splicing variant-4	1219.662	1219.622	0.04	879	888	1	***TQEEVMALKR***
CAST1/ERC2 splicing variant-4	1219.662	1219.601	0.061	697	706	1	MNPEFADRLK
CAST1/ERC2 splicing variant-4	1253.723	1253.691	0.032	746	755	2	KIAELERHMK
CAST1/ERC2 splicing variant-4	1284.756	1284.678	0.078	156	165	1	ELQRENDLLR
CAST1/ERC2 splicing variant-4	1287.747	1287.689	0.058	808	818	2	TRQELDATKAR
CAST1/ERC2 splicing variant-4	1300.775	1300.709	0.066	291	301	1	IETQKQTLNAR
CAST1/ERC2 splicing variant-4	1319.73	1319.711	0.019	877	887	2	***KKTQEEVMALK***
CAST1/ERC2 splicing variant-4	1331.757	1331.692	0.064	510	520	1	TKQLQDLTEEK
CAST1/ERC2 splicing variant-4	1331.757	1331.722	0.034	878	888	2	KTQEEVMALKR
CAST1/ERC2 splicing variant-4	1375.789	1375.737	0.052	375	386	1	ALQTVIEMKDTK
CAST1/ERC2 splicing variant-4	1381.779	1381.767	0.012	363	374	2	RSQLQPEPAKTK
CAST1/ERC2 splicing variant-4	1398.796	1398.844	-0.048	664	675	2	LKSLEIAIEQKK
CAST1/ERC2 splicing variant-4	1406.79	1406.729	0.061	416	426	1	EEEIKQIEVYK
CAST1/ERC2 splicing variant-4	1429.749	1429.813	-0.064	434	445	2	TKIDQLKQELSK
Cep290 protein	983.629	983.503	0.126	730	737	1	QHSKEEVK
Cep290 protein	1014.622	1014.498	0.124	585	594	0	APQLEGADSK
Cep290 protein	1032.612	1032.581	0.031	483	492	1	KASGILTSEK
Cep290 protein	1156.653	1156.717	-0.064	60	69	2	LKSQLALKEK
Cep290 protein	1184.699	1184.712	-0.013	502	511	2	NLKAELEKLK
Cep290 protein	1184.699	1184.673	0.026	444	455	2	LGQVRGAGRSGK
Cep290 protein	1209.74	1209.719	0.021	415	424	2	DLPRLKNQVK
Cep290 protein	1270.788	1270.699	0.089	474	483	2	VQRENEQLKK
Cep290 protein	1302.767	1302.702	0.065	542	552	2	ELKKEIEASEK
Cep290 protein	1343.857	1343.788	0.069	183	194	2	QIKRLSSGLQSK
Cep290 protein	1369.861	1369.756	0.105	555	566	1	IAKNNLELVNDK
Cep290 protein	1427.85	1427.834	0.016	201	212	2	QSLIDELQKKVK
Cep290 protein	1446.85	1446.713	0.137	493	504	1	MATIEEENRNLK
Cep290 protein	1471.924	1471.831	0.093	456	468	1	TIPELEKTIGLMK
Cep290 protein	1515.906	1515.814	0.092	394	406	1	ENLKLSSENIELK
Cep290 protein	1554.966	1554.843	0.123	598	610	2	***SIVVSRVYETKMK***
Cep290 protein	1595.926	1595.851	0.075	725	737	2	LELEKQHSKEEVK
Cep290 protein	1612.013	1611.894	0.119	470	482	2	LVEKVQRENEQLK
Cep290 protein	1630.958	1630.882	0.076	48	61	2	***APTTTMRNLVDRLK***
Cep290 protein	1656.021	1655.908	0.112	119	132	1	SQIEDLNENLLKLK
Cep290 protein	1748.997	1748.919	0.078	642	655	1	KYTEDLEQQIEILK
Cep290 protein	1782.959	1782.906	0.052	604	618	2	VYETKMKELESDIAK
Cep290 protein	1784.972	1784.951	0.021	116	130	1	ELKSQIEDLNENLLK
Cep290 protein	1914.139	1914.043	0.096	13	29	2	VKAEVEDLRHALAQAHK
Cep290 protein	2162.019	2162.155	-0.136	101	118	2	EANLNVQQVVERHTRELK
Cep290 protein	2272.139	2272.147	-0.008	558	577	2	NNLELVNDKMAAQLEETGKR
Cep290 protein	2288.128	2288.142	-0.014	558	577	2	***NNLELVNDKMAAQLEETGKR***
Cingulin	922.573	922.487	0.086	42	50	0	ASTYGVAVR
Cingulin	958.568	958.544	0.024	482	489	1	ERELTALK
Cingulin	986.549	986.55	-0.001	1009	1016	1	ISLERQNK
Cingulin	1055.598	1055.608	-0.01	394	401	1	LQELLERR
Cingulin	1487.765	1487.746	0.019	842	854	1	LNKELEQIGDDSK

Protein Name	Measured Mass (Da)	Calculated Mass (Da)	Error	Peptide Start	Peptide End	Missed cleavage	Sequence
Cingulin	1593.813	1593.822	-0.009	1082	1094	1	QHVNDQKDQLTLR
Cingulin	1593.813	1593.759	0.053	693	706	1	***VASETEAMMLGQRR***
Cingulin	1620.749	1620.777	-0.028	403	416	1	***GEVQQSSKELQNMK***
Cingulin	1620.749	1620.795	-0.047	691	705	1	AKVASETEAMMLGQR
Cingulin	1628.799	1628.774	0.024	631	644	0	ELQAEQQNQEVTGR
Cingulin	1666.76	1666.786	-0.026	16	30	0	***FITEPEGATEMGTLR***
Cingulin	1787.823	1787.774	0.049	520	535	0	***SMQDATQDHAALEAER***
Cingulin	1822.828	1822.887	-0.059	16	31	1	***FITEPEGATEMGTLRR***
Cingulin	1896.861	1896.899	-0.038	129	146	0	***SQSQASLTGLAFMSPSNR***
Cingulin	2023.922	2024.016	-0.094	304	322	1	ATIYGILREGSSESEASVR
Cingulin	2043.963	2043.927	0.036	520	537	1	***SMQDATQDHAALEAERQK***
citron	953.478	953.529	-0.051	239	246	1	HSLENKVK
citron	972.454	972.523	-0.07	230	237	1	KLVEAEER
citron	995.493	995.441	0.052	348	356	0	***AMINAMDSK***
citron	1000.471	1000.53	-0.059	231	238	1	LVEAEERR
citron	1080.525	1080.513	0.012	638	645	1	SQFECRVR
citron	1136.584	1136.495	0.089	323	331	0	ESLENMMQR
citron	1199.579	1199.59	-0.011	710	719	1	KHAMLEMNAR
citron	1219.544	1219.586	-0.042	996	1006	1	***DKMNSPGLQSK***
citron	1226.611	1226.577	0.034	443	452	0	EVSLEHEEQK
citron	1278.65	1278.729	-0.079	670	680	1	QVVELAVKEHK
citron	1295.686	1295.599	0.087	909	919	1	KESSTPEEFSR
citron	1312.651	1312.734	-0.083	168	178	1	LEKINAEQQLK
citron	1585.778	1585.795	-0.018	865	879	1	KATDHPHPSTPATAR
citron	1604.773	1604.782	-0.009	387	399	1	***AQEEMISELRQQK***
citron	1835.858	1835.886	-0.028	107	121	1	***LMMNQLEEDLVSARR***
citron	1892.933	1892.878	0.055	260	275	1	***DDIQTKSEQIQQMADK***
Cobll1 protein	1098.619	1098.603	0.016	909	917	1	EPTIKEVQR
Cobll1 protein	1139.652	1139.727	-0.075	137	146	1	VILKPKSLDK
Cobll1 protein	1587.761	1587.832	-0.071	123	136	0	FKPNTPIGMLDVEK
Cobll1 protein	1668.728	1668.725	0.003	325	339	1	ASCVERSTSVDDTDK
Cobll1 protein	1672.737	1672.78	-0.043	669	682	1	EFRSQGTSTYVQDR
Cobll1 protein	1745.728	1745.867	-0.139	433	449	1	EELSEASKDPAGSISVK
Cobll1 protein	1770.771	1770.754	0.016	753	767	1	***SLEMAKDWESEAMGR***
Cobll1 protein	1835.794	1835.893	-0.099	450	466	1	***SPDIASASTDMRITVEK***
Cobll1 protein	1946.792	1946.906	-0.114	467	486	1	DPDSALGISDGETSPSSKGK
cofilin 1	1339.791	1339.77	0.02	153	166	0	LGGSAVISLEGKPL
cofilin 1	1680.794	1680.788	0.007	82	95	1	YALYDATYETKESK
cofilin 1	1887.983	1887.976	0.007	97	112	0	EDLVFIFWAPENAPLK
cofilin 1	1998.982	1998.975	0.008	1	19	1	***MASGVAVSDGVIKVFNDMK***
collagen alpha 2 (IV)	1360.723	1360.623	0.1	723	735	0	***EGFPGPPGFMGPR***
collagen alpha 2 (IV)	1486.789	1486.727	0.062	318	332	1	GSRGLDGFQGPSGPR
collagen alpha 2 (IV)	1486.789	1486.701	0.088	903	917	1	***GMAGMPGIPGQKGDR***
collagen alpha 2 (IV)	1486.789	1486.777	0.012	303	317	1	GFPGLDGEKGVVGQK
collagen alpha 2 (IV)	1589.857	1589.819	0.038	608	624	0	GFPGDIGPPGQGLPGPK
collagen alpha 2 (IV)	1751.855	1751.808	0.047	358	375	1	GARGDPGFQGAHGEPGSR
collagen alpha 2 (IV)	1751.855	1751.752	0.103	1345	1362	0	***GEQGFMGNTGPSGAVGDR***
collagen alpha 2 (IV)	2063.054	2063.093	-0.039	547	566	2	TITTKGERGQPGIPGVHGMK
collagen alpha 2 (IV)	2143.995	2143.989	0.007	715	735	1	***GFPGDPGREGFPGPPGFMGPR***
collagen alpha 2 (IV)	2143.995	2144.111	-0.116	1405	1426	1	RGLPGALGEIGPQGPPGDPGFR
collagen alpha 2 (IV)	2162.056	2162.045	0.011	291	311	1	GEEGIMGFPGIRGFPGLDGEK

Protein Name	Measured Mass (Da)	Calculated Mass (Da)	Error	Peptide Start	Peptide End	Missed cleavage	Sequence
collagen alpha 2 (IV)	2272.082	2272.112	-0.03	552	574	2	GERGQPGIPGVHGMKGDDGVPGR
collagen alpha 2 (IV)	2288.11	2288.107	0.003	552	574	2	***GERGQPGIPGVHGMKGDDGVPGR***
collagen, type XVII, alpha 1	1129.636	1129.599	0.037	1399	1410	1	GPPGPPGPRGNK
collagen, type XVII, alpha 1	1138.647	1138.561	0.086	358	366	0	***EMELLIMTK***
collagen, type XVII, alpha 1	1140.637	1140.613	0.024	696	707	1	GDVGLPGVKGDK
collagen, type XVII, alpha 1	1152.65	1152.595	0.055	705	716	1	GDKGLMGPPGPK
collagen, type XVII, alpha 1	1154.648	1154.567	0.081	1381	1392	1	GDVGTPGPKGDR
collagen, type XVII, alpha 1	1208.676	1208.662	0.014	723	734	1	GPRGLTGEPGIR
collagen, type XVII, alpha 1	1469.751	1469.692	0.059	616	630	0	GSIGDPGMEGPIGQR
collagen, type XVII, alpha 1	1510.751	1510.646	0.104	418	430	1	***SKMTSAENHGYDR***
collagen, type XVII, alpha 1	1655.741	1655.785	-0.045	34	51	1	GSTSNGYAKTGSLGGGSR
cortactin	943.447	943.508	-0.061	391	398	1	LEEQARAK
cortactin	957.472	957.455	0.017	190	198	1	DYSKGFGGK
cortactin	960.484	960.426	0.058	360	366	1	EREQEDR
cortactin	971.475	971.471	0.004	264	272	1	DYKTGFGGK
cortactin	985.481	985.482	-0.001	219	226	1	TEKHESQK
cortactin	985.481	985.482	-0.001	182	189	1	TEKHESQK
cortactin	1086.559	1086.487	0.072	111	119	0	HCSQVDSVR
cortactin	1114.604	1114.504	0.1	296	304	0	HEPQQDYAK
cortactin	1190.558	1190.604	-0.046	352	361	1	ANFENLAKER
cortactin	1215.591	1215.54	0.051	280	290	0	QDSSAVGFDYK
cortactin	1297.643	1297.623	0.02	120	131	1	GFGGKFGVQMDR
cortactin	1426.722	1426.72	0.002	293	304	1	LAKHEPQQDYAK
cortactin	1426.722	1426.709	0.013	206	218	1	VDKSAVGFEYQGK
cortactin	1426.722	1426.672	0.05	132	144	0	VDQSAVGFEYQGK
cortactin	1522.81	1522.683	0.127	95	107	1	***MDRSAVGHEYQSK***
cortactin	1614.801	1614.893	-0.092	337	351	1	TVPIEAVTSKTSNIR
cortactin	1683.82	1683.843	-0.023	43	57	0	TVQGSGHQEHINIHK
cortactin	1953.015	1953.028	-0.013	43	59	1	TVQGSGHQEHINIHKLR
Cyclophilin C	1187.611	1187.586	0.025	173	182	0	TPFVVEVPDW
Cyclophilin C	1465.78	1465.756	0.024	3	15	1	GPSVTDKVFFDVR
Cyclophilin C	1642.855	1642.86	-0.005	169	182	1	IDVKTPFVVEVPDW
Cyclophilin C	1696.878	1696.86	0.018	60	74	1	VIKDFMIQGGDFTAR
Cyclophilin C	1712.894	1712.855	0.039	60	74	1	***VIKDFMIQGGDFTAR***
Cyclophilin C	1945.974	1945.978	-0.004	36	53	1	TVENFVALATGEKGYGYK
DDX46	900.365	900.477	-0.112	804	811	1	SRSTTPPR
DDX46	912.389	912.514	-0.125	640	646	2	RKIEDPR
DDX46	912.389	912.441	-0.052	647	655	0	GNLSGNSHK
DDX46	918.363	918.488	-0.125	743	750	2	NATKDSKR
DDX46	925.403	925.509	-0.106	360	367	2	DAHRKATK
DDX46	931.39	931.506	-0.116	621	627	3	SRSRDRR
DDX46	931.39	931.435	-0.045	658	665	1	GEAKEQDR
DDX46	931.39	931.506	-0.116	634	640	3	SRSRDRR
DDX46	941.443	941.54	-0.097	806	813	2	STTPPRRK
DDX46	945.427	945.535	-0.108	579	586	2	RVKVDSSR
DDX46	945.427	945.499	-0.072	680	686	3	KDKERDR
DDX46	958.459	958.517	-0.058	593	599	3	DRRRSNR
DDX46	962.394	962.5	-0.106	628	635	2	TNRSSRSR
DDX46	975.405	975.509	-0.104	668	675	2	ERSRSVDK
DDX46	975.405	975.455	-0.05	254	260	1	ERMEQQR
DDX46	984.416	984.557	-0.141	798	805	3	HKSKSRSR

Protein Name	Measured Mass (Da)	Calculated Mass (Da)	Error	Peptide Start	Peptide End	Missed cleavage	Sequence
DDX46	986.457	986.5	-0.043	609	616	2	NRSPSRDR
DDX46	997.421	997.505	-0.084	571	578	2	SRSRSYSR
DDX46	1099.468	1099.537	-0.069	734	742	2	HDSRQDSKK
DDX46	1105.479	1105.57	-0.091	631	639	3	SSRSRSRDR
Dedicator of cytokinesis 2	960.5	960.586	-0.086	802	809	1	RLIGFSIR
Dedicator of cytokinesis 2	974.371	974.453	-0.082	265	272	0	***NFAMSYVK***
Dedicator of cytokinesis 2	991.466	991.533	-0.067	17	25	0	VVFTDLGNK
Dedicator of cytokinesis 2	991.466	991.445	0.021	75	83	1	GKAESDEEK
Dedicator of cytokinesis 2	1210.7	1210.696	0.004	475	484	1	TLKALEYVFK
Dedicator of cytokinesis 2	1272.723	1272.617	0.106	262	272	1	GEKNFAMSYVK
Dedicator of cytokinesis 2	1307.659	1307.694	-0.035	312	323	1	HPVENKGATLSR
Dedicator of cytokinesis 2	1428.792	1428.743	0.049	1540	1551	1	***VNQFFKTMLASK***
Dedicator of cytokinesis 2	1469.752	1469.79	-0.038	364	375	0	MKPQLLQENLEK
Dedicator of cytokinesis 2	1547.797	1547.801	-0.004	4	16	1	***GFPKEIEMLNNLK***
Dedicator of cytokinesis 2	1770.861	1770.954	-0.093	451	465	2	***KLMTVLKTYLDTSSR***
Dedicator of cytokinesis 2	1831.888	1831.935	-0.047	537	552	0	YIPSVLHDVETVFDAK
Dedicator of cytokinesis 2	1848.799	1848.939	-0.141	106	123	1	***VIASKGDSGGQGLWVTMK***
Dedicator of cytokinesis 2	1874.975	1875.028	-0.053	60	76	1	***RPFGVAVMDITDIIKGK***
Dedicator of cytokinesis 2	1910.937	1910.915	0.022	923	938	2	LLDYRGVMTDESKDNR
Dedicator of cytokinesis 2	1913.876	1913.822	0.054	721	736	0	***NVYPGDWMAMSMVQNR***
Dedicator of cytokinesis 2	1924.991	1924.952	0.039	276	292	0	EDGTTLHDGYHELVVLK
Dedicator of cytokinesis 2	1924.991	1925.018	-0.027	737	752	2	***VFLRAINKFAETMNQK***
Dedicator of cytokinesis 2	1962.097	1962.115	-0.018	32	47	2	***IFLICQIVRIGKMDLK***
Dedicator of cytokinesis 2	2014.033	2013.909	0.124	1351	1366	2	***VEKEYGVREMPDFEDR***
Dedicator of cytokinesis 2	2144.041	2144.031	0.01	1463	1481	1	KQEFMSDTNLSEHAAIPAR
Dedicator of cytokinesis 2	2160.038	2160.026	0.012	1463	1481	1	***KQEFMSDTNLSEHAAIPAR***
Dedicator of cytokinesis 2	2162.026	2162.035	-0.01	843	859	1	KATIPIFFDMMLCEYQR
dihydrolipoamide branched chain transacylase E2	1005.527	1005.512	0.014	253	261	0	TEPVTGFQK
dihydrolipoamide branched chain transacylase E2	1103.655	1103.575	0.08	179	187	1	***RLAMENNIK***
dihydrolipoamide branched chain transacylase E2	1124.669	1124.655	0.014	292	301	0	EELKPVALAR
dihydrolipoamide branched chain transacylase E2	1128.684	1128.604	0.08	305	313	0	LSFMPFFLK
dihydrolipoamide branched chain transacylase E2	1142.668	1142.594	0.074	47	55	1	YSQPRHSLR
dihydrolipoamide branched chain transacylase E2	1149.667	1149.624	0.042	262	272	1	AMVKTMSAALK
dihydrolipoamide branched chain transacylase E2	1426.801	1426.804	-0.003	302	313	1	GIKLSFMPFFLK
dihydrolipoamide branched chain transacylase E2	1508.824	1508.819	0.005	106	119	1	ASVTITSRYDGVIK
dishevelled associated activator of morphogenesis 1	1130.599	1130.665	-0.066	133	142	1	TIESLKTALR
dishevelled associated activator of morphogenesis 1	1171.59	1171.598	-0.008	955	965	1	AVKHFGEEAGK
dishevelled associated activator of morphogenesis 1	1208.56	1208.592	-0.032	904	913	0	AVETELEYQK
dishevelled associated activator of morphogenesis 1	1348.659	1348.677	-0.018	612	622	1	SFNWSKLPENK
dishevelled associated activator of morphogenesis 1	1504.761	1504.761	0	667	680	1	EADAIDDTLSSKLK
dishevelled associated activator of morphogenesis 1	1516.745	1516.77	-0.025	438	449	1	MLVNENEVKQWK
dishevelled associated activator of morphogenesis 1	1558.756	1558.813	-0.057	434	446	1	***NVVRMLVNENEVK***
dishevelled associated activator of morphogenesis 1	1647.813	1647.849	-0.036	886	899	1	VNMTELDKEISTLR
dishevelled associated activator of morphogenesis 1	1851.909	1851.929	-0.02	240	254	1	***VLQAMLHYQKYASER***
dishevelled associated activator of morphogenesis 1	2142.995	2143.042	-0.047	904	922	1	AVETELEYQKSQPPQPGDK
dishevelled associated activator of morphogenesis 1	2288.111	2288.063	0.047	322	339	1	EHENSTLDRHLDFFEMLR
DNA replication licensing factor MCM5	964.609	964.527	0.082	583	589	1	NRYIIMR
DNA replication licensing factor MCM5	978.607	978.524	0.083	55	61	2	YRDELKR
DNA replication licensing factor MCM5	1046.517	1046.488	0.028	142	150	0	SDMMSHLVK
DNA replication licensing factor MCM5	1108.62	1108.617	0.003	724	731	2	***MQRKVLYR***
DNA replication licensing factor MCM5	1123.677	1123.63	0.047	262	271	0	VTIMGIYSIK

Protein Name	Measured Mass (Da)	Calculated Mass (Da)	Error	Peptide Start	Peptide End	Missed cleavage	Sequence
DNA replication licensing factor MCM5	1139.729	1139.625	0.104	262	271	0	***VTIMGIYSIK***
DNA replication licensing factor MCM5	1205.714	1205.688	0.026	284	294	1	VGVGIRSSYIR
DNA replication licensing factor MCM5	1205.714	1205.706	0.008	581	589	2	LKNRYIIMR
DNA replication licensing factor MCM5	1302.751	1302.718	0.032	163	173	2	AKATRISIQCR
DNA replication licensing factor MCM5	1328.791	1328.741	0.05	573	584	2	LSAEAAEKLKNR
DNA replication licensing factor MCM5	1432.889	1432.741	0.148	139	150	1	***ILKSDMMSHLVK***
DNA replication licensing factor MCM5	1437.85	1437.773	0.077	717	727	3	RGEIQHRMQRK
DNA replication licensing factor MCM5	1443.795	1443.833	-0.038	324	336	0	LAALPNIYELISK
DNA replication licensing factor MCM5	1453.816	1453.768	0.048	717	727	3	***RGEIQHRMQRK***
DNA replication licensing factor MCM5	1496.723	1496.736	-0.013	337	350	1	***SISPSIFGGMDMKK***
DNA replication licensing factor MCM5	1576.855	1576.898	-0.043	581	593	3	LKNRYIIMRSGAR
DNA replication licensing factor MCM5	1592.953	1592.893	0.06	581	593	3	LKNRYIIMRSGAR
dystonin	1312.658	1312.709	-0.051	50	60	1	AQVQELNDRLK
dystonin	1403.737	1403.653	0.084	365	376	0	QCGMHTEVTTLK
dystonin	1424.719	1424.762	-0.043	669	680	1	EISNLNIEKTHK
dystonin	1571.817	1571.805	0.012	132	144	1	AQENAKLWETNIR
dystonin	1645.829	1645.791	0.038	363	376	1	NKQCGMHTEVTTLK
dystonin	1651.794	1651.755	0.039	380	394	1	***RLGSSAGGWMLEGCR***
dystonin	1711.789	1711.856	-0.067	706	719	0	GIYLELSGQQYQWK
dystonin	1788.896	1788.849	0.047	365	379	1	QCGMHTEVTTLKQEK
dystonin	1948.91	1948.973	-0.063	739	756	0	TGLQFNISEAVEQGTLDK
dystonin	1957.935	1957.888	0.047	157	172	1	***MQQGPPVEANHYQKCR***
dystonin	2225.084	2225.067	0.017	877	896	1	***MMSVVEAVNANIISKEMGMR***
dystonin	2257.069	2257.056	0.012	877	896	1	***MMSVVEAVNANIISKEMGMR***
dystonin	2273.093	2273.051	0.042	877	896	1	***MMSVVEAVNANIISKEMGMR***
dystonin	2324.111	2324.157	-0.046	556	576	1	***AVLGYSHASKTLSVFQAMENR***
dystonin isoform 1A	984.519	984.546	-0.027	621	629	1	KTPSRPGSR
dystonin isoform 1A	1156.679	1156.645	0.034	212	221	2	KQPDVDKVTK
dystonin isoform 1A	1174.686	1174.623	0.063	472	481	2	AKGRTNMELR
dystonin isoform 1A	1199.687	1199.636	0.051	622	633	1	TPSRPGSRAGSK
dystonin isoform 1A	1208.704	1208.622	0.082	93	101	2	EFMKRLEEK
dystonin isoform 1A	1402.79	1402.727	0.063	86	96	1	TLIEQHKEFMK
dystonin isoform 1A	1426.79	1426.75	0.04	503	515	2	RSRPSSRGASPNR
dystonin isoform 1A	1835.833	1835.854	-0.021	197	211	0	TLIAEHQTFMEEMTR
dystonin isoform 1A	1851.837	1851.848	-0.011	197	211	0	***TLIAEHQTFMEEMTR***
Enolase 1 (phosphopyruvate hydratase)	931.553	931.524	0.029	427	434	1	SFRNPLAK
Enolase 1 (phosphopyruvate hydratase)	1111.54	1111.628	-0.088	1	9	1	***MSILRIHAR***
Enolase 1 (phosphopyruvate hydratase)	1142.58	1142.608	-0.028	184	193	0	IGAEVYHNLK
Enolase 1 (phosphopyruvate hydratase)	1180.617	1180.62	-0.003	61	71	0	GVSQAVEHINK
Enolase 1 (phosphopyruvate hydratase)	1690.883	1690.888	-0.005	407	420	1	YNQILRIEEELGSK
Enolase 1 (phosphopyruvate hydratase)	1803.907	1803.936	-0.029	33	50	0	AAVPSGASTGIYEALELR
Enolase 1 (phosphopyruvate hydratase)	1927.927	1927.953	-0.026	163	179	0	***LAMQEFMILPVGASSFR***
Enolase 1 (phosphopyruvate hydratase)	2153.056	2153.063	-0.007	10	28	1	EIFDSRGNPTVEVDLYTAK
Enolase 1 (phosphopyruvate hydratase)	2207.02	2207.02	0	234	253	0	***AGYTDQVVIGMDVAASEFYR***
Enolase 3, beta	1474.774	1474.777	-0.003	413	426	1	IEEALGDKAVFAGR
Enolase 3, beta	1592.817	1592.826	-0.009	16	30	1	GNPTVEVDLHTAKGR
Enolase 3, beta	1694.812	1694.829	-0.017	407	420	1	***YNQLMRIEEALGDK***
Enolase 3, beta	1803.892	1803.936	-0.044	33	50	0	AAVPSGASTGIYEALELR
Enolase 3, beta	2093.054	2093.074	-0.021	10	28	1	EILDSRGNPTVEVDLHTAK
Enolase 3, beta	2219.007	2219.106	-0.099	33	54	1	AAVPSGASTGIYEALELRDGDK
Enolase 3, beta	2687.298	2687.325	-0.028	229	253	1	***TAIQAAGYPDKVVIGMDVAASEFYR***

Protein Name	Measured Mass (Da)	Calculated Mass (Da)	Error	Peptide Start	Peptide End	Missed cleavage	Sequence
Epidermal growth factor receptor kinase substrate 8	1125.651	1125.65	0.001	309	319	1	KGPGEGVLTLR
Epidermal growth factor receptor kinase substrate 8	1173.719	1173.665	0.053	335	344	1	FKHGFNLLAK
Epidermal growth factor receptor kinase substrate 8	1173.719	1173.661	0.057	674	682	2	YKQLPVDRR
Epidermal growth factor receptor kinase substrate 8	1199.68	1199.709	-0.029	697	707	2	LTIGRSAAQRK
Epidermal growth factor receptor kinase substrate 8	1199.68	1199.68	0	195	204	1	***RPEALRMIAK***
Epidermal growth factor receptor kinase substrate 8	1295.785	1295.721	0.064	702	712	2	SAAQRKFHVPR
Epidermal growth factor receptor kinase substrate 8	1414.838	1414.844	-0.006	335	346	2	FKHGFNLLAKLK
Epidermal growth factor receptor kinase substrate 8	1587.735	1587.865	-0.13	79	92	2	DAMITVEDGIRKLK
Epidermal growth factor receptor kinase substrate 8	1690.765	1690.825	-0.06	173	188	0	ANLISEDIESAISDSK
Epidermal growth factor receptor kinase substrate 8	1773.767	1773.839	-0.072	320	334	0	AKPPPPDEFVDCFQK
Epidermal growth factor receptor kinase substrate 8	1851.805	1851.775	0.03	803	821	0	ISAAASDSGVESFDEGSSH
fascin	1199.738	1199.666	0.072	469	479	1	YLKGDHAGVLK
fascin	1268.778	1268.637	0.141	101	110	1	WSLQSEAHRR
fascin	1610.853	1610.735	0.118	69	82	1	YLAADKDGNVTCER
fascin	1734.732	1734.676	0.056	331	343	0	NASCYFDIEWCDR
fascin	1770.847	1770.878	-0.031	314	330	0	YWTLTATGGVQSTASTK
fascin	1802.904	1802.967	-0.063	202	217	0	LVARPEPATGFTLEFR
fascin	1890.726	1890.777	-0.051	331	344	1	NASCYFDIEWCDRR
fibrinogen B-beta-chain	1670.806	1670.707	0.099	203	215	0	***YYWGGLYSWDMSK***
fibrinogen B-beta-chain	1670.806	1670.739	0.067	72	85	0	YCGLPGEYWLGNDK
fibrinogen B-beta-chain	1706.887	1706.788	0.099	92	105	0	***MGPTELLIEMEDWK***
fibrinogen B-beta-chain	1819.876	1819.801	0.074	153	167	0	TMTIHNGMFFSTYDR
fibrinogen B-beta-chain	1835.919	1835.796	0.122	153	167	0	***TMTIHNGMFFSTYDR***
fibrinogen B-beta-chain	1851.895	1851.791	0.104	153	167	0	***TMTIHNGMFFSTYDR***
fibrinogen B-beta-chain	1876.01	1875.874	0.136	134	152	0	***GTAGNALMDGASQLVGENR***
fibrinogen, B beta polypeptide	1819.827	1819.801	0.026	386	400	0	TMTIHNGMFFSTYDR
fibrinogen, B beta polypeptide	1835.839	1835.796	0.042	386	400	0	***TMTIHNGMFFSTYDR***
fibrinogen, B beta polypeptide	1851.84	1851.791	0.049	386	400	0	***TMTIHNGMFFSTYDR***
fibrinogen, B beta polypeptide	1875.862	1875.874	-0.012	367	385	0	***GTAGNALMDGASQLVGENR***
fibrinogen, B beta polypeptide	1900.899	1900.989	-0.09	171	186	0	LYIDETVNDNIPLNLR
fibrinogen, B beta polypeptide	1975.918	1976.01	-0.093	44	62	0	EEPPSLRPAPPPISGGGYR
flt-1	992.517	992.482	0.034	821	827	1	WEFARER
flt-1	1022.562	1022.558	0.004	1219	1226	1	FMSLERIK
flt-1	1110.624	1110.571	0.053	1018	1026	1	CIHRDLAAR
flt-1	1154.651	1154.611	0.04	934	943	1	DAALHMELKK
flt-1	1211.675	1211.607	0.068	600	609	1	***TMHHSISKQK***
flt-1	1242.689	1242.675	0.013	687	697	0	GVPAPQITWFK
flt-1	1292.745	1292.687	0.058	1257	1266	1	RFTWTETKPK
flt-1	1512.898	1512.87	0.028	360	372	1	VKAFPSPEIVWLK
flt-1	1917.023	1916.948	0.075	927	942	1	DLFCLNKDAALHMELK
flt-1	1974.046	1973.992	0.054	38	54	1	GTQHVMQAGQTLFLKCR
FYVE and coiled-coil domain containing 1	972.447	972.462	-0.015	791	798	0	EQNEALNR
FYVE and coiled-coil domain containing 1	1002.428	1002.513	-0.085	1106	1113	1	DKDALWQK
FYVE and coiled-coil domain containing 1	1114.616	1114.62	-0.004	454	462	2	KQESAQLRR
FYVE and coiled-coil domain containing 1	1114.616	1114.598	0.018	425	433	1	ELQLKEEAR
FYVE and coiled-coil domain containing 1	1190.574	1190.56	0.014	1082	1091	0	***MLADLDDLNR***
FYVE and coiled-coil domain containing 1	1307.671	1307.66	0.011	811	821	0	EGILQEESIYK
FYVE and coiled-coil domain containing 1	1312.688	1312.687	0.001	442	453	0	DVVPLQEELSGK
FYVE and coiled-coil domain containing 1	1445.818	1445.823	-0.006	1095	1105	1	YLEERLIELLR
FYVE and coiled-coil domain containing 1	1542.879	1542.807	0.072	905	917	0	GLELQVMQLQQEK
FYVE and coiled-coil domain containing 1	1630.838	1630.797	0.041	989	1002	0	AQLEEQGQQLQMTK

Protein Name	Measured Mass (Da)	Calculated Mass (Da)	Error	Peptide Start	Peptide End	Missed cleavage	Sequence
FYVE and coiled-coil domain containing 1	1723.843	1723.844	-0.001	326	340	1	***KEYSPSALQLENMAK***
FYVE and coiled-coil domain containing 1	1944.031	1944.034	-0.003	904	919	2	***KGLELQVMQLQQEKEK***
FYVE and coiled-coil domain containing 1	2224.108	2224.191	-0.083	822	840	2	AQKQEQELRALQAELSQVR
FYVE and coiled-coil domain containing 1	2272.149	2272.01	0.139	1160	1177	0	IFCYYCCNNYVVTKPSGK
glyceraldehyde-3-phosphate dehydrogenase	1258.669	1258.593	0.076	322	332	0	***VVDLMAYMASK***
glyceraldehyde-3-phosphate dehydrogenase	1318.715	1318.713	0.002	247	257	1	LEKPAKYDDIK
glyceraldehyde-3-phosphate dehydrogenase	1355.695	1355.646	0.049	322	333	1	VVDLMAYMASKE
glyceraldehyde-3-phosphate dehydrogenase	1555.804	1555.802	0.002	233	246	0	VPTPNVSVVDLTCR
glyceraldehyde-3-phosphate dehydrogenase	1778.742	1778.789	-0.047	308	321	0	LISWYDNEYGYSNR
glyceraldehyde-3-phosphate dehydrogenase	1818.833	1818.896	-0.063	144	160	0	IVSNASCTTNCLAPLAK
glyceraldehyde-3-phosphate dehydrogenase	2227.992	2228.096	-0.104	117	137	0	***VIISAPSADAPMFVMGVNHEK***
glyceraldehyde-3-phosphate dehydrogenase	2244.026	2244.091	-0.065	117	137	0	***VIISAPSADAPMFVMGVNHEK***
glyceraldehyde-3-phosphate dehydrogenase	2348.167	2348.197	-0.031	226	246	1	***LTGMAFRVPTPNVSVVDLTCR***
glyceraldehyde-3-phosphate dehydrogenase	2400.171	2400.192	-0.021	116	137	1	***RVIISAPSADAPMFVMGVNHEK***
golgi-specific brefeldin A-resistance factor 1	1113.704	1113.675	0.029	693	701	2	ELIEIKNKK
golgi-specific brefeldin A-resistance factor 1	1136.679	1136.593	0.086	383	392	1	GVRFTQSSQK
golgi-specific brefeldin A-resistance factor 1	1313.795	1313.73	0.065	597	607	1	VLNTLNQQEKK
golgi-specific brefeldin A-resistance factor 1	1429.796	1429.784	0.012	502	513	1	***KLMEIITVENPK***
golgi-specific brefeldin A-resistance factor 1	1544.807	1544.801	0.006	393	406	0	EGTALVPYGLPCIR
golgi-specific brefeldin A-resistance factor 1	1722.782	1722.843	-0.061	23	36	1	NARWSTHIPLDEER
golgi-specific brefeldin A-resistance factor 1	1737.862	1737.816	0.046	664	679	2	DLEEAGDSGADKKFTR
golgi-specific brefeldin A-resistance factor 1	1807.864	1807.91	-0.046	480	493	1	VCFLLFESMREHLK
golgi-specific brefeldin A-resistance factor 1	1947.893	1947.929	-0.036	456	470	1	***DEMCRHLFQLLSVER***
Gprin1 protein	1516.668	1516.698	-0.03	920	932	2	RPRCCSRAGPTAE
Gprin1 protein	1520.747	1520.685	0.062	648	661	0	QSDGTPYSSAQPQR
Gprin1 protein	1554.794	1554.727	0.066	3	16	1	DCCSSPKAIPAPPR
Gprin1 protein	1560.814	1560.8	0.014	691	706	1	SPSAEAAAPPPGPRTR
Gprin1 protein	1565.792	1565.866	-0.074	601	615	1	KVDPPTTVEPVSLGK
Gprin1 protein	1599.775	1599.76	0.015	202	214	2	MDPKTENVMHSRR
Gprin1 protein	1603.779	1603.775	0.004	491	506	0	AETVSPGEVDAMTLGK
Gprin1 protein	1607.741	1607.745	-0.004	122	135	1	***EEAGSLRNEESMLK***
Gprin1 protein	1757.829	1757.925	-0.096	913	926	2	ALLQSVRRPRCCSR
Gprin1 protein	1772.844	1772.89	-0.046	665	681	1	SIGSLPEREPSASTSQK
Gprin1 protein	1772.844	1772.89	-0.046	673	690	1	EPSASTSQKDLAAAAAQK
Gprin1 protein	1776.88	1776.867	0.013	122	137	2	EEAGSLRNEESMLKGK
Gprin1 protein	1841.85	1841.869	-0.019	1	16	2	MRDCCSSPKAIPAPPR
Gprin1 protein	1892.853	1892.841	0.012	230	246	1	***ENDMKPPDNTDSASTKK***
Gprin1 protein	1892.853	1892.86	-0.007	648	664	1	QSDGTPYSSAQPQRDTR
Gprin1 protein	2082.938	2083.023	-0.085	705	723	2	TRDNFTKAPSWDAGAPPPR
GRIP and coiled-coil domain-containing protein 2	1209.602	1209.635	-0.033	264	273	1	EHEAEINKLK
GRIP and coiled-coil domain-containing protein 2	1334.658	1334.692	-0.034	1554	1565	1	EELAEKLSSTTK
GRIP and coiled-coil domain-containing protein 2	1348.726	1348.698	0.028	469	479	0	QEVVLNYESLR
GRIP and coiled-coil domain-containing protein 2	1560.768	1560.85	-0.083	735	747	1	LLENEQVQKSFVK
GRIP and coiled-coil domain-containing protein 2	1562.78	1562.797	-0.017	1387	1399	1	***MLQETVTKEAELR***
GRIP and coiled-coil domain-containing protein 2	1585.763	1585.819	-0.056	1088	1101	1	GVVEKELDAEELQK
GRIP and coiled-coil domain-containing protein 2	1606.741	1606.783	-0.042	1242	1255	0	GELEASQQQVEVYK
GRIP and coiled-coil domain-containing protein 2	1634.742	1634.792	-0.051	653	666	1	EKSQNDQSITVQMK
GRIP and coiled-coil domain-containing protein 2	1692.746	1692.8	-0.055	1665	1679	0	SSGWASYLHSWSGLR
GRIP and coiled-coil domain-containing protein 2	1713.771	1713.787	-0.016	1	18	0	MEDSAPDAVAAAPSGTPK
GRIP and coiled-coil domain-containing protein 2	1729.76	1729.782	-0.022	1	18	0	***MEDSAPDAVAAAPSGTPK***
GRIP and coiled-coil domain-containing protein 2	1751.813	1751.85	-0.037	558	573	0	NLQADNSMYLASLGQK

Protein Name	Measured Mass (Da)	Calculated Mass (Da)	Error	Peptide Start	Peptide End	Missed cleavage	Sequence
GRIP and coiled-coil domain-containing protein 2	1828.795	1828.884	-0.089	1299	1315	1	AAKAEQAAVTSEFESYK
GRIP and coiled-coil domain-containing protein 2	1897.852	1897.915	-0.063	431	447	1	EVSELSETFISGSEKEK
GRIP and coiled-coil domain-containing protein 2	1903.888	1903.955	-0.067	846	861	1	***EALQLDLLEMKNTNEK***
GRIP and coiled-coil domain-containing protein 2	1944.889	1944.909	-0.02	1	20	1	***MEDSAPDAVAAAPSGTPKSK***
Grp94 neighboring nucleotidase variant 3	963.465	963.444	0.021	49	56	0	LNDICSSR
Grp94 neighboring nucleotidase variant 3	965.471	965.438	0.033	553	559	0	NEFQCLR
Grp94 neighboring nucleotidase variant 3	975.452	975.517	-0.065	148	155	0	NGYPWVLK
Grp94 neighboring nucleotidase variant 3	977.478	977.481	-0.003	789	796	0	IEGEQFQK
Grp94 neighboring nucleotidase variant 3	985.482	985.461	0.021	75	82	0	SFTSNQFR
Grp94 neighboring nucleotidase variant 3	990.467	990.523	-0.056	1273	1281	0	SISELVSEK
Grp94 neighboring nucleotidase variant 3	1333.61	1333.622	-0.012	236	246	0	DLEELGDMVWK
Grp94 neighboring nucleotidase variant 3	1390.715	1390.666	0.049	83	93	0	QYSCLQSQHLK
GTP binding protein 4	1112.741	1112.666	0.075	481	489	2	QLAKQIREK
GTP binding protein 4	1124.75	1124.604	0.146	590	598	3	***TMMKKAQKK***
GTP binding protein 4	1149.712	1149.623	0.089	42	49	2	IRHFYMRK
GTP binding protein 4	1195.754	1195.641	0.113	588	597	3	***AKTMMKKAQK***
GTP binding protein 4	1204.781	1204.653	0.128	136	144	2	MCTIIKRQR
GTP binding protein 4	1240.798	1240.761	0.037	481	490	3	QLAKQIREKK
GTP binding protein 4	1268.799	1268.713	0.086	503	513	3	QGPRMPRTAKK
GTP binding protein 4	1284.848	1284.708	0.14	503	513	3	***QGPRMPRTAKK***
GTP binding protein 4	1307.861	1307.767	0.094	25	35	2	TQRKTPTVIHK
GTP binding protein 4	1341.881	1341.736	0.145	572	583	2	TPRDVSGLRDVK
GTP binding protein 4	1382.874	1382.756	0.118	501	512	3	NKQGPRMPRTAK
GTP binding protein 4	1398.895	1398.751	0.144	501	512	3	***NKQGPRMPRTAK***
GTP binding protein 4	1416.875	1416.75	0.125	347	358	2	***MKGNKVNEVLNR***
GTP binding protein 4	1489.92	1489.828	0.092	575	587	3	***DVSGLRDVKMVKK***
GTP binding protein 4	1541.858	1541.792	0.066	534	546	2	NNAHYAVQARRSR
GTP binding protein 4	1656.8	1656.84	-0.04	605	618	2	KGEADRHVFDMKPK
guanine nucleotide exchange factor 1	1098.573	1098.604	-0.031	1477	1484	2	ENFRRHIK
guanine nucleotide exchange factor 1	1373.752	1373.81	-0.058	748	759	3	RTLSLTQRGKSK
guanine nucleotide exchange factor 1	1444.818	1444.799	0.019	628	639	2	EDTVRLLKSQTR
guanine nucleotide exchange factor 1	1564.916	1564.897	0.019	1365	1377	2	ARKDIELTVFVFK
guanine nucleotide exchange factor 1	1597.751	1597.778	-0.027	378	390	2	MRRFSDWTGSLSR
guanine nucleotide exchange factor 1	1604.949	1604.924	0.025	761	775	2	GIFSSLKGLDTLARK
guanine nucleotide exchange factor 1	1604.949	1604.924	0.025	760	774	2	KGIFSSLKGLDTLAR
guanine nucleotide exchange factor 1	1629.963	1629.904	0.059	363	377	3	ALTEDAAKKDTLKAR
guanine nucleotide exchange factor 1	1680.005	1679.894	0.111	640	653	3	***SLLQKIDMDSKMKK***
guanine nucleotide exchange factor 1	1712.798	1712.876	-0.078	78	93	2	LSGPTCKVSKGTTYSK
guanine nucleotide exchange factor 1	1740.873	1740.908	-0.035	863	876	1	MRQLEPTHYGLQLR
guanine nucleotide exchange factor 1	1788.924	1788.98	-0.056	741	755	3	DDSTLRKRTLSLTQR
guanine nucleotide exchange factor 1	1814.006	1813.971	0.035	654	669	2	MAELQLSVVSDPKNRK
guanine nucleotide exchange factor 1	1814.006	1813.971	0.035	653	668	2	KMAELQLSVVSDPKNR
guanine nucleotide exchange factor 1	1821.918	1821.973	-0.055	1241	1256	3	TDKAFKAFLDARNPTK
guanine nucleotide exchange factor 1	1925.954	1925.98	-0.026	1482	1496	3	HIKCELPLEKTCKDR
guanine nucleotide exchange factor 1	2225.103	2225.252	-0.149	1257	1275	1	QHSSTLESYLIKPVQRVLK
guanine nucleotide exchange factor 1	2272.107	2272.127	-0.021	59	77	2	SLARSCLSHFKNHQPYATR
guanine nucleotide exchange factor 1	960.512	960.612	-0.1	1378	1385	1	RAVILVYK
guanine nucleotide exchange factor 1	985.521	985.473	0.048	70	77	0	NHQPYATR
guanylate binding protein 7	1135.654	1135.707	-0.053	543	551	2	LEHKLKIQK
guanylate binding protein 7	1657.753	1657.899	-0.146	575	589	2	EIQLNKEKNSSLGAK
guanylate binding protein 7	1707.736	1707.816	-0.08	561	574	1	***KCEAMDLEISQLQK***

Protein Name	Measured Mass (Da)	Calculated Mass (Da)	Error	Peptide Start	Peptide End	Missed cleavage	Sequence
guanylate binding protein 7	1712.741	1712.814	-0.073	519	532	1	ENVAQLHEKMETER
guanylate binding protein 7	1718.802	1718.949	-0.147	547	560	3	LKIQKDMLNEGFKR
guanylate binding protein 7	1728.751	1728.809	-0.058	519	532	1	***ENVAQLHEKMETER***
guanylate binding protein 7	1737.732	1737.848	-0.116	71	84	0	***GIWMWCVPHPSKPK***
guanylate binding protein 7	1737.732	1737.835	-0.103	505	518	1	ELQQVMEAQERSYK
guanylate binding protein 7	1747.777	1747.923	-0.146	85	100	0	FTLVLLDTEGLGDVEK
guanylate binding protein 7	1753.742	1753.83	-0.088	505	518	1	***ELQQVMEAQERSYK***
guanylate binding protein 7	1761.725	1761.867	-0.142	474	489	2	***ALTDGQKAMEAERAQK***
guanylate binding protein 7	1946.773	1946.87	-0.098	1	16	0	***MEAPVCLVENENEELR***
guanylate cyclase 2e	963.525	963.496	0.029	970	977	0	HMPEVPVR
guanylate cyclase 2e	1205.664	1205.643	0.021	816	825	1	***KTNIIDSMLR***
guanylate cyclase 2e	1208.575	1208.626	-0.051	638	646	1	EIKLDWMFK
guanylate cyclase 2e	1307.65	1307.65	0	1052	1062	0	GIEDTYWLVGR
guanylate cyclase 2e	1583.779	1583.794	-0.015	317	330	2	RCPPGGSVQDSLRR
guanylate cyclase 2e	1600.743	1600.843	-0.1	367	383	1	ARTAVGGGWVSGASVAR
guanylate cyclase 2e	1735.816	1735.849	-0.033	1030	1043	1	ILRALDQGFQMECR
guanylate cyclase 2e	1817.871	1817.93	-0.059	210	226	1	***ARGLPVALVTSMETSDR***
guanylate cyclase 2e	1821.027	1821.01	0.017	641	655	1	LDWMFKSSLLLDLIK
guanylate cyclase 2e	1821.807	1821.892	-0.085	927	943	0	***VETIGDAYMVASGLPQR***
guanylate cyclase 2e	1895.925	1895.995	-0.07	244	260	0	VVIMVMHSVLLGGEEQR
guanylate cyclase 2e	1991.89	1991.986	-0.096	746	762	0	***STPYAMLELTPEEVIQR***
guanylate cyclase 2e	2037.918	2037.971	-0.053	1033	1049	2	ALDQGFQMECRGRTELK
guanylate cyclase 2e	2053.891	2053.966	-0.075	1033	1049	2	***ALDQGFQMECRGRTELK***
H1 histone family	915.575	915.55	0.025	245	252	2	AKDLVRSK
H1 histone family	915.575	915.525	0.05	34	41	1	RGTQSVLR
H1 histone family	937.505	937.484	0.021	267	274	1	AREQAHAR
H1 histone family	937.505	937.484	0.021	269	276	1	EQAHARAR
H1 histone family	937.505	937.484	0.021	261	268	1	EQAHARAR
H1 histone family	958.554	958.553	0.001	197	204	3	ARSRARSR
H1 histone family	958.554	958.519	0.035	299	306	2	AKEQERAK
H1 histone family	958.554	958.553	0.001	237	244	3	ARSRARSR
H1 histone family	1089.696	1089.6	0.096	169	178	2	VRTRSTSGAR
H1 histone family	1099.688	1099.636	0.052	151	158	3	AREVWRRK
H1 histone family	1099.688	1099.636	0.052	150	157	3	KAREVWRR
H1 histone family	1099.688	1099.636	0.052	153	160	3	EVWRRKAR
H1 histone family	1142.709	1142.688	0.021	245	254	3	AKDLVRSKAR
H1 histone family	1332.812	1332.733	0.079	169	180	3	VRTRSTSGARSR
H1 histone family	1537.793	1537.796	-0.003	223	236	3	SSTRSSAKSWARSK
H1 histone family	1541.782	1541.762	0.02	95	108	1	VSGSDAAGYFRVWK
H1 histone family	1620.883	1620.819	0.064	253	266	2	AREQAQAREQAHAR
H1 histone family	1620.883	1620.819	0.064	255	268	2	EQAQAREQAHARAR
H1 histone family	1880.922	1880.974	-0.052	285	300	2	AQEFVSAKEQQYVRAK
H1 histone family	1880.922	1880.974	-0.052	283	298	2	AKAQEFVSAKEQQYVR
Heat shock protein 1 (chaperonin)	940.58	940.606	-0.026	463	470	1	IGIEIIKR
Heat shock protein 1 (chaperonin)	1096.655	1096.66	-0.005	517	526	1	GIIDPTKVVR
Heat shock protein 1 (chaperonin)	1343.746	1343.708	0.038	61	72	0	TVIIEQSWGSPK
Heat shock protein 1 (chaperonin)	1388.73	1388.697	0.033	222	233	0	GYISPYFINTSK
Heat shock protein 1 (chaperonin)	1683.896	1683.897	-0.001	430	446	0	AAVEEGIVLGGGCALLR
Heat shock protein 1 (chaperonin)	1853.949	1854.047	-0.098	293	310	2	VGLQVVAVKAPGFGDNRK
Heat shock protein 1 (chaperonin)	1937.947	1937.939	0.008	206	221	1	***TLNDELEIIEGMKFDR***
Heat shock protein 1 (chaperonin)	2062.071	2062.153	-0.082	250	268	1	KISSVQSIVPTLEIANAHR

Protein Name	Measured Mass (Da)	Calculated Mass (Da)	Error	Peptide Start	Peptide End	Missed cleavage	Sequence
Heat shock protein 1 (chaperonin)	2573.269	2573.303	-0.034	406	429	3	VGGTSDVEVNEKKDRVTDALNATR
Heat shock protein 1 (chaperonin)	2808.425	2808.401	0.024	371	393	2	IQEITEQLDITTSEYEKEKLNER
Heat shock protein 1 (chaperonin)	2964.518	2964.502	0.015	370	393	3	RIQEITEQLDITTSEYEKEKLNER
Heat shock protein 60	940.58	940.606	-0.026	463	470	1	IGIEIIKR
Heat shock protein 60	1096.655	1096.66	-0.005	517	526	1	GIIDPTKVVR
Heat shock protein 60	1343.746	1343.708	0.038	61	72	0	TVIIEQSWGSPK
Heat shock protein 60	1388.73	1388.697	0.033	222	233	0	GYISPYFINTSK
Heat shock protein 60	1683.896	1683.897	-0.001	430	446	0	AAVEEGIVLGGGCALLR
Heat shock protein 60	1853.949	1854.047	-0.098	293	310	2	VGLQVVAVKAPGFGDNRK
Heat shock protein 60	1937.947	1937.939	0.008	206	221	1	***TLNDELEIIEGMKFDR***
Heat shock protein 60	2559.239	2559.24	-0.001	97	121	0	LVQDVANNTNEEAGDGTTTATVLAR
Heat shock protein 60	2573.269	2573.303	-0.034	406	429	3	VGGTSDVEVNEKKDRVTDALNATR
Heat shock protein 60	2808.425	2808.401	0.024	371	393	2	IQEITEQLDITTSEYEKEKLNER
Heat shock protein 60	2964.518	2964.502	0.015	370	393	3	RIQEITEQLDITTSEYEKEKLNER
Heat shock protein 8	1158.64	1158.587	0.053	558	567	1	LQGKINDEDK
Heat shock protein 8	1406.743	1406.712	0.031	237	247	1	***MVNHFIAEFKR***
Heat shock protein 8	1479.767	1479.746	0.02	300	311	1	ARFEELNADLFR
Heat shock protein 8	1626.879	1626.904	-0.026	156	171	1	QATKDAGTIAGLNVLR
Heat shock protein 8	1690.854	1690.718	0.136	221	236	0	STAGDTHLGGEDFDNR
Heat shock protein 8	1786.894	1786.982	-0.088	172	188	1	IINEPTAAAIAYGLDKK
Heat shock protein 8	1820.88	1820.883	-0.003	57	72	1	***NQVAMNPTNTVFDAKR***
Heat shock protein 8	1836.913	1837.005	-0.092	326	342	1	LDKSQIHDIVLVGGSTR
Heat shock protein 8	1981.004	1980.99	0.014	138	155	0	TVTNAVVTVPAYFNDSQR
heterogeneous nuclear ribonucleoprotein M isoform a	1458.765	1458.695	0.07	591	605	0	***MGPAMGPALGAGIER***
heterogeneous nuclear ribonucleoprotein M isoform a	2051.008	2051.028	-0.02	362	380	1	FGSGMNMGRINEILSNALK
heterogeneous nuclear ribonucleoprotein M isoform a	2161.989	2162.04	-0.051	627	650	1	GNFGGSFAGSFGGAGGHAPGVARK
heterogeneous nuclear ribonucleoprotein M isoform a	2225.036	2225.026	0.009	322	344	0	***GIGMGNLGPAGMGMEGIGFGINK***
heterogeneous nuclear ribonucleoprotein M isoform a	2272.053	2272.042	0.011	436	455	2	***MGLVMDRMGSVERMGSSIER***
heterogeneous nuclear ribonucleoprotein M isoform a	2272.053	2272.14	-0.088	496	516	2	MAAPIDRVGQTIERMGSGVER
heterogeneous nuclear ribonucleoprotein M isoform a	2272.053	2272.14	-0.088	503	523	2	VGQTIERMGSGVERMGPAIER
heterogeneous nuclear ribonucleoprotein M isoform a	2288.094	2288.037	0.057	436	455	2	***MGLVMDRMGSVERMGSSIER***
heterogeneous nuclear ribonucleoprotein M isoform a	2288.094	2288.135	-0.042	496	516	2	***MAAPIDRVGQTIERMGSGVER***
heterogeneous nuclear ribonucleoprotein M isoform a	2288.094	2288.135	-0.042	503	523	2	***VGQTIERMGSGVERMGPAIER***
heterogeneous nuclear ribonucleoprotein M isoform a	2304.029	2304.032	-0.003	436	455	2	***MGLVMDRMGSVERMGSSIER***
heterogeneous nuclear ribonucleoprotein M isoform a	2792.373	2792.325	0.048	531	556	2	MVPTGMGASLERMGPVMDRMATGLER
heterogeneous nuclear ribonucleoprotein M isoform a	2792.373	2792.325	0.048	517	542	2	MGPAIERMGLSMDRMVPTGMGASLER
heterogeneous nuclear ribonucleoprotein M isoform a	2824.289	2824.315	-0.026	531	556	2	***MVPTGMGASLERMGPVMDRMATGLER***
heterogeneous nuclear ribonucleoprotein M isoform a	2824.289	2824.297	-0.008	524	549	2	MGLSMDRMVPTGMGASLERMGPVMDR
heterogeneous nuclear ribonucleoprotein M isoform a	2824.289	2824.315	-0.026	517	542	2	***MGPAIERMGLSMDRMVPTGMGASLER***
high density lipoprotein binding protein	928.575	928.545	0.03	530	536	2	IREIRDK
high density lipoprotein binding protein	1098.636	1098.629	0.007	518	526	1	FHRTIIGQK
high density lipoprotein binding protein	1379.794	1379.826	-0.032	814	824	2	HFVIRRGQVLR
high density lipoprotein binding protein	1689.875	1689.883	-0.008	727	740	1	SFTVDIRAKPEYHK
high density lipoprotein binding protein	1851.993	1852.02	-0.027	73	88	1	ASVITQVFHVPLEERK
high density lipoprotein binding protein	1851.993	1851.979	0.014	478	494	1	SNLIRIEGDPQGVQQAK
high density lipoprotein binding protein	1989.053	1989.052	0.001	266	283	1	TEIVFTGEKEQLAQAVAR
high density lipoprotein binding protein	2047.097	2047.084	0.013	724	740	2	QTKSFTVDIRAKPEYHK
high density lipoprotein binding protein	2162.131	2162.075	0.056	1221	1240	2	APSKGFVVRDAPWTSNSSEK
high density lipoprotein binding protein	2255.2	2255.194	0.006	532	550	2	EIRDKFPEVIINFPDPAQK
high density lipoprotein binding protein	2255.2	2255.226	-0.026	715	733	2	QLLHLAEEKQTKSFTVDIR
high density lipoprotein binding protein	2270.167	2270.193	-0.026	111	132	1	***TGAHLELSLAKDQGLSIMVSGK***

Protein Name	Measured Mass (Da)	Calculated Mass (Da)	Error	Peptide Start	Peptide End	Missed cleavage	Sequence
high density lipoprotein binding protein	2396.233	2396.226	0.007	51	72	1	AACLESAQEPAGAWSNKIRPIK
high density lipoprotein binding protein	2443.186	2443.206	-0.02	430	448	2	***DLINRMDYVEINIDHKFHR***
high density lipoprotein binding protein	2456.275	2456.364	-0.089	147	168	2	LQTQASATVPIPKEHHRFVIGK
high density lipoprotein binding protein	2577.302	2577.285	0.017	569	590	1	***YMQKMVADLVENSYSISVPIFK***
high density lipoprotein binding protein	2618.392	2618.365	0.027	471	494	2	IPPDSEKSNLIRIEGDPQGVQQAK
Histone deacetylase 2	1876.079	1876.013	0.066	36	50	1	IRMTHNLLLNYGLYR
Histone deacetylase 2	1930.916	1930.861	0.055	60	75	1	***ATAEEMTKYHSDEYIK***
Histone deacetylase 2	1984.971	1984.909	0.062	79	94	1	***SIRPDNMSEYSKQMQR***
Histone deacetylase 2	1991.033	1990.979	0.054	128	145	1	QQTDMAVNWAGGLHHAKK
HSP27	1148.656	1148.597	0.059	29	38	0	LFDQAFGVPR
HSP27	1654.819	1654.744	0.075	116	128	1	HEERQDEHGYISR
HSP27	1796.961	1796.93	0.031	89	104	0	VSLDVNHFAPEELTVK
HSP27	1832.006	1831.967	0.039	164	180	0	AVTQSAEITIPVTFEAR
HSP27	2026.107	2026.073	0.034	89	106	1	VSLDVNHFAPEELTVKTK
HSP27	2567.305	2567.235	0.07	107	128	2	EGVVEITGKHEERQDEHGYISR
HSP27	2567.305	2567.235	0.07	107	128	2	EGVVEITGKHEERQDEHGYISR
HSP27-related protein (form b)	931.509	931.462	0.047	93	99	1	QTADRWR
HSP27-related protein (form b)	986.541	986.602	-0.061	5	12	1	RVPFSLLR
HSP27-related protein (form b)	1148.605	1148.597	0.007	29	38	0	LFDQAFGVPR
HSP27-related protein (form b)	1796.898	1796.93	-0.032	100	115	0	VSLDVNHFAPEELTVK
HSP27-related protein (form b)	1831.937	1831.967	-0.03	175	191	0	AVTQSAEITIPVTFEAR
hydroxysteroid (17-beta) dehydrogenase 4	1148.581	1148.576	0.005	1	10	1	MASPLRFDGR
hydroxysteroid (17-beta) dehydrogenase 4	1164.582	1164.57	0.012	1	10	1	***MASPLRFDGR***
hydroxysteroid (17-beta) dehydrogenase 4	1178.595	1178.556	0.039	669	680	0	SGSGEVYQGPAK
hydroxysteroid (17-beta) dehydrogenase 4	1301.705	1301.693	0.011	51	63	1	GSSAADKVVAEIR
hydroxysteroid (17-beta) dehydrogenase 4	1314.707	1314.672	0.035	645	655	0	ANAVFEWHITK
hydroxysteroid (17-beta) dehydrogenase 4	1416.758	1416.761	-0.003	33	46	0	GALVIVNDLGGDFK
hydroxysteroid (17-beta) dehydrogenase 4	1637.824	1637.768	0.055	185	199	0	NNIHCNTIAPNAGSR
hydroxysteroid (17-beta) dehydrogenase 4	2366.218	2366.12	0.098	146	168	0	***ILMTSSASGIYGNFGQANYSAAK***
hydroxysteroid (17-beta) dehydrogenase 4	2887.395	2887.433	-0.038	424	450	1	CEAVIADILDKGSGVVIVMDVYSYSGK
hydroxysteroid (17-beta) dehydrogenase 4	2903.384	2903.428	-0.044	424	450	1	***CEAVIADILDKGSGVVIVMDVYSYSGK***
hypothetical protein LOC66625 isoform 1	900.365	900.477	-0.112	804	811	1	SRSTTPPR
hypothetical protein LOC66625 isoform 1	912.389	912.514	-0.125	640	646	2	RKIEDPR
hypothetical protein LOC66625 isoform 1	912.389	912.441	-0.052	647	655	0	GNLSGNSHK
hypothetical protein LOC66625 isoform 1	918.363	918.488	-0.125	743	750	2	NATKDSKR
hypothetical protein LOC66625 isoform 1	925.403	925.509	-0.106	360	367	2	DAHRKATK
hypothetical protein LOC66625 isoform 1	931.39	931.506	-0.116	621	627	3	SRSRDRR
hypothetical protein LOC66625 isoform 1	931.39	931.435	-0.045	658	665	1	GEAKEQDR
hypothetical protein LOC66625 isoform 1	931.39	931.506	-0.116	634	640	3	SRSRDRR
hypothetical protein LOC66625 isoform 1	941.443	941.54	-0.097	806	813	2	STTPPRRK
hypothetical protein LOC66625 isoform 1	945.427	945.535	-0.108	579	586	2	RVKVDSSR
hypothetical protein LOC66625 isoform 1	945.427	945.499	-0.072	680	686	3	KDKERDR
hypothetical protein LOC66625 isoform 1	958.459	958.517	-0.058	593	599	3	DRRRSNR
hypothetical protein LOC66625 isoform 1	962.394	962.5	-0.106	628	635	2	TNRSSRSR
hypothetical protein LOC66625 isoform 1	975.405	975.509	-0.104	668	675	2	ERSRSVDK
hypothetical protein LOC66625 isoform 1	975.405	975.455	-0.05	254	260	1	ERMEQQR
hypothetical protein LOC66625 isoform 1	984.416	984.557	-0.141	798	805	3	HKSKSRSR
hypothetical protein LOC66625 isoform 1	986.457	986.5	-0.043	609	616	2	NRSPSRDR
hypothetical protein LOC66625 isoform 1	997.421	997.505	-0.084	571	578	2	SRSRSYSR
hypothetical protein LOC66625 isoform 1	1099.468	1099.537	-0.069	734	742	2	HDSRQDSKK
hypothetical protein LOC66625 isoform 1	1105.479	1105.57	-0.091	631	639	3	SSRSRSRDR

Protein Name	Measured Mass (Da)	Calculated Mass (Da)	Error	Peptide Start	Peptide End	Missed cleavage	Sequence
Ikbkb protein	1335.674	1335.554	0.12	516	526	0	EMEQAVEQCGR
Ikbkb protein	1436.707	1436.711	-0.004	468	480	1	***MKNAMASTAQQLK***
Ikbkb protein	1544.753	1544.735	0.018	497	509	1	YKEQTEFGITSDK
Ikbkb protein	1549.729	1549.678	0.051	580	592	1	DQRTEGDSQEMVR
Ikbkb protein	1580.731	1580.846	-0.115	429	441	0	VWGQVWHSIQTLK
Ikbkb protein	1627.791	1627.914	-0.123	119	134	0	EGAVLTLLSDIASALR
Ikbkb protein	1630.788	1630.815	-0.027	719	732	1	LESALQDTVKEQDR
Ikbkb protein	1633.746	1633.838	-0.093	447	460	1	***LQQGQRAAMMSLLR***
Ikbkb protein	1659.747	1659.799	-0.052	665	680	1	***VRGPVSGSPDSMNVSR***
Ikbkb protein	1694.835	1694.826	0.009	453	467	1	AAMMSLLRNNSCLSK
Ikbkb protein	1793.814	1793.933	-0.119	276	288	1	WLQLMLMWHPRQR
Ikbkb protein	1879.863	1879.994	-0.131	273	286	1	LEKWLQLMLMWHPR
Ikbkb protein	1936.918	1936.824	0.094	516	531	1	***EMEQAVEQCGRENDVK***
Ikbkb protein	1938.937	1938.947	-0.01	32	47	1	WHNQATGEQIAIKQCR
Iqgap2 protein	1098.667	1098.614	0.053	815	823	1	IRANQQLEK
Iqgap2 protein	1150.701	1150.598	0.103	91	99	1	KIYDVEQTR
Iqgap2 protein	1287.777	1287.682	0.095	656	666	0	LWSASEDLLVR
Iqgap2 protein	1313.795	1313.661	0.134	92	101	1	IYDVEQTRYK
Iqgap2 protein	1544.807	1544.819	-0.012	654	666	1	EKLWSASEDLLVR
Iqgap2 protein	1655.793	1655.822	-0.029	185	198	1	***ELEKYGIQMPAFSK***
Iqgap2 protein	1771.838	1771.913	-0.075	817	831	1	ANQQLEKDLNLMDIK
Iqgap2 protein	1786.836	1786.914	-0.078	145	158	0	***MIYCIHALSLYLFK***
Iqgap2 protein	1807.864	1807.855	0.009	34	48	0	QNIAYGYLCHLEEAK
Iqgap2 protein	1963.957	1963.956	0.001	34	49	1	QNIAYGYLCHLEEAKR
Iqgap2 protein	1963.957	1963.956	0.001	33	48	1	RQNIAYGYLCHLEEAK
Iqgap2 protein	1972.897	1973.003	-0.106	537	552	1	QWVTLVVDVNECLDRK
Isopentenyl-diphosphate delta isomerase	1255.596	1255.561	0.035	38	47	0	NCHLNENIDK
Isopentenyl-diphosphate delta isomerase	1352.705	1352.693	0.012	158	169	0	NVTLNPDPNEIK
Isopentenyl-diphosphate delta isomerase	1383.682	1383.656	0.026	37	47	1	KNCHLNENIDK
Isopentenyl-diphosphate delta isomerase	1415.723	1415.708	0.015	53	64	0	AFSVFLFNTENK
Isopentenyl-diphosphate delta isomerase	1418.669	1418.649	0.02	170	180	1	SYCYVSKEEVR
Isopentenyl-diphosphate delta isomerase	1480.784	1480.788	-0.004	157	169	1	KNVTLNPDPNEIK
Isopentenyl-diphosphate delta isomerase	2319.083	2319.129	-0.047	114	133	0	AELGIPLEEVDLNEMDYLTR
Isopentenyl-diphosphate delta isomerase	2335.092	2335.124	-0.032	114	133	0	***AELGIPLEEVDLNEMDYLTR***
KIAA0445	941.561	941.54	0.021	913	920	1	EALGLRQR
KIAA0445	1184.701	1184.651	0.05	240	249	1	LQAAQEELKR
KIAA0445	1184.701	1184.687	0.014	976	985	1	SLELRLEAVR
KIAA0445	1397.832	1397.824	0.008	837	849	1	LALLEEARVSVAK
KIAA0445	1453.835	1453.836	-0.001	488	501	2	ERAGLAVKLAAAER
KIAA0445	1501.854	1501.814	0.04	870	880	3	***RELQELRRQMK***
KIAA0445	1521.882	1521.837	0.045	275	286	2	SHRQLEQLEVKR
KIAA0445	1521.882	1521.823	0.059	1552	1563	3	TLEARERAHRQR
KIAA0445	1557.877	1557.858	0.019	1220	1232	3	RRLKEVLDASESR
KIAA0445	1600.915	1600.864	0.051	794	807	3	SLSEGAKEREALRR
KIAA0445	1600.915	1600.864	0.051	793	806	3	RSLSEGAKEREALR
KIAA0445	1611.937	1611.839	0.098	694	707	1	HSLAAISLEMERQK
KIAA0445	1614.932	1614.868	0.064	719	732	1	NTLNALTSELRDLR
KIAA0445	1641.914	1641.843	0.071	453	466	1	QLSGRDQELEQALR
KIAA0445	1641.914	1641.89	0.023	201	214	2	EAQRLRSANELLSR
KIAA0445	1641.914	1641.854	0.06	467	480	2	ESQRQVEALERAAR
KIAA0445	1651.835	1651.863	-0.028	217	231	0	GNLTHSLQVTQQQAK

Protein Name	Measured Mass (Da)	Calculated Mass (Da)	Error	Peptide Start	Peptide End	Missed cleavage	Sequence
KIAA0445	1747.928	1747.896	0.032	1444	1457	3	RDLERSALQFDKDR
KIAA0445	1969.053	1969.047	0.006	828	844	2	LANEDKEQKLALLEEAR
KIAA0445	1972.045	1972.058	-0.013	1224	1240	2	EVLDASESRSIKLELQR
KIAA0445	2008.067	2008.117	-0.05	275	291	3	SHRQLEQLEVKRSGLTK
KIAA0445	2087.025	2087.085	-0.06	819	836	3	AESERISLKLANEDKEQK
KIAA0445	2272.104	2272.155	-0.051	1135	1154	3	AKQLQKAVAESEEAWRSADR
KIAA0635	964.488	964.501	-0.013	1063	1070	1	ETSMLRTK
KIAA0635	1267.637	1267.656	-0.019	353	362	0	LQWELDLSHK
KIAA0635	1740.801	1740.888	-0.087	191	204	0	IQELQEEVQQLQEK
KIAA0635	1790.827	1790.84	-0.013	868	881	1	***FQMLHSRAEDWEVK***
KIAA0635	1812.833	1812.816	0.017	339	352	2	***NLCEMRNLEEKMSK***
KIAA0635	1851.84	1851.925	-0.085	42	57	1	AEKESANLDFVLEPYK
KIAA0635	1875.862	1875.924	-0.062	677	692	1	GELESAQEQIKMLEQK
KIAA0635	1900.899	1900.957	-0.058	860	874	2	ENKDLLDRFQMLHSR
KIAA0635	1982.896	1982.958	-0.063	656	671	1	IVSEQLQRSLDDCQHR
KIAA0635	2086.991	2087.03	-0.039	297	313	1	NEKLCQELTEIDQLAQR
KIAA0635	2161.996	2161.983	0.013	473	490	2	CSEKGDCSTDVHLITRER
KIAA0635	2272.089	2272.056	0.033	693	711	1	***LENLSHRMTVQSEETHAMK***
KIAA0635	2311.063	2311.084	-0.021	700	719	2	***MTVQSEETHAMKKTIGVMDK***
KIAA0635	2326.04	2326.121	-0.081	1071	1089	2	***VTQLQTDYDNLKRQMSNEK***
KIAA1141 protein	985.518	985.545	-0.027	392	399	1	AHAQRLYK
KIAA1141 protein	1655.743	1655.831	-0.088	755	767	2	TFHQRSCLSKHQK
KIAA1141 protein	1707.746	1707.77	-0.024	824	838	0	IHTGEKPYTCGTCGK
KIAA1141 protein	1740.802	1740.861	-0.059	556	569	1	HNKIHTIEGLYECK
KIAA1141 protein	1774.781	1774.871	-0.09	361	374	3	HQKTPTNAKCFRCK
KIAA1141 protein	1787.783	1787.848	-0.065	537	550	2	HFGCAKCKETFIYK
KIAA1141 protein	1793.746	1793.739	0.007	418	432	1	KSHFPSAACECQGCR
KIAA1141 protein	1793.746	1793.739	0.007	419	433	1	SHFPSAACECQGCRK
KIAA1141 protein	1804.784	1804.822	-0.038	796	810	0	IHTGEKPYVCQECGK
KIAA1141 protein	1839.828	1839.875	-0.047	373	388	2	CKKCGETFSGAFHLAK
KIAA1141 protein	1844.776	1844.873	-0.097	376	391	1	CGETFSGAFHLAKHQR
KIAA1141 protein	1972.869	1972.968	-0.099	375	391	2	KCGETFSGAFHLAKHQR
KIAA1486 protein	1900.906	1900.884	0.022	150	168	0	QPPGACPSSLPSHGSSHAK
KIAA1486 protein	1936.989	1936.959	0.03	241	259	0	SSSSVPHTTPRPVSQDGAK
KIAA1486 protein	1968.995	1968.943	0.052	260	278	1	MVNAAVNTYSAAQSGSRSR
KIAA1486 protein	1985.001	1984.938	0.063	260	278	1	***MVNAAVNTYSAAQSGSRSR***
KIAA1610 protein	1654.78	1654.715	0.065	361	373	0	***SVGECVAFYYMWK***
KIAA1610 protein	1782.813	1782.81	0.003	361	374	1	***SVGECVAFYYMWKK***
KIAA1610 protein	1821.807	1821.816	-0.009	529	545	0	ESPGSSEFFQEAVSHGK
KIAA1610 protein	1835.84	1835.898	-0.058	390	404	2	KKYNLHPGVTDYMDR
KIAA1610 protein	1851.846	1851.893	-0.047	390	404	2	***KKYNLHPGVTDYMDR***
KIAA1610 protein	1878.913	1878.901	0.012	375	389	2	SERYDFFAQQTRFGK
KIAA1610 protein	1895.925	1895.869	0.056	359	373	1	TRSVGECVAFYYMWK
KIAA1610 protein	2006.978	2006.996	-0.018	375	390	3	SERYDFFAQQTRFGKK
KIAA1610 protein	2006.978	2006.996	-0.018	374	389	3	KSERYDFFAQQTRFGK
KIAA1610 protein	2037.918	2037.968	-0.05	223	240	0	***EIMVGSMFQAEIPVGVCR***
KIAA1610 protein	2053.891	2053.962	-0.071	223	240	0	***EIMVGSMFQAEIPVGVCR***
kinesin heavy chain	1080.663	1080.581	0.082	345	352	3	KYEKEKEK
kinesin heavy chain	1121.677	1121.538	0.139	881	890	2	***EAKEGAMKDK***
kinesin heavy chain	1333.793	1333.71	0.083	892	901	2	RYQQEVDRIK
kinesin heavy chain	1346.846	1346.745	0.101	596	606	3	SEVKSVVKRCR

Protein Name	Measured Mass (Da)	Calculated Mass (Da)	Error	Peptide Start	Peptide End	Missed cleavage	Sequence
kinesin heavy chain	1398.826	1398.812	0.014	545	556	2	KRIAEVLNGLMR
kinesin heavy chain	1432.825	1432.84	-0.015	800	811	2	LFVQDVTTRVKK
kinesin heavy chain	1432.825	1432.84	-0.015	799	810	2	KLFVQDVTTRVK
kinesin heavy chain	1474.793	1474.73	0.063	287	299	1	ILQDSLGGNCRSR
kinesin heavy chain	1489.817	1489.811	0.006	891	901	3	RRYQQEVDRIK
kinesin heavy chain	1489.817	1489.745	0.072	275	286	2	THVPYRDSKMTR
kinesin heavy chain	1496.803	1496.678	0.124	192	204	0	***HVAVTNMNEHSSR***
kinesin heavy chain	1520.817	1520.844	-0.027	228	241	1	LYLVDLAGSEKVSK
kinesin heavy chain	1628.796	1628.836	-0.04	242	257	1	TGAEGAVLDEAKNINK
kinesin heavy chain	1628.796	1628.836	-0.04	509	522	0	SQQNQLLVDELSQK
kinesin heavy chain	1656.78	1656.894	-0.114	15	28	1	FRPLNEAEILRGDK
kinesin heavy chain	1740.804	1740.907	-0.103	642	655	2	***SLTEYMQTVELKKR***
kinesin heavy chain	1740.804	1740.814	-0.01	605	617	1	CRQLENLQVECHR
kinesin heavy chain	1788.846	1788.959	-0.113	892	905	3	RYQQEVDRIKEAVR
kinesin heavy chain	1788.846	1788.9	-0.054	409	422	2	IAPEERQKYEEEIR
kinesin heavy chain	1837.857	1837.996	-0.139	640	654	2	IRSLTEYMQTVELKK
kinesin heavy chain	1837.857	1837.859	-0.003	189	204	1	***ANRHVAVTNMNEHSSR***
kinesin heavy chain	1965.948	1965.935	0.013	281	297	2	***DSKMTRILQDSLGGNCR***
kinesin heavy chain	1972.911	1972.878	0.033	812	829	1	***SAEMEPEDSGGIHSQKQK***
LaXp180 protein	1334.663	1334.649	0.014	952	962	1	***QSREMALEDLK***
LaXp180 protein	1334.663	1334.682	-0.02	249	260	0	TTNTSLVTSFHK
LaXp180 protein	1372.726	1372.81	-0.085	1453	1463	1	***IMLLERTLQLK***
LaXp180 protein	1435.774	1435.74	0.034	655	668	0	IESTTGITTTTSPK
LaXp180 protein	1656.779	1656.876	-0.097	202	214	1	IPLLECLTRHSYR
LaXp180 protein	1688.791	1688.876	-0.085	949	962	2	ELKQSREMALEDLK
LaXp180 protein	1688.791	1688.858	-0.067	1002	1015	2	***VMTDHNMSLEKLKK***
LaXp180 protein	1713.761	1713.86	-0.099	955	968	2	***EMALEDLKKLHDEK***
LaXp180 protein	1728.823	1728.834	-0.011	1021	1035	0	IDQMLESHASTIQEK
LaXp180 protein	1764.818	1764.824	-0.006	553	567	0	GLDSWPSSFCTQKPR
LaXp180 protein	1791.811	1791.925	-0.115	1470	1485	1	LNQRLMSQSLSSVSSR
LaXp180 protein	1827.802	1827.936	-0.134	338	352	0	IIQPFMLECHQTIAK
LaXp180 protein	1872.849	1872.896	-0.047	1107	1122	0	IHDNNESYQVGLSELR
LaXp180 protein	1894.858	1894.82	0.038	280	296	0	ESCQSTVQQEEASVDAK
LaXp180 protein	1894.858	1894.779	0.079	453	467	0	WCCFVMLHADQDGEK
LaXp180 protein	1977.898	1977.971	-0.073	84	101	1	***APAIPKATFSTENDMEIK***
LaXp180 protein	2005.915	2006.016	-0.101	1123	1139	1	ALMTIEKDQCISELISR
LaXp180 protein	2036.934	2037.024	-0.09	939	954	2	IMHTQHCEIKELKQSR
LaXp180 protein	2060.93	2061.01	-0.08	1278	1295	1	EDSSSLVAELQEKLQEEK
LaXp180 protein	2070.884	2070.989	-0.105	933	948	1	***LLEMEKIMHTQHCEIK***
leucine-rich repeats and WD repeat domain containing 1	1068.58	1068.484	0.096	383	391	0	AGFCCSVIR
leucine-rich repeats and WD repeat domain containing 1	1098.673	1098.65	0.023	13	22	2	GRPKTDKLGK
leucine-rich repeats and WD repeat domain containing 1	1108.666	1108.623	0.043	238	247	1	DPVTLPPSKR
leucine-rich repeats and WD repeat domain containing 1	1117.663	1117.57	0.093	364	373	0	***WNMLAAAGLR***
leucine-rich repeats and WD repeat domain containing 1	1199.737	1199.775	-0.038	112	121	2	VSFLLPKLRK
leucine-rich repeats and WD repeat domain containing 1	1221.789	1221.78	0.008	538	548	0	QSVLPVVILVR
leucine-rich repeats and WD repeat domain containing 1	1257.65	1257.676	-0.026	363	373	1	RWNMLAAAGLR
leucine-rich repeats and WD repeat domain containing 1	1395.787	1395.79	-0.003	1	12	0	***MAPLTPQLLLQR***
leucine-rich repeats and WD repeat domain containing 1	1512.771	1512.75	0.021	140	151	1	ELMDRVTAHWQK
leucine-rich repeats and WD repeat domain containing 1	1532.831	1532.77	0.061	383	395	2	AGFCCSVIRAHKK
leucine-rich repeats and WD repeat domain containing 1	1592.937	1592.842	0.095	212	226	1	VPVEHPQAAGASKFR
lipocortin I	1212.549	1212.525	0.024	167	177	0	DITSDTSGDFR

Protein Name	Measured Mass (Da)	Calculated Mass (Da)	Error	Peptide Start	Peptide End	Missed cleavage	Sequence
lipocortin I	1261.62	1261.593	0.026	114	124	0	TPAQFDADELR
lipocortin I	1340.669	1340.62	0.049	167	178	1	DITSDTSGDFRK
lipocortin I	1386.763	1386.76	0.004	59	71	0	GVDEATIIDILTK
lipocortin I	1522.797	1522.798	-0.001	215	228	0	GTDVNVFTTILTSR
lipocortin I	1639.785	1639.768	0.016	163	177	1	DLAKDITSDTSGDFR
lipocortin I	1650.844	1650.893	-0.049	214	228	1	KGTDVNVFTTILTSR
lipocortin I	1723.814	1723.841	-0.027	13	26	0	FLENQEQEYVQAVK
lipocortin I	1745.824	1745.904	-0.08	129	144	0	GLGTDEDTLIEILTTR
lipocortin I	1819.737	1819.877	-0.14	243	257	1	***YSQHDMNKALDLELK***
lipocortin I	1819.737	1819.8	-0.063	189	204	0	CQDLSVNQDLADTDAR
lipocortin I	2343.117	2343.148	-0.031	30	53	0	GGPGSAVSPYPSFNVSSDVAALHK
low density lipoprotein-related protein 12	945.56	945.487	0.072	758	766	0	QLDTAVSGR
low density lipoprotein-related protein 12	1208.602	1208.61	-0.008	229	238	0	VYTCLPESLK
low density lipoprotein-related protein 12	1438.793	1438.741	0.052	663	678	0	DTAGASGGVAAPLPQK
low density lipoprotein-related protein 12	2184.07	2184.153	-0.083	331	351	0	VLTAFDSHAPLTVVSSSGQIR
low density lipoprotein-related protein 12	2207.124	2207.085	0.039	82	101	0	ANPGEIITISFQDFDIQGSR
low density lipoprotein-related protein 12	2270.018	2270.076	-0.059	614	636	0	HSGSLALVSGDGDEVVPSQSSSR
L-xylulose reductase	1112.702	1112.637	0.065	199	208	1	AMLDRIPLGK
L-xylulose reductase	1154.765	1154.702	0.063	110	120	0	AVIQVSQIVAK
L-xylulose reductase	1410.779	1410.747	0.032	162	173	1	***MMALELGPHKIR***
L-xylulose reductase	1426.805	1426.742	0.063	162	173	1	***MMALELGPHKIR***
L-xylulose reductase	1584.779	1584.811	-0.032	174	188	0	VNAVNPTVVMTPMGR
L-xylulose reductase	1600.802	1600.806	-0.004	174	188	0	***VNAVNPTVVMTPMGR***
malate dehydrogenase	1177.611	1177.609	0.003	221	230	0	GEFITTVQQR
malate dehydrogenase	1370.741	1370.722	0.019	80	92	0	DLDVAVLVGSMPR
malate dehydrogenase	1386.741	1386.717	0.024	80	92	0	***DLDVAVLVGSMPR***
malate dehydrogenase	1392.725	1392.703	0.022	299	310	0	FVEGLPINDFSR
malate dehydrogenase	1432.728	1432.792	-0.064	202	214	1	LQGKEVGVYEALK
malate dehydrogenase	1750.848	1750.877	-0.029	206	220	1	EVGVYEALKDDSWLK
malate dehydrogenase	1756.858	1756.913	-0.056	126	142	0	VIVVGNPANTNCLTASK
malate dehydrogenase	1829.792	1829.856	-0.064	319	334	1	ELTEEKETAFEFLSSA
MAMA/CyCAP precursor	950.446	950.464	-0.018	105	112	0	***SLGWMVSR***
MAMA/CyCAP precursor	1730.86	1730.808	0.052	547	562	0	TPSLFPCASGAFSSFR
MAMA/CyCAP precursor	1773.84	1773.798	0.042	230	245	0	LASAYGATELQDYCGR
MAMA/CyCAP precursor	1990.957	1990.945	0.012	408	424	0	***LYTSSTWSSLVMASTWR***
matrix metalloproteinase 12	1190.518	1190.563	-0.046	400	409	0	QELMDPAYPK
matrix metalloproteinase 12	1257.624	1257.66	-0.036	360	371	0	SIYSLGFSASVK
matrix metalloproteinase 12	1336.642	1336.786	-0.144	410	421	0	LISTHFPGIKPK
matrix metalloproteinase 12	1417.704	1417.735	-0.031	130	141	0	AFQVWSDVTPLR
matrix metalloproteinase 12	1558.714	1558.772	-0.058	385	395	1	VYFFVDKHYWR
matrix metalloproteinase 12	1720.806	1720.904	-0.098	130	143	1	AFQVWSDVTPLRFR
matrix metalloproteinase 12	1723.788	1723.823	-0.035	396	409	1	YDVRQELMDPAYPK
matrix metalloproteinase 12	1739.739	1739.818	-0.079	396	409	1	***YDVRQELMDPAYPK***
matrix metalloproteinase 12	1761.772	1761.907	-0.135	291	302	1	ILFFKDWFFWWK
matrix metalloproteinase 12	2007.939	2007.987	-0.048	227	242	1	SIMYPTYRYLNPSTFR
Microtubule-associated protein 7 domain containing 1	1073.545	1073.553	-0.008	253	262	0	CSVSAVNLPK
Microtubule-associated protein 7 domain containing 1	1073.545	1073.601	-0.056	717	724	2	RLEEIMKR
Microtubule-associated protein 7 domain containing 1	1177.62	1177.645	-0.025	149	158	2	EERAKYLAAK
Microtubule-associated protein 7 domain containing 1	1184.62	1184.651	-0.031	753	763	0	AVETRPSGLQK
Microtubule-associated protein 7 domain containing 1	1190.616	1190.643	-0.028	718	726	2	***LEEIMKRTR***
Microtubule-associated protein 7 domain containing 1	1203.624	1203.614	0.01	636	644	2	***RMREEQLAR***

Protein Name	Measured Mass (Da)	Calculated Mass (Da)	Error	Peptide Start	Peptide End	Missed cleavage	Sequence
Microtubule-associated protein 7 domain containing 1	1216.638	1216.692	-0.054	220	229	2	SVKKTWAEIR
Microtubule-associated protein 7 domain containing 1	1229.63	1229.578	0.052	703	711	1	HFQKEEQER
Microtubule-associated protein 7 domain containing 1	1232.628	1232.661	-0.033	453	464	1	LSTGSELSPKSK
Microtubule-associated protein 7 domain containing 1	1248.658	1248.689	-0.031	302	312	0	LMTPTLSFLAR
Microtubule-associated protein 7 domain containing 1	1273.661	1273.662	-0.001	425	435	2	ENEKEKSALAR
Microtubule-associated protein 7 domain containing 1	1312.658	1312.634	0.024	322	334	2	NGRDQGRGSGPGR
Microtubule-associated protein 7 domain containing 1	1312.658	1312.732	-0.074	313	324	2	SRSAVTLPRNGR
Microtubule-associated protein 7 domain containing 1	1329.671	1329.729	-0.057	160	170	2	AVWLEKEEKAK
Microtubule-associated protein 7 domain containing 1	1329.671	1329.638	0.033	691	700	2	EEAERQRQER
Mitogen-activated protein kinase binding protein 1	1219.556	1219.644	-0.088	93	104	0	TITALAFSPDGK
Mitogen-activated protein kinase binding protein 1	1284.657	1284.584	0.073	1167	1177	0	HNNLDNSWASK
Mitogen-activated protein kinase binding protein 1	1426.732	1426.748	-0.016	500	511	0	IHELQSLSEMLK
Mitogen-activated protein kinase binding protein 1	1835.933	1835.937	-0.004	1308	1325	0	GLAHNETEQSGPLVSLGK
Mitogen-activated protein kinase binding protein 1	2184.055	2184.031	0.024	785	804	0	EGEDEGTEEEELPALPILSK
Mitogen-activated protein kinase binding protein 1	1110.569	1110.603	-0.034	870	879	0	QIETLAPSPR
Mitogen-activated protein kinase binding protein 1	1199.59	1199.65	-0.061	809	820	0	ELASGSSPALLR
MLL3-like protein	1173.606	1173.531	0.075	270	278	1	AQCMFFKDK
MLL3-like protein	1208.608	1208.619	-0.011	353	363	0	ALFPVGYEASR
MLL3-like protein	1312.668	1312.669	-0.001	39	50	0	ESLPSLPQSPMK
MLL3-like protein	1677.806	1677.793	0.013	591	603	1	***LYESQNRGVYMFR***
MLL3-like protein	1698.798	1698.876	-0.078	411	424	1	DVWDKILEPVACVR
MLL3-like protein	1724.856	1724.923	-0.068	116	130	1	TVPVGLEDCRPLNKK
MLL3-like protein	1769.868	1769.886	-0.018	39	54	1	***ESLPSLPQSPMKEPSK***
MLL3-like protein	1776.841	1776.76	0.081	241	255	1	CVFCHKTGATSGCHR
MLL3-like protein	1797.857	1797.831	0.026	604	620	0	***MDNDHVIDATLTGGPAR***
MLL3-like protein	1817.873	1817.833	0.04	256	269	1	FRCTNIYHFTCATK
MLL3-like protein	1851.9	1851.92	-0.021	394	410	0	IVEQGHEDLVLSDSSPK
MLL3-like protein	1851.9	1851.932	-0.032	473	489	0	NPLMELPLAVNPTGCAR
MLL3-like protein	1923.939	1923.891	0.048	148	163	0	GTFKPPCEDEIDEFLK
MLL3-like protein	1954.923	1954.908	0.015	453	469	0	IAESLPGVEACENYTFR
multidrug resistance-associated protein 7A	971.572	971.514	0.058	1347	1355	0	GQNLSLGQR
multidrug resistance-associated protein 7A	985.538	985.566	-0.029	526	533	1	VSLDRIQR
multidrug resistance-associated protein 7A	1184.704	1184.64	0.064	1425	1435	0	VVELDSPSALR
multidrug resistance-associated protein 7A	1199.735	1199.702	0.033	576	585	1	TFISHLQVKK
multidrug resistance-associated protein 7A	1252.792	1252.713	0.079	1238	1249	1	VEPGEKLGIVGR
multidrug resistance-associated protein 7A	1369.882	1369.782	0.099	1396	1407	1	FANKTVLTIAHR
multidrug resistance-associated protein 7A	1471.919	1471.879	0.04	421	433	1	LMTELLSGIRVIK
multidrug resistance-associated protein 7A	1512.937	1512.833	0.104	751	764	0	ADVVLLMEAGQLVR
multidrug resistance-associated protein 7A	1585.01	1584.901	0.109	1356	1369	1	QLLCLARALLTDAK
multidrug resistance-associated protein 7A	1649.955	1649.809	0.146	1	13	0	***MICGLLFFSFFPR***
multidrug resistance-associated protein 7A	1868.11	1867.993	0.117	595	612	1	VGCGKSSLLAAITGELHR
Myelin transcription factor 1 like	1431.751	1431.637	0.114	390	401	1	QEDDFPGRTPDR
Myelin transcription factor 1 like	1445.779	1445.827	-0.048	1065	1077	2	KAKLSGEQMLTIK
Myelin transcription factor 1 like	1784.833	1784.89	-0.057	964	978	2	IAQSKEDKEDQEPIR
Myelin transcription factor 1 like	1812.839	1812.89	-0.051	1009	1023	2	RQKDGYLNGSQFSWK
Myelin transcription factor 1 like	1875.889	1875.987	-0.098	559	574	1	***DRVPPEILAMHENVLK***
Myelin transcription factor 1 like	1885.92	1886.028	-0.108	902	918	2	KDLITLSGCPLADKSIR
Myelin transcription factor 1 like	1970.919	1971.045	-0.126	595	613	2	SLSGCPIAAAEKLAKAQEK
Myelin transcription factor 1 like	1970.919	1970.984	-0.065	1010	1026	2	QKDGYLNGSQFSWKSVK
Myelin transcription factor 1 like	1979.884	1979.969	-0.085	507	523	2	SKSSDSHVKKPYYDPSR
Myelin transcription factor 1 like	2087.999	2088.07	-0.071	881	898	1	YPGEVTIPSPKPKYPQCK

Protein Name	Measured Mass (Da)	Calculated Mass (Da)	Error	Peptide Start	Peptide End	Missed cleavage	Sequence
Myelin transcription factor 1 like	2146.901	2146.951	-0.05	323	340	0	***NMNYVMLGKPMNNGLMEK***
Myelin transcription factor 1 like	2198.981	2199.069	-0.088	367	385	2	***KLSETNPQDRSQPPNMSVR***
Myelin transcription factor 1 like	2198.981	2199.011	-0.03	402	419	1	SYSDMMNLMRLEEQLSPR
Myelin transcription factor 1 like	2358.971	2359.05	-0.079	666	684	2	YSKTSFEYNSYDNHTYGKR
Myelin transcription factor 1 like	2449.028	2449.11	-0.082	972	993	1	EDQEPIRCPVPGCDGQGHITGK
Myelin transcription factor 1 like	2575.14	2575.269	-0.13	561	583	1	VPPEILAMHENVLKCPTPGCTGR
Myelin transcription factor 1 like	2575.14	2575.266	-0.127	368	389	2	LSETNPQDRSQPPNMSVRQHVR
Myelin transcription factor 1 like	2668.161	2668.24	-0.079	398	419	2	TPDRSYSDMMNLMRLEEQLSPR
Myelin transcription factor 1 like	2692.193	2692.13	0.063	390	411	2	***QEDDFPGRTPDRSYSDMMNLMR***
Myelin transcription factor 1 like	2708.13	2708.125	0.004	390	411	2	***QEDDFPGRTPDRSYSDMMNLMR***
Myelin transcription factor 1 like	2714.315	2714.449	-0.134	1	26	2	RELALGLHPAHGVTLPPSSGEKASYK
Myelin transcription factor 1 like	2852.335	2852.446	-0.111	447	471	2	***SEEVFDMTKGNLTLLEKAIALETER***
Myosin, heavy polypeptide 2, skeletal muscle	957.51	957.56	-0.05	1060	1067	2	RKLEGDLK
Myosin, heavy polypeptide 2, skeletal muscle	973.481	973.49	-0.009	679	686	0	CIIPNETK
Myosin, heavy polypeptide 2, skeletal muscle	975.466	975.487	-0.021	868	876	1	DDLAKSEAK
Myosin, heavy polypeptide 2, skeletal muscle	994.546	994.471	0.074	1382	1389	0	YETDAIQR
Myosin, heavy polypeptide 2, skeletal muscle	1014.529	1014.586	-0.057	1459	1466	1	ILAEWKQK
Myosin, heavy polypeptide 2, skeletal muscle	1014.529	1014.582	-0.053	1362	1370	1	AELQRALSK
Myosin, heavy polypeptide 2, skeletal muscle	1149.65	1149.669	-0.019	1589	1598	1	KPRKPSLMPP
Myosin, heavy polypeptide 2, skeletal muscle	1156.657	1156.656	0.001	1081	1089	2	QQLDERLKK
Myosin, heavy polypeptide 2, skeletal muscle	1193.635	1193.597	0.038	1174	1182	2	REAEFQKMR
Myosin, heavy polypeptide 2, skeletal muscle	1193.635	1193.597	0.038	1175	1183	2	EAEFQKMRR
Myosin, heavy polypeptide 2, skeletal muscle	1287.615	1287.657	-0.042	25	35	0	IEAQNRPFDAK
Myosin, heavy polypeptide 2, skeletal muscle	1311.64	1311.678	-0.038	971	981	2	VEKEKHATENK
Myosin, heavy polypeptide 2, skeletal muscle	1445.836	1445.815	0.021	878	889	2	KELEEKMVSLLK
Myosin, heavy polypeptide 2, skeletal muscle	1445.836	1445.74	0.096	1442	1454	2	TNAACAALDKKQR
Myosin, heavy polypeptide 2, skeletal muscle	1501.784	1501.777	0.007	752	764	0	LLGSIDIDHTQYK
Myosin, heavy polypeptide 2, skeletal muscle	1518.795	1518.723	0.072	1068	1080	0	LAQESIMDIENEK
Myosin, heavy polypeptide 2, skeletal muscle	1518.795	1518.803	-0.008	1311	1323	1	GKQAFTQQIEELK
Myosin, heavy polypeptide 2, skeletal muscle	1518.795	1518.819	-0.024	1455	1466	2	NFDKILAEWKQK
Myosin, heavy polypeptide 2, skeletal muscle	1729.869	1729.892	-0.023	687	701	0	TPGAMEHELVLHQLR
Myosin, heavy polypeptide 2, skeletal muscle	2288.125	2288.155	-0.03	815	832	2	RESIFCIQYNIRAFMNVK
Myosin, heavy polypeptide 2, skeletal muscle	2288.125	2288.094	0.031	1068	1086	1	LAQESIMDIENEKQQLDER
Myosin-Va	1080.689	1080.611	0.078	710	717	2	***YRVLMKQK***
Myosin-Va	1084.648	1084.569	0.079	920	928	1	***KLHIGMENK***
Myosin-Va	1092.681	1092.586	0.095	232	240	1	YRIIGANMR
Myosin-Va	1094.688	1094.681	0.007	1488	1496	0	NLILELKPR
Myosin-Va	1116.691	1116.661	0.03	814	823	2	RTKAATTIQK
Myosin-Va	1121.665	1121.604	0.061	899	907	3	***MMAKRDVKK***
Myosin-Va	1128.727	1128.629	0.098	401	410	1	DALAKHIYAK
Myosin-Va	1128.727	1128.638	0.089	985	994	0	VLSLQEEIAK
Myosin-Va	1143.693	1143.624	0.069	1479	1487	2	REDEQKLVK
Myosin-Va	1244.741	1244.643	0.098	1810	1820	1	KDSPQLLMDAK
Myosin-Va	1295.78	1295.683	0.097	1370	1380	0	IEASLQHEITR
Myosin-Va	1331.798	1331.745	0.053	1801	1810	3	***TIQMRLRDRK***
Myosin-Va	1349.765	1349.682	0.083	568	578	1	NKDTVFEEQIK
Myosin-Va	1454.868	1454.808	0.059	1271	1283	1	SQLVSQKEAIQPK
Myosin-Va	1472.797	1472.7	0.097	1517	1528	1	HADYLNDDQKVR
Myosin-Va	1490.921	1490.856	0.065	908	919	3	LKIEARSVERYK
Myosin-Va	1520.794	1520.838	-0.044	234	246	1	IIGANMRTYLLEK
Myosin-Va	1544.846	1544.87	-0.024	712	724	3	***VLMKQKDVLGDRK***

Protein Name	Measured Mass (Da)	Calculated Mass (Da)	Error	Peptide Start	Peptide End	Missed cleavage	Sequence
Myosin-Va	1546.915	1546.878	0.036	1148	1160	1	***VPLDMSLFLKLQK***
Myosin-Va	1558.765	1558.744	0.021	1468	1479	2	***EKDFQGMLEYKR***
Myosin-Va	1558.765	1558.802	-0.037	196	210	0	***VLASNPIMESIGNAK***
Myosin-Va	1624.863	1624.817	0.046	786	798	1	***YLCMQRAAITVQR***
Myosin-Va	1641.816	1641.742	0.074	1706	1718	2	***KDMCSWSKGMQIR***
Myosin-Va	1654.866	1654.79	0.075	585	597	1	***FKMLPELFQDDEK***
Myosin-Va	1686.785	1686.839	-0.054	1467	1479	3	***KEKDFQGMLEYKR***
Myosin-Va	1704.816	1704.814	0.002	885	897	1	***TMKAIVYLQCCFR***
nasopharyngeal epithelium specific protein 1	965.486	965.42	0.066	442	448	1	DRDEFER
nasopharyngeal epithelium specific protein 1	967.515	967.443	0.072	534	540	0	YCIEVER
nasopharyngeal epithelium specific protein 1	975.468	975.542	-0.074	309	316	1	***LKMQAEIK***
nasopharyngeal epithelium specific protein 1	1153.649	1153.543	0.106	284	292	0	***EQMLAYLDR***
nasopharyngeal epithelium specific protein 1	1155.645	1155.635	0.01	173	182	1	AQNLLQRADK
nasopharyngeal epithelium specific protein 1	1163.626	1163.539	0.087	153	161	1	EMTWNNNKK
nasopharyngeal epithelium specific protein 1	1185.661	1185.621	0.04	391	400	2	AKRNQEVADR
nasopharyngeal epithelium specific protein 1	1185.661	1185.682	-0.021	511	519	3	RERIEDIKK
nasopharyngeal epithelium specific protein 1	1208.659	1208.64	0.019	50	60	0	SDSPIVVIHDK
nasopharyngeal epithelium specific protein 1	1218.595	1218.602	-0.007	325	334	1	***QKAEMLAQER***
nasopharyngeal epithelium specific protein 1	1244.701	1244.672	0.029	460	469	3	EKQEQEKKAK
nasopharyngeal epithelium specific protein 1	1307.693	1307.592	0.101	151	160	1	***QKEMTWNNNK***
nasopharyngeal epithelium specific protein 1	1307.693	1307.749	-0.056	197	207	1	IILNAKCHAIR
nasopharyngeal epithelium specific protein 1	1325.772	1325.777	-0.005	368	378	2	EIARLRALQEK
nasopharyngeal epithelium specific protein 1	1334.743	1334.814	-0.071	257	267	3	GKRHIVEQIKK
nasopharyngeal epithelium specific protein 1	1343.737	1343.609	0.128	335	345	0	***LADQMVMEFTK***
nasopharyngeal epithelium specific protein 1	1424.719	1424.821	-0.102	173	184	2	AQNLLQRADKLR
nasopharyngeal epithelium specific protein 1	1427.79	1427.772	0.017	122	133	2	EELNAREQALKK
nasopharyngeal epithelium specific protein 1	1431.79	1431.758	0.032	444	454	2	DEFERILRAQR
nasopharyngeal epithelium specific protein 1	1445.883	1445.735	0.148	408	419	2	ENAQKKIETEEK
nasopharyngeal epithelium specific protein 1	1582.877	1582.915	-0.038	366	378	3	EKEIARLRALQEK
nasopharyngeal epithelium specific protein 1	1656.816	1656.844	-0.028	391	403	3	AKRNQEVADREWR
nasopharyngeal epithelium specific protein 1	1702.982	1702.873	0.109	406	419	3	EKENAQKKIETEEK
nasopharyngeal epithelium specific protein 1	1702.982	1702.885	0.096	442	454	3	DRDEFERILRAQR
nasopharyngeal epithelium specific protein 1	2272.092	2272.052	0.04	327	345	1	***AEMLAQERLADQMVMEFTK***
nasopharyngeal epithelium specific protein 1	2288.103	2288.048	0.055	327	345	1	***AEMLAQERLADQMVMEFTK***
Ncapg2 protein	1128.544	1128.584	-0.04	517	527	0	DVVMVGLGDPK
Ncapg2 protein	1149.481	1149.563	-0.082	171	179	0	***CVTLIQMNR***
Ncapg2 protein	1170.559	1170.538	0.021	207	215	0	HCLNACIQR
Ncapg2 protein	1260.605	1260.62	-0.015	128	137	0	***ICPMEDILVR***
Ncapg2 protein	1578.717	1578.788	-0.071	54	68	0	VTGELAFDISSADVR
N-deacetylase/N-sulfotransferase 4	1098.579	1098.555	0.024	115	125	0	GDIPPLTDSGK
N-deacetylase/N-sulfotransferase 4	1199.624	1199.586	0.038	560	568	0	YFELFPEQK
N-deacetylase/N-sulfotransferase 4	1381.821	1381.785	0.036	128	138	0	YTLIIYENILK
N-deacetylase/N-sulfotransferase 4	1445.856	1445.802	0.053	179	191	0	GFPLNLFNNVALK
N-deacetylase/N-sulfotransferase 4	1754.926	1754.903	0.023	740	753	1	RCLVPGWYAVHIER
N-deacetylase/N-sulfotransferase 4	1768.888	1768.845	0.043	139	152	1	YVSMDSWNRELLEK
N-deacetylase/N-sulfotransferase 4	1957.982	1957.953	0.029	836	851	1	TFLSSYYRDHNVELSK
nebulin	1428.771	1428.724	0.047	63	76	0	GLGWSPAGSLEVEK
nebulin	1431.692	1431.714	-0.022	120	130	1	HLYTIDWNKDK
nebulin	1474.776	1474.639	0.137	773	784	0	QAYDLQSDNMYK
nebulin	1474.776	1474.712	0.064	876	887	1	MNKVNYSESLYK
nebulin	1676.804	1676.88	-0.076	29	42	0	***YILLPDAMNIELTR***

Protein Name	Measured Mass (Da)	Calculated Mass (Da)	Error	Peptide Start	Peptide End	Missed cleavage	Sequence
nebulin	1706.802	1706.927	-0.125	1212	1227	1	TKYSSPVDMLGVVLAK
nebulin	1706.802	1706.927	-0.125	483	498	1	TKYSSPVDMLGVVLAK
nebulin	1706.802	1706.927	-0.125	969	984	1	TKYSSPVDMLGVVLAK
nebulin	1706.802	1706.927	-0.125	726	741	1	TKYSSPVDMLGVVLAK
nebulin	1734.821	1734.8	0.021	702	715	1	***MMWSMHVAKIQSDR***
nebulin	1734.821	1734.8	0.021	1188	1201	1	***MMWSMHVAKIQSDR***
nebulin	1734.821	1734.8	0.021	945	958	1	***MMWSMHVAKIQSDR***
nebulin	1734.821	1734.8	0.021	459	472	1	***MMWSMHVAKIQSDR***
nebulin	1767.819	1767.852	-0.033	1344	1358	1	DKTQIHIMPDTPEIM
nebulin	1798.863	1798.906	-0.044	1172	1187	1	QLGHHIGARNIEDDPK
nebulin	1846.877	1846.902	-0.026	582	597	1	***FKFSSLMDSMPMVLAK***
nebulin	1864.846	1864.953	-0.107	860	875	0	TQIHIMPDTPEIMLAR
nebulin	1931.922	1932.031	-0.109	168	185	1	GYDLPVDAISVQLAKTSR
nebulin	2105.996	2106.095	-0.1	1101	1118	1	DKIQIHVMPDTPEIMLAR
nebulin	2238.03	2238.131	-0.101	860	878	1	TQIHIMPDTPEIMLARMNK
nebulin	2254.07	2254.126	-0.056	860	878	1	***TQIHIMPDTPEIMLARMNK***
nebulin	2318.132	2318.088	0.044	288	305	1	***VYELQSENMYKSDLEWLR***
nebulin	2452.11	2452.189	-0.079	1	23	1	***PADMLSVTAAKDAQANITNTNYK***
NF-kappa-B-repressing factor (NFkB-repressing factor)	945.443	945.449	-0.006	87	94	0	THFASMPR
NF-kappa-B-repressing factor (NFkB-repressing factor)	972.448	972.512	-0.064	667	674	0	EDLLDQLK
NF-kappa-B-repressing factor (NFkB-repressing factor)	974.381	974.503	-0.122	525	533	0	GTVEDVISR
NF-kappa-B-repressing factor (NFkB-repressing factor)	978.4	978.538	-0.138	223	231	0	EIPPADIPK
NF-kappa-B-repressing factor (NFkB-repressing factor)	978.4	978.459	-0.059	562	571	0	***MGWTGGGLGK***
NF-kappa-B-repressing factor (NFkB-repressing factor)	1173.59	1173.585	0.005	197	205	0	INYTYMLTR
NF-kappa-B-repressing factor (NFkB-repressing factor)	1307.679	1307.675	0.003	212	222	0	TNPEYIYAPLK
NF-kappa-B-repressing factor (NFkB-repressing factor)	1766.837	1766.858	-0.021	675	690	0	QEGQVGHYELVVPQAN
NG, NG dimethylarginine dimethylaminohydrolase 2	919.441	919.4	0.041	174	182	0	***GLCGMGGPR***
NG, NG dimethylarginine dimethylaminohydrolase 2	926.516	926.493	0.023	97	104	0	RPEVDGVR
NG, NG dimethylarginine dimethylaminohydrolase 2	1012.602	1012.602	0	105	113	1	KALQDLGLR
NG, NG dimethylarginine dimethylaminohydrolase 2	1054.578	1054.588	-0.01	97	105	1	RPEVDGVRK
NG, NG dimethylarginine dimethylaminohydrolase 2	1150.604	1150.62	-0.016	41	51	1	AQREHGVLGGK
NG, NG dimethylarginine dimethylaminohydrolase 2	1657.79	1657.842	-0.052	158	173	0	DFAVSTVPVSGSSHLR
Ngef protein	1092.553	1092.548	0.005	464	472	0	***TEQMISIQK***
Ngef protein	1201.614	1201.575	0.039	493	503	0	QGELQQMSGPK
Ngef protein	1208.597	1208.546	0.051	645	654	0	TEDGWIFGER
Ngef protein	1520.762	1520.809	-0.047	537	549	1	YQVFDSAPRGLLR
Ngef protein	1648.826	1648.79	0.036	446	459	1	***ELEMVVKACNEGVR***
Ngef protein	1712.847	1712.945	-0.098	385	399	1	AAFRELIAQLELDPK
Ngef protein	1741.862	1741.918	-0.056	489	503	1	WLLKQGELQQMSGPK
Ngef protein	1750.9	1750.924	-0.024	168	181	0	NLIEQIGLLYQEYR
Ngef protein	1750.9	1750.823	0.077	661	675	0	***GWFPSSMTEEILNPK***
Ngef protein	1796.861	1796.861	0	568	582	1	***LLENADDREATYMLK***
Ngef protein	1887.949	1887.9	0.049	23	39	0	***SNHGPAEMRPALPPENR***
NmrA-like family domain containing 1	1289.732	1289.709	0.023	23	33	1	ALLEDGTFRIR
NmrA-like family domain containing 1	1587.889	1587.873	0.016	6	22	0	LVVVFGATGAQGGSVAR
NmrA-like family domain containing 1	1715.978	1715.968	0.01	5	22	1	KLVVVFGATGAQGGSVAR
NmrA-like family domain containing 1	1731.934	1731.93	0.004	114	128	0	LGLHYVVYSGLENIR
NmrA-like family domain containing 1	1923.969	1923.911	0.058	135	151	1	LAAGHFDGKGEVEEYFR
NmrA-like family domain containing 1	2127.066	2127.038	0.028	292	309	1	AQTLDQWLEQHKGDFAQL
NmrA-like family domain containing 1	2546.191	2546.174	0.017	251	272	1	***TTPEDYEKLGFQGAQDLANMFR***
Nrxn3 protein	1600.75	1600.774	-0.024	353	365	1	FNDNAWHDVKVTR

Protein Name	Measured Mass (Da)	Calculated Mass (Da)	Error	Peptide Start	Peptide End	Missed cleavage	Sequence
Nrxn3 protein	1625.745	1625.694	0.05	698	711	0	***EASILSYDGSMYMK***
Nrxn3 protein	1641.787	1641.861	-0.074	638	652	1	NIRQLAEMQNAAGVK
Nrxn3 protein	1700.848	1700.876	-0.028	1274	1288	0	LFQGQLSGLYYDGLK
Nrxn3 protein	1704.799	1704.804	-0.005	103	116	0	QVNDSSWHFLMVSR
Nrxn3 protein	1799.902	1799.934	-0.032	729	744	1	FMSQRAYGLLVATTSR
Nrxn3 protein	1816.828	1816.913	-0.085	824	838	1	LEFHNIETGIMTEKR
Nrxn3 protein	1951.929	1951.927	0.002	761	776	1	***LMVNLDCIRINCNSSK***
Nrxn3 protein	1975.939	1975.932	0.007	103	118	1	QVNDSSWHFLMVSRDR
Nrxn3 protein	1990.891	1990.931	-0.04	712	728	0	VIMPMVMHTEAEDVSFR
ORF2	1199.598	1199.666	-0.068	586	595	0	SINVIHYINK
ORF2	1512.771	1512.76	0.011	598	610	1	DKNHMIISLDAEK
ORF2	1658.799	1658.771	0.028	1216	1228	1	EPRCPSTEEWIQK
ORF2	1769.874	1769.934	-0.06	596	610	2	***LKDKNHMIISLDAEK***
ORF2	1860.876	1860.846	0.03	1229	1242	0	MWYIYTMEYYSAIK
ORF2	1971.965	1972.034	-0.069	806	821	2	WKDLPCSWIGRTNIVK
ORF2	1973.963	1973.987	-0.024	598	614	2	DKNHMIISLDAEKAFDK
ORF2	1988.982	1988.941	0.041	1229	1243	1	MWYIYTMEYYSAIKK
ORF2	2004.961	2004.936	0.025	1229	1243	1	***MWYIYTMEYYSAIKK***
ORF2	2072.023	2072.001	0.022	402	419	1	NEKGDITTDPEEIQNTIR
ORF2	2087.021	2086.988	0.032	1213	1228	2	NWKEPRCPSTEEWIQK
ORF2	2127.034	2127.161	-0.127	224	241	1	LKNIEIVPCILSDHHALR
ORF2	2201.036	2201.15	-0.115	530	548	1	DPTKIENFRPISLMNIDAK
ORF2	2254.14	2254.238	-0.099	873	892	2	***TSGGITMPDLKLYYRAIVIK***
ORF2	2310.149	2310.246	-0.097	762	782	1	ETTPFSIATNNIKYLGVTLTK
ORF2	2417.141	2417.215	-0.074	141	162	0	***AHIAPHTIIVGDFNTPLSPMDR***
ORF2	2509.161	2509.288	-0.127	1249	1269	1	***FLAKWMDLESIILSEVTQSQR***
ornithine aminotransferase	1280.651	1280.63	0.021	170	180	0	IVFADGNFWGR
ornithine aminotransferase	1491.856	1491.818	0.038	427	439	1	ESVEIINKTILSF
ornithine aminotransferase	1491.856	1491.865	-0.009	414	426	1	LAPPLVIKEDEIR
ornithine aminotransferase	1588.834	1588.849	-0.015	359	372	1	***ELMKLPSDVVTSVR***
ornithine aminotransferase	1735.864	1735.879	-0.015	50	64	0	YGAHNYHPLPVALER
ornithine aminotransferase	1809.939	1809.973	-0.034	256	271	0	HQVLFIADEIQTGLAR
ornithine aminotransferase	1920.963	1920.995	-0.032	50	66	1	YGAHNYHPLPVALERGK
ornithine aminotransferase	2107.06	2107.088	-0.028	275	292	0	***WLAVDHENVRPDMVLLGK***
ornithine aminotransferase	2111.036	2111.016	0.02	32	49	2	KTEQGPPSSEYIFERESK
ornithine aminotransferase	2404.32	2404.356	-0.037	414	434	2	LAPPLVIKEDEIRESVEIINK
Oxalosuccinate decarboxylase	975.55	975.55	0.001	101	109	0	NILGGTVFR
Oxalosuccinate decarboxylase	1008.442	1008.441	0.001	133	140	0	HAYGDQYR
Oxalosuccinate decarboxylase	1153.54	1153.522	0.018	261	270	0	SEGGFIWACK
Oxalosuccinate decarboxylase	1233.627	1233.609	0.018	250	260	0	LIDDMVAQAMK
Oxalosuccinate decarboxylase	1340.684	1340.668	0.016	302	314	0	TVEAEAAHGTVTR
Oxalosuccinate decarboxylase	1397.711	1397.682	0.029	346	357	0	LDNNTELSFFAK
Oxalosuccinate decarboxylase	1596.811	1596.814	-0.003	344	357	1	AKLDNNTELSFFAK
Oxalosuccinate decarboxylase	1735.798	1735.786	0.012	5	20	0	IQGGSVVEMQGDEMTR
Oxalosuccinate decarboxylase	1751.776	1751.781	-0.004	5	20	0	***IQGGSVVEMQGDEMTR***
Oxalosuccinate decarboxylase	1796.779	1796.84	-0.06	188	203	0	SIEDFAHSSFQMALSK
Oxalosuccinate decarboxylase	1796.779	1796.891	-0.112	302	317	1	TVEAEAAHGTVTRHYR
Oxalosuccinate decarboxylase	1812.776	1812.834	-0.059	188	203	0	***SIEDFAHSSFQMALSK***
Oxalosuccinate decarboxylase	1863.815	1863.881	-0.066	4	20	1	KIQGGSVVEMQGDEMTR
Oxalosuccinate decarboxylase	1863.815	1863.911	-0.096	322	338	0	GQETSTNPIASIFAWSR
Oxalosuccinate decarboxylase	1879.783	1879.876	-0.093	4	20	1	***KIQGGSVVEMQGDEMTR***

Protein Name	Measured Mass (Da)	Calculated Mass (Da)	Error	Peptide Start	Peptide End	Missed cleavage	Sequence
Oxalosuccinate decarboxylase	1895.799	1895.871	-0.072	4	20	1	***KIQGGSVVEMQGDEMTR***
Oxalosuccinate decarboxylase	2017.974	2018.071	-0.097	141	159	1	ATDFVVPGPGKVEITYTPK
p66 mot1	972.472	972.517	-0.045	361	368	1	RTIAPCQK
p66 mot1	1488.881	1488.829	0.052	349	361	1	AQFEGIVTDLIKR
p66 mot1	1669.917	1669.91	0.007	203	218	1	QATKDAGQISGLNVLR
p66 mot1	1671.872	1671.89	-0.018	620	634	2	ALLARKDSETGENIR
p66 mot1	1693.839	1693.842	-0.003	188	202	0	NAVITVPAYFNDSQR
p66 mot1	1723.872	1723.864	0.008	108	122	1	QAVTNPNNTFYATKR
p66 mot1	1723.872	1723.864	0.008	107	121	1	RQAVTNPNNTFYATK
p66 mot1	2162.063	2162.103	-0.04	86	106	1	***TTPSVVAFTADGERLVGMPAK***
p66 mot1	2318.176	2318.204	-0.028	86	107	2	***TTPSVVAFTADGERLVGMPAKR***
p66 mot1	2389.127	2389.186	-0.059	77	99	1	VLENAEGARTTPSVVAFTADGER
PDZ domain containing RING finger 4	900.459	900.394	0.065	423	428	1	***REFMMR***
PDZ domain containing RING finger 4	954.518	954.502	0.016	252	259	1	EYTSTKVK
PDZ domain containing RING finger 4	976.552	976.537	0.015	226	233	1	***MINLTNKK***
PDZ domain containing RING finger 4	1111.683	1111.537	0.145	423	430	2	REFMMRSR
PDZ domain containing RING finger 4	1199.701	1199.578	0.123	248	257	1	QSTREYTSTK
PDZ domain containing RING finger 4	1208.71	1208.578	0.132	172	181	0	NYNTSLDVQR
PDZ domain containing RING finger 4	1426.798	1426.675	0.122	182	193	1	***GKLDDIMEHPEK***
PDZ domain containing RING finger 4	1426.798	1426.741	0.057	248	259	2	QSTREYTSTKVK
PDZ domain containing RING finger 4	1541.79	1541.769	0.021	424	435	2	***EFMMRSRLESLK***
PDZ domain containing RING finger 4	1656.77	1656.782	-0.012	234	247	1	***NLRSMMTAHQSPPR***
PDZ domain containing RING finger 4	1740.802	1740.834	-0.032	182	196	2	GKLDDIMEHPEKSDK
Peptidyl-prolyl cis-trans isomerase A (Cyclophilin A)	1133.573	1133.574	-0.001	155	164	1	KITISDCGQL
Peptidyl-prolyl cis-trans isomerase A (Cyclophilin A)	1309.588	1309.563	0.025	134	144	0	***EGMNIVEAMER***
Peptidyl-prolyl cis-trans isomerase A (Cyclophilin A)	1378.74	1378.749	-0.009	20	31	1	VSFELFADKVPK
Peptidyl-prolyl cis-trans isomerase A (Cyclophilin A)	1422.689	1422.71	-0.021	32	44	1	TAENFRALSTGEK
Peptidyl-prolyl cis-trans isomerase A (Cyclophilin A)	1536.749	1536.727	0.022	132	144	1	***VKEGMNIVEAMER***
Peptidyl-prolyl cis-trans isomerase A (Cyclophilin A)	1613.741	1613.732	0.009	56	69	0	***IIPGFMCQGGDFTR***
Peptidyl-prolyl cis-trans isomerase A (Cyclophilin A)	1830.891	1830.903	-0.012	77	91	1	SIYGEKFEDENFILK
Peptidylprolyl isomerase A	1133.573	1133.574	-0.001	155	164	1	KITISDCGQL
Peptidylprolyl isomerase A	1309.588	1309.563	0.025	134	144	0	***EGMNIVEAMER***
Peptidylprolyl isomerase A	1378.74	1378.749	-0.009	20	31	1	VSFELFADKVPK
Peptidylprolyl isomerase A	1422.689	1422.71	-0.021	32	44	1	TAENFRALSTGEK
Peptidylprolyl isomerase A	1536.749	1536.727	0.022	132	144	1	***VKEGMNIVEAMER***
Peptidylprolyl isomerase A	1613.741	1613.732	0.009	56	69	0	***IIPGFMCQGGDFTR***
Peptidylprolyl isomerase A	1830.891	1830.903	-0.012	77	91	1	SIYGEKFEDENFILK
Per3	1523.801	1523.74	0.061	467	479	0	***NVGQQLYIESMAR***
Per3	1526.742	1526.699	0.042	1007	1020	0	DEAPPGAAEESIWR
Per3	1588.807	1588.732	0.075	590	605	0	QETTGPSTDIEGGAAR
Per3	1613.722	1613.734	-0.012	50	62	0	***MSEELIMVVQEMK***
Per3	1737.828	1737.806	0.022	1025	1038	0	***TPECVLMTYQVPER***
Per3	1830.861	1830.817	0.044	197	211	0	ASQYECAPAKPFFCR
Peroxiredoxin-2	1595.786	1595.782	0.004	11	26	0	SAPDFTATAVVDGAFK
Peroxiredoxin-2	1705.903	1705.96	-0.057	93	109	0	EGGLGPLNIPLLADVTK
Peroxiredoxin-2	1833.996	1834.055	-0.059	92	109	1	KEGGLGPLNIPLLADVTK
Peroxiredoxin-2	1965.988	1966.004	-0.016	11	29	1	SAPDFTATAVVDGAFKEIK
Peroxiredoxin-2	2025.966	2026.011	-0.045	110	127	1	SLSQNYGVLKNDEGIAYR
peroxiredoxin-6	905.503	905.46	0.043	156	162	0	NFDEILR
peroxiredoxin-6	930.563	930.528	0.035	57	64	1	LAPEFAKR
peroxiredoxin-6	1148.622	1148.659	-0.037	133	142	1	VVFIFGPDKK

Protein Name	Measured Mass (Da)	Calculated Mass (Da)	Error	Peptide Start	Peptide End	Missed cleavage	Sequence
peroxiredoxin-6	1161.622	1161.639	-0.017	205	215	1	GVFTKELPSGK
peroxiredoxin-6	1190.644	1190.665	-0.021	145	155	0	LSILYPATTGR
peroxiredoxin-6	1269.702	1269.708	-0.005	98	108	1	LPFPIIDDKGR
peroxiredoxin-6	1394.669	1394.649	0.02	42	53	0	DFTPVCTTELGR
peroxiredoxin-6	2288.113	2288.109	0.004	1	22	0	***MPGGLLLGDEAPNFEANTTIGR***
Pgam1	1149.641	1149.661	-0.021	181	191	0	VLIAAHGNSLR
Pgam1	1311.63	1311.595	0.035	11	21	0	HGESAWNLENR
Pgam1	1338.679	1338.688	-0.01	101	113	1	AETAAKHGEAQVK
Pgam1	1682.843	1682.902	-0.059	163	176	0	ALPFWNEEIVPQIK
Pgam1	1934.926	1934.882	0.044	47	62	1	DAGYEFDICFTSVQKR
Pgam1	2130.05	2130.113	-0.063	223	240	0	***NLKPIKPMQFLGDEETVR***
Pgam1	2424.112	2424.146	-0.034	142	162	1	YADLTEDQLPSCESLKDTIAR
Pgam1	2432.1	2432.098	0.001	118	138	0	***SYDVPPPPMEPDHPFYSNISK***
Pgam1	2588.228	2588.199	0.029	117	138	1	***RSYDVPPPPMEPDHPFYSNISK***
Pgam1	2703.227	2703.226	0.001	118	140	1	***SYDVPPPPMEPDHPFYSNISKDR***
Pgam1	974.487	974.485	0.002	242	251	0	AMEAVAAQGK
Pgam1	1311.636	1311.595	0.041	11	21	0	HGESAWNLENR
Pgam1	1934.886	1934.882	0.004	47	62	1	DAGYEFDICFTSVQKR
Pgam1	2424.094	2424.146	-0.053	142	162	1	YADLTEDQLPSCESLKDTIAR
Pgam1	2432.063	2432.098	-0.036	118	138	0	***SYDVPPPPMEPDHPFYSNISK***
Pgam1	2580.194	2580.248	-0.054	141	162	2	RYADLTEDQLPSCESLKDTIAR
Pgam1	2588.158	2588.199	-0.042	117	138	1	***RSYDVPPPPMEPDHPFYSNISK***
Pgam1	2843.299	2843.333	-0.034	118	141	2	SYDVPPPPMEPDHPFYSNISKDRR
Pgam1	2843.299	2843.333	-0.034	117	140	2	RSYDVPPPPMEPDHPFYSNISKDR
Pgam1	2859.32	2859.328	-0.008	118	141	2	***SYDVPPPPMEPDHPFYSNISKDRR***
Pgam1	2859.32	2859.328	-0.008	117	140	2	***RSYDVPPPPMEPDHPFYSNISKDR***
Phosphatidylethanolamine-binding protein 1	936.502	936.491	0.011	180	187	0	LYEQLSGK
Phosphatidylethanolamine-binding protein 1	1559.844	1559.819	0.025	63	76	0	LYTLVLTDPDAPSR
Phosphatidylethanolamine-binding protein 1	2497.282	2497.262	0.02	40	62	0	VLTPTQVMNRPSSISWDGLDPGK
phospholipase C, eta 1	1307.651	1307.599	0.052	485	495	1	DKEDPDSFTVR
phospholipase C, eta 1	1348.7	1348.688	0.012	20	29	1	LFYLDEHRTR
phospholipase C, eta 1	1441.775	1441.77	0.005	195	206	0	DLYLLLLSYSDK
phospholipase C, eta 1	1445.742	1445.824	-0.082	346	357	1	ILFRDVVETINK
phospholipase C, eta 1	1454.69	1454.791	-0.101	922	934	0	SLQVRPVSMPVDK
phospholipase C, eta 1	1454.69	1454.741	-0.051	1512	1523	1	QDVNQCPRALVR
phospholipase C, eta 1	1457.718	1457.737	-0.019	51	62	1	VTEGRQSEIFHR
phospholipase C, eta 1	1470.694	1470.786	-0.092	922	934	0	***SLQVRPVSMPVDK***
phospholipase C, eta 1	1505.732	1505.845	-0.113	43	55	1	ILIDSIYKVTEGR
phospholipase C, eta 1	1541.724	1541.808	-0.084	208	220	0	DHLTVEELAQFLK
phospholipase C, eta 1	1611.755	1611.749	0.006	308	320	1	***VDMYARVLQEGCR***
phospholipase C, eta 1	1611.755	1611.882	-0.127	1482	1495	1	SLEPLDALTEQLRK
phospholipase C, eta 1	1657.758	1657.809	-0.051	695	710	0	GTFNPFSGDPLPANPK
phospholipase C, eta 1	1667.756	1667.818	-0.062	104	118	1	YLMAGISDEDSLAKR
phospholipase C, eta 1	1723.823	1723.907	-0.084	248	262	1	VKNVLGIEGFTNFMR
phospholipase C, eta 1	1739.803	1739.902	-0.099	248	262	1	***VKNVLGIEGFTNFMR***
phospholipase C, eta 1	1800.84	1800.882	-0.042	620	633	1	***SEQFMIYNQKQLTR***
phospholipase C, eta 1	1959.871	1959.983	-0.112	136	152	0	NGDGLLNIEEIHQLMHK
phospholipase C-alpha	1190.614	1190.593	0.021	63	73	0	LAPEYEAAATR
phospholipase C-alpha	1340.745	1340.676	0.069	448	459	0	GFPTIYFSPANK
phospholipase C-alpha	1346.766	1346.694	0.072	62	73	1	RLAPEYEAAATR
phospholipase C-alpha	1396.748	1396.698	0.05	471	481	0	ELNDFISYLQR

Protein Name	Measured Mass (Da)	Calculated Mass (Da)	Error	Peptide Start	Peptide End	Missed cleavage	Sequence
phospholipase C-alpha	1431.811	1431.771	0.039	63	75	1	LAPEYEAAATRLK
phospholipase C-alpha	1487.755	1487.671	0.084	335	346	1	***FVMQEEFSRDGK***
phospholipase C-alpha	1651.777	1651.739	0.038	433	447	0	***MDATANDVPSPYEVK***
phospholipase C-alpha	1757.921	1757.894	0.027	130	145	1	QAGPASVPLRTEEEFK
phospholipase C-alpha	1970.017	1969.989	0.028	347	362	1	ALEQFLQEYFDGNLKR
Phospholipase D1	972.449	972.462	-0.013	620	628	0	HSTDTGSIR
Phospholipase D1	1136.622	1136.607	0.015	181	189	1	KQLEDYLTK
Phospholipase D1	1190.582	1190.621	-0.039	540	549	1	IVDETDMKLK
Phospholipase D1	1427.733	1427.701	0.031	869	879	0	WINYISFCGLR
Phospholipase D1	1477.79	1477.713	0.077	676	688	0	***MPWHDIGSVVHGK***
Phospholipase D1	1490.831	1490.766	0.065	318	329	1	WWGGAIEEFIRK
Phospholipase D1	1520.796	1520.762	0.034	340	353	0	FGSYAALHENTLAK
Phospholipase D1	1581.94	1581.869	0.07	409	421	1	***AQQGVRIFIMLYK***
Phospholipase D1	1620.847	1620.92	-0.073	592	605	0	SHQNLIHGLKPHLK
Phospholipase D1	1719.811	1719.778	0.033	230	245	1	SGGHRIPGVNCCGHGR
Phospholipase D1	1719.811	1719.872	-0.061	1004	1017	0	CLPNDEVHNLIQLR
Phospholipase D1	1740.841	1740.925	-0.084	853	868	0	GESSILEQLKPELGNK
Phospholipase D1	1769.868	1769.833	0.035	487	501	1	WDDNEHRLTDVGSVK
Phospholipase D1	1775.848	1775.888	-0.04	676	691	1	***MPWHDIGSVVHGKAAR***
Phospholipase D1	1775.848	1775.776	0.072	235	249	1	IPGVNCCGHGRACYR
Phospholipase D1	1836.905	1836.936	-0.031	985	1000	1	EIWVSTAARNATIYDK
Phospholipase D1	2162.046	2161.977	0.069	775	791	0	HYIYIENQFFISCADDK
phosphomannomutase 2	990.5	990.495	0.005	235	242	1	RICEGLFP
phosphomannomutase 2	1156.604	1156.602	0.002	120	130	0	NGMLNVSPIGR
phosphomannomutase 2	1172.603	1172.597	0.006	120	130	0	***NGMLNVSPIGR***
phosphomannomutase 2	1183.621	1183.612	0.01	138	146	1	IEFYELDKK
phosphomannomutase 2	1335.681	1335.703	-0.022	35	47	1	TKIGVVGGSDFEK
phosphomannomutase 2	1408.714	1408.683	0.032	222	234	0	TVGYTVTAPEDTR
phosphomannomutase 2	1564.791	1564.784	0.007	222	235	1	TVGYTVTAPEDTRR
phosphomannomutase 2	1581.768	1581.77	-0.002	18	30	1	***QKITEEMDGFLQK***
phosphomannomutase 2	1685.734	1685.746	-0.012	207	221	0	TMPGGNDHEIFTDPR
phosphomannomutase 2	1701.737	1701.741	-0.004	207	221	0	***TMPGGNDHEIFTDPR***
phosphomannomutase 2	2184.044	2184.036	0.007	1	19	1	***MATLCLFDMDGTLTAPRQK***
phosphomannomutase 2	2199.95	2200.031	-0.081	1	19	1	***MATLCLFDMDGTLTAPRQK***
Piwi/argonaute family protein meIF2C4	1542.808	1542.833	-0.025	404	417	1	VLPAPMLQYGGRNK
Piwi/argonaute family protein meIF2C4	1599.762	1599.807	-0.045	666	679	1	IIYYRGGVSEGQMK
Piwi/argonaute family protein meIF2C4	1626.773	1626.745	0.028	74	87	1	***MQIFGDRQPGYDGK***
Piwi/argonaute family protein meIF2C4	1626.773	1626.868	-0.095	361	374	2	SAPDRQEEISRLVK
Piwi/argonaute family protein meIF2C4	1649.807	1649.84	-0.033	346	360	1	LTDNQTSTMIKATAR
Piwi/argonaute family protein meIF2C4	1657.786	1657.838	-0.052	254	267	2	***GLKVEVTHCGQMKR***
Piwi/argonaute family protein meIF2C4	1669.804	1669.835	-0.031	312	325	0	HPHLPCLQVGQEQK
Piwi/argonaute family protein meIF2C4	1723.821	1723.867	-0.046	600	616	0	***KPSIAAVVGSMDGHPSR***
Piwi/argonaute family protein meIF2C4	1782.83	1782.846	-0.016	74	88	2	***MQIFGDRQPGYDGKR***
Piwi/argonaute family protein meIF2C4	1785.838	1785.933	-0.095	254	268	3	***GLKVEVTHCGQMKRK***
Piwi/argonaute family protein meIF2C4	1790.807	1790.902	-0.095	257	270	3	VEVTHCGQMKRKYR
Piwi/argonaute family protein meIF2C4	1797.841	1797.825	0.016	847	861	0	AVQIHHDTQHTMYFA
Piwi/argonaute family protein meIF2C4	1800.854	1800.976	-0.122	543	558	1	NVVKTSPQTLSNLCLK
Piwi/argonaute family protein meIF2C4	1868.877	1868.956	-0.079	326	341	0	HTYLPLEVCNIVAGQR
Piwi/argonaute family protein meIF2C4	1883.879	1883.97	-0.091	251	266	2	EIRGLKVEVTHCGQMK
Piwi/argonaute family protein meIF2C4	1888.893	1888.985	-0.092	526	542	1	RVGDTLLGMATQCVQIK
Piwi/argonaute family protein meIF2C4	1900.89	1901.005	-0.115	59	73	3	***RVNREVVDTMVRHFK***

Protein Name	Measured Mass (Da)	Calculated Mass (Da)	Error	Peptide Start	Peptide End	Missed cleavage	Sequence
platelet-derived growth factor A receptor	1006.511	1006.508	0.003	167	175	0	LVPASYDSR
platelet-derived growth factor A receptor	1138.571	1138.566	0.005	972	981	1	SDHPAVARMR
platelet-derived growth factor A receptor	1307.635	1307.581	0.054	842	852	0	DIMHDSNYVSK
platelet-derived growth factor A receptor	1474.642	1474.704	-0.063	1000	1011	1	LKDWESGLDEQR
platelet-derived growth factor A receptor	1546.76	1546.802	-0.042	197	209	1	TFKTSEFNVYALK
platelet-derived growth factor A receptor	1563.796	1563.728	0.068	805	817	1	***GMEFLASKNCVHR***
platelet-derived growth factor A receptor	1661.802	1661.789	0.013	704	718	0	DLDIFGLNPADESTR
platelet-derived growth factor A receptor	1678.778	1678.834	-0.056	736	749	1	QADTTQYVPMLERK
platelet-derived growth factor A receptor	1789.843	1789.884	-0.041	703	718	1	KDLDIFGLNPADESTR
platelet-derived growth factor A receptor	2058.956	2058.937	0.019	982	999	1	VDSDNAYIGVTYKNEEDK
platelet-derived growth factor A receptor	1006.511	1006.508	0.003	167	175	0	LVPASYDSR
platelet-derived growth factor A receptor	1138.571	1138.566	0.005	972	981	1	SDHPAVARMR
platelet-derived growth factor A receptor	1307.635	1307.581	0.054	842	852	0	DIMHDSNYVSK
platelet-derived growth factor A receptor	1474.642	1474.704	-0.063	1000	1011	1	LKDWESGLDEQR
platelet-derived growth factor A receptor	1546.76	1546.802	-0.042	197	209	1	TFKTSEFNVYALK
platelet-derived growth factor A receptor	1563.796	1563.728	0.068	805	817	1	***GMEFLASKNCVHR***
platelet-derived growth factor A receptor	1661.802	1661.789	0.013	704	718	0	DLDIFGLNPADESTR
platelet-derived growth factor A receptor	1678.778	1678.834	-0.056	736	749	1	QADTTQYVPMLERK
platelet-derived growth factor A receptor	1789.843	1789.884	-0.041	703	718	1	KDLDIFGLNPADESTR
platelet-derived growth factor A receptor	2058.956	2058.937	0.019	982	999	1	VDSDNAYIGVTYKNEEDK
plenty-of-prolines-101	941.495	941.515	-0.02	177	184	2	SRSPSPRR
plenty-of-prolines-101	953.503	953.538	-0.035	299	305	3	RHRSRSR
plenty-of-prolines-101	959.546	959.526	0.02	164	170	3	EKRERSR
plenty-of-prolines-101	962.476	962.537	-0.061	278	285	3	SRSKSRSR
plenty-of-prolines-101	962.476	962.537	-0.061	276	283	3	SRSRSKSR
plenty-of-prolines-101	984.542	984.571	-0.029	433	441	2	TSGKVTKHK
plenty-of-prolines-101	984.542	984.546	-0.004	526	534	2	SASPSPRKR
plenty-of-prolines-101	997.502	997.53	-0.028	672	680	1	SPSLSSKHR
plenty-of-prolines-101	1000.48	1000.541	-0.061	534	541	2	RQKETSPR
plenty-of-prolines-101	1000.48	1000.545	-0.065	547	554	1	RWQSPVTK
plenty-of-prolines-101	1015.503	1015.556	-0.053	615	622	1	YSPPIQRR
plenty-of-prolines-101	1015.503	1015.556	-0.053	614	621	1	RYSPPIQR
plenty-of-prolines-101	1081.546	1081.562	-0.016	508	517	1	SHVKNGEVGR
plenty-of-prolines-101	1088.561	1088.595	-0.034	700	708	1	RHSPSPRPR
plenty-of-prolines-101	1092.575	1092.59	-0.015	290	298	1	SPSHTRPRR
plenty-of-prolines-101	1107.556	1107.612	-0.056	336	344	3	HRRSRSPGR
plenty-of-prolines-101	1118.537	1118.58	-0.043	196	204	2	SHSRSPRHR
plenty-of-prolines-101	1125.609	1125.625	-0.016	672	681	2	SPSLSSKHRK
plenty-of-prolines-101	1128.563	1128.636	-0.073	533	541	3	KRQKETSPR
plenty-of-prolines-101	1142.582	1142.624	-0.042	329	337	2	RMPPPPRHR
plenty-of-prolines-101	1142.582	1142.624	-0.042	330	338	2	MPPPPRHRR
plenty-of-prolines-101	1142.582	1142.593	-0.01	258	267	2	EPSPEKNSKK
plenty-of-prolines-101	1153.594	1153.631	-0.037	671	680	2	RSPSLSSKHR
plenty-of-prolines-101	1179.658	1179.537	0.121	537	546	1	***ETSPRMQMGK***
plenty-of-prolines-101	1179.658	1179.622	0.036	288	297	1	SRSPSHTRPR
plenty-of-prolines-101	1181.59	1181.626	-0.036	691	700	2	EARSPQPNKR
plenty-of-prolines-101	1224.66	1224.657	0.003	730	740	0	QSPSPSTRPIR
plenty-of-prolines-101	1231.638	1231.609	0.029	471	481	1	MAAADSVQQRR
plenty-of-prolines-101	1302.722	1302.663	0.059	819	830	3	NSDQEGGGKRKK
plenty-of-prolines-101	2162.035	2162.071	-0.036	537	554	3	***ETSPRMQMGKRWQSPVTK***
plenty-of-prolines-101	2288.12	2288.2	-0.08	209	229	3	SPSPAPEKKEKSPELPEPSVR

Protein Name	Measured Mass (Da)	Calculated Mass (Da)	Error	Peptide Start	Peptide End	Missed cleavage	Sequence
pORF2	958.518	958.559	-0.041	927	934	1	GVKTIQWK
pORF2	975.434	975.473	-0.039	745	752	0	***SMAFLYTK***
pORF2	1014.585	1014.643	-0.058	863	871	2	IAKSLLKDK
pORF2	1033.627	1033.592	0.035	1063	1070	2	ELKKVDFR
pORF2	1114.62	1114.707	-0.087	304	314	2	GKLIALSASKK
pORF2	1114.62	1114.609	0.011	753	761	2	NKQAEKEIR
pORF2	1140.59	1140.665	-0.075	101	110	1	EGHFILIKGK
pORF2	1162.612	1162.626	-0.014	171	180	1	DTVKLTEVMK
pORF2	1213.711	1213.786	-0.076	306	316	3	LIALSASKKKR
pORF2	1213.711	1213.666	0.045	330	340	2	ALEKKEANSPK
pORF2	1561.824	1561.849	-0.025	168	180	2	***LNRDTVKLTEVMK***
pORF2	1601.798	1601.929	-0.131	493	505	1	EDLIPILHKLFHK
pORF2	1607.784	1607.804	-0.02	954	967	1	MGIDPYLSPCTKVK
pORF2	1617.767	1617.746	0.021	197	210	0	EYTFFSAPHGTFSK
pORF2	1634.76	1634.898	-0.138	1052	1065	2	SDRGLISNIYKELK
pORF2	1651.779	1651.839	-0.06	1001	1014	2	FLNRTAMACAVRSR
pORF2	1657.798	1657.849	-0.051	745	758	2	SMAFLYTKNKQAEK
pORF2	1773.879	1773.908	-0.029	740	754	2	***INSNKSMAFLYTKNK***
pORF2	1836.91	1837.016	-0.106	314	329	3	KKRETAHTSSLTTHLK
pORF2	1844.931	1844.916	0.015	1197	1212	0	***DTCSTMFIAALFIIAR***
pORF2	2010.002	2010.042	-0.04	1015	1030	3	IDKWDLMKLQSFCKAK
pORF2	2019.027	2019.013	0.014	181	196	2	QMDLTDIYRTFYPKTK
pORF2	2177.056	2177.201	-0.145	1101	1118	3	KCSTSLIIREMQIKTTLR
pORF2	2224.138	2224.148	-0.01	720	739	1	NSTRELLNLINSFGEVAGYK
Potassium voltage gated channel, Shab-related subfamily, member 1	1462.708	1462.804	-0.096	35	47	0	LNVGGLAHEVLWR
Potassium voltage gated channel, Shab-related subfamily, member 1	1521.7	1521.772	-0.072	88	100	0	HPGAFTSILNFYR
Potassium voltage gated channel, Shab-related subfamily, member 1	1521.7	1521.789	-0.089	475	487	2	KDKVQDNHLSPNK
Potassium voltage gated channel, Shab-related subfamily, member 1	1543.736	1543.81	-0.074	776	790	1	SLHGSTSPKFSLGAR
Potassium voltage gated channel, Shab-related subfamily, member 1	1592.798	1592.779	0.019	503	516	2	SFETKEQGSPEKAR
Potassium voltage gated channel, Shab-related subfamily, member 1	1639.723	1639.78	-0.057	442	456	1	NGSIVSMNMKDAFAR
Potassium voltage gated channel, Shab-related subfamily, member 1	1655.814	1655.775	0.039	442	456	1	***NGSIVSMNMKDAFAR***
Potassium voltage gated channel, Shab-related subfamily, member 1	1703.774	1703.829	-0.055	137	149	2	YHQKKEQMNEELK
Potassium voltage gated channel, Shab-related subfamily, member 1	1707.79	1707.869	-0.079	476	489	2	DKVQDNHLSPNKWK
Potassium voltage gated channel, Shab-related subfamily, member 1	1740.806	1740.948	-0.142	658	672	3	SSMKTHNPMKLRALK
Potassium voltage gated channel, Shab-related subfamily, member 1	1755.874	1755.974	-0.1	310	325	1	LARHSTGLQSLGFTLR
Potassium voltage gated channel, Shab-related subfamily, member 1	1793.891	1793.942	-0.051	488	502	3	WKWTKRALSETSSSK
Potassium voltage gated channel, Shab-related subfamily, member 1	1835.886	1835.942	-0.057	88	103	1	HPGAFTSILNFYRTGR
Potassium voltage gated channel, Shab-related subfamily, member 1	1835.886	1835.964	-0.078	475	489	3	KDKVQDNHLSPNKWK
Potassium voltage gated channel, Shab-related subfamily, member 1	1852.88	1852.906	-0.026	452	467	1	DAFARSIEMMDIVVEK
Potassium voltage gated channel, Shab-related subfamily, member 1	1947.885	1947.964	-0.078	457	474	1	SIEMMDIVVEKNGEGVAK
Potassium voltage gated channel, Shab-related subfamily, member 1	1979.96	1979.953	0.007	457	474	1	***SIEMMDIVVEKNGEGVAK***
progesterone-induced blocking factor 1 isoform a	1014.559	1014.513	0.046	743	750	1	KEAQEWPK
progesterone-induced blocking factor 1 isoform a	1016.536	1016.586	-0.05	660	668	1	EKSALLQTK
progesterone-induced blocking factor 1 isoform a	1115.619	1115.557	0.063	412	421	1	EARDNALAEK
progesterone-induced blocking factor 1 isoform a	1162.621	1162.645	-0.025	628	636	1	YLIESVRQR
progesterone-induced blocking factor 1 isoform a	1199.585	1199.65	-0.065	106	115	0	LDNQLTIQQK
progesterone-induced blocking factor 1 isoform a	1208.621	1208.576	0.044	206	216	0	SELSEELSTSK
progesterone-induced blocking factor 1 isoform a	1545.702	1545.799	-0.097	646	659	1	STAQLEKDVSNLNK
progesterone-induced blocking factor 1 isoform a	1572.849	1572.871	-0.022	305	318	0	EVSSLQQTVTLLQK
progesterone-induced blocking factor 1 isoform a	1603.811	1603.798	0.013	713	726	1	***SVTENQAKTLNMPR***
progesterone-induced blocking factor 1 isoform a	1640.764	1640.829	-0.065	158	170	1	DFELTEEQYVKLK

Protein Name	Measured Mass (Da)	Calculated Mass (Da)	Error	Peptide Start	Peptide End	Missed cleavage	Sequence
progesterone-induced blocking factor 1 isoform a	1652.79	1652.793	-0.003	321	333	1	***DYLNRQNMELSVR***
progesterone-induced blocking factor 1 isoform a	1655.728	1655.795	-0.068	79	91	1	***MDYLTKIEELEEK***
progesterone-induced blocking factor 1 isoform a	1676.821	1676.818	0.003	699	712	1	HSENNLFLTKMESK
progesterone-induced blocking factor 1 isoform a	2289.177	2289.16	0.017	690	708	1	QIIINMCSKHSENNLFLTK
prohibitin	1022.562	1022.491	0.07	178	186	0	EFTEAVEAK
prohibitin	1022.562	1022.566	-0.004	36	43	1	AVIFDRFR
prohibitin	1148.593	1148.582	0.011	134	143	0	FDAGELITQR
prohibitin	1605.843	1605.835	0.008	240	253	1	KLEAAEDIAYQLSR
prohibitin	1660.892	1660.889	0.003	129	143	1	SVVARFDAGELITQR
prohibitin	1732.9	1732.91	-0.01	134	148	1	FDAGELITQRELVSR
prohibitin	1853.942	1854.024	-0.082	256	272	0	NITYLPAGQSVLLQLPQ
prohibitin	1997.07	1997.078	-0.009	220	239	0	AAELIANSLATAGDGLIELR
prohibitin	2097.167	2097.157	0.01	254	272	1	SRNITYLPAGQSVLLQLPQ
prohibitin	2654.362	2654.367	-0.006	106	128	1	IYTSIGEDYDERVLPSITTEILK
Proliferation-associated protein 1	983.495	983.471	0.024	264	271	0	AFFSEVER
Proliferation-associated protein 1	1112.558	1112.532	0.026	273	281	0	***FDAMPFTLR***
Proliferation-associated protein 1	1139.589	1139.539	0.05	21	30	1	***YKMGGDIANR***
Proliferation-associated protein 1	1139.589	1139.572	0.017	264	272	1	AFFSEVERR
Proliferation-associated protein 1	1181.615	1181.597	0.018	145	155	0	AAHLCAEAALR
Proliferation-associated protein 1	1216.638	1216.634	0.004	23	33	1	***MGGDIANRVLR***
Proliferation-associated protein 1	1268.654	1268.633	0.021	272	281	1	***RFDAMPFTLR***
Proliferation-associated protein 1	1850.867	1850.929	-0.062	34	51	0	SLVEASSSGVSVLSLCEK
Proliferation-associated protein 1	2184.056	2184.044	0.011	173	191	0	***VAHSFNCTPIEGMLSHQLK***
proline arginine rich coiled coil 1	916.432	916.436	-0.004	691	697	1	EEAERQR
proline arginine rich coiled coil 1	1312.757	1312.732	0.025	313	324	2	SRSAVTLPRNGR
proline arginine rich coiled coil 1	1312.757	1312.634	0.123	322	334	2	NGRDQGRGSGPGR
proline arginine rich coiled coil 1	1473.802	1473.669	0.133	666	677	1	EKAQAEQEEQER
proline arginine rich coiled coil 1	1656.748	1656.817	-0.069	639	652	2	EEQLAREAEARAER
proline arginine rich coiled coil 1	1789.796	1789.898	-0.102	753	768	1	***AVETRPSGLQKDSMQK***
proline arginine rich coiled coil 1	1818.772	1818.863	-0.091	114	131	0	SSQPSPTTVSAYDSPPAK
proline arginine rich coiled coil 1	1851.804	1851.9	-0.096	366	383	1	SASASPLTPCSAPRSAHR
proline arginine rich coiled coil 1	916.432	916.436	-0.004	659	665	1	REEQEAR
proline arginine rich coiled coil 1	984.525	984.557	-0.032	176	182	2	QLQERRR
proline arginine rich coiled coil 1	1098.628	1098.611	0.016	613	620	3	RRQAREQR
proline arginine rich coiled coil 1	1142.66	1142.651	0.008	628	636	3	KLQAERDKR
proline arginine rich coiled coil 1	1156.673	1156.667	0.006	431	440	2	SALARERNLK
proline arginine rich coiled coil 1	1174.675	1174.649	0.026	718	726	2	LEEIMKRTR
proline arginine rich coiled coil 1	1174.675	1174.594	0.081	420	428	3	KDKERENEK
proline arginine rich coiled coil 1	1279.739	1279.747	-0.008	442	452	1	RQSLPASIRPR
protein disulfide-isomerase (EC 5.3.4.1) ERp61 precursor	1190.614	1190.593	0.021	63	73	0	LAPEYEAAATR
protein disulfide-isomerase (EC 5.3.4.1) ERp61 precursor	1340.745	1340.676	0.069	449	460	0	GFPTIYFSPANK
protein disulfide-isomerase (EC 5.3.4.1) ERp61 precursor	1346.766	1346.694	0.072	62	73	1	RLAPEYEAAATR
protein disulfide-isomerase (EC 5.3.4.1) ERp61 precursor	1396.748	1396.698	0.05	472	482	0	ELNDFISYLQR
protein disulfide-isomerase (EC 5.3.4.1) ERp61 precursor	1431.811	1431.771	0.039	63	75	1	LAPEYEAAATRLK
protein disulfide-isomerase (EC 5.3.4.1) ERp61 precursor	1487.755	1487.671	0.084	336	347	1	***FVMQEEFSRDGK***
protein disulfide-isomerase (EC 5.3.4.1) ERp61 precursor	1651.777	1651.758	0.019	105	119	1	IFRDGEEAGAYDGPR
protein disulfide-isomerase (EC 5.3.4.1) ERp61 precursor	1651.777	1651.739	0.038	434	448	0	***MDATANDVPSPYEVK***
protein disulfide-isomerase (EC 5.3.4.1) ERp61 precursor	1757.921	1757.894	0.027	131	146	1	QAGPASVPLRTEEEFK
protein disulfide-isomerase (EC 5.3.4.1) ERp61 precursor	1970.017	1969.989	0.028	348	363	1	ALEQFLQEYFDGNLKR
protein disulfide-isomerase (EC 5.3.4.1) ERp61 precursor	2590.282	2590.29	-0.009	306	329	0	TFSHELSDFGLESTTGEVPVVAIR
protein disulfide-isomerase (EC 5.3.4.1) ERp61 precursor	2718.398	2718.385	0.013	305	329	1	KTFSHELSDFGLESTTGEVPVVAIR

Protein Name	Measured Mass (Da)	Calculated Mass (Da)	Error	Peptide Start	Peptide End	Missed cleavage	Sequence
protein tyrosine phosphatase, receptor type, R	1070.667	1070.59	0.077	327	335	0	MKPIGLQER
protein tyrosine phosphatase, receptor type, R	1098.667	1098.557	0.11	25	33	0	NNDHFLAIR
protein tyrosine phosphatase, receptor type, R	1136.699	1136.571	0.128	53	62	0	SLDIAQEAYK
protein tyrosine phosphatase, receptor type, R	1237.73	1237.604	0.125	210	220	0	NVLQGQHEADK
protein tyrosine phosphatase, receptor type, R	1307.778	1307.676	0.102	581	593	0	GPVVVHCSAGIGR
protein tyrosine phosphatase, receptor type, R	1454.773	1454.682	0.091	284	296	0	***TTHSMVQPDQAPK***
protein tyrosine phosphatase, receptor type, R	1524.776	1524.731	0.045	63	75	0	HNYHSPSEVQISK
protocadherin gamma subfamily C	1098.648	1098.591	0.056	755	764	0	IQLGSEDPIK
protocadherin gamma subfamily C	1142.677	1142.654	0.023	80	90	0	VDLDSGALLIK
protocadherin gamma subfamily C	1199.703	1199.65	0.053	544	555	0	GSPPLSSTVTVR
protocadherin gamma subfamily C	1307.77	1307.73	0.04	743	754	1	REGLPPSNGILR
protocadherin gamma subfamily C	1307.77	1307.719	0.051	213	225	0	LVLTAVDGGNPPR
protocadherin gamma subfamily C	1770.804	1770.788	0.016	325	340	1	ARDGGSPAMEQHCSLR
Prpf8	1489.78	1489.759	0.021	752	764	0	GSELQLPFQACLK
Prpf8	1734.821	1734.795	0.026	488	502	0	***MNSSCADILLFASYK***
Prpf8	1887.856	1887.878	-0.022	221	236	0	SGMSHEEDQLIPNLYR
Prpf8	2097.999	2098.007	-0.008	775	791	0	***ATEPQMVLFNLYDDWLK***
Prpf8	2162.042	2162.18	-0.138	733	751	0	GMLDPLEVHLLDFPNIVIK
Prpf8	2238.03	2238.149	-0.119	187	207	0	***ELGGLGMLSMGHVLIPQSDLR***
Prpf8	2254.07	2254.144	-0.074	187	207	0	***ELGGLGMLSMGHVLIPQSDLR***
Prpf8	2336.109	2336.198	-0.089	411	431	0	ANVYVGFQVQLDLTGIFMHGK
PTK2 protein tyrosine kinase 2 beta	972.436	972.487	-0.051	688	695	0	DIAIEQER
PTK2 protein tyrosine kinase 2 beta	1114.615	1114.58	0.035	217	225	0	QMQENLKPK
PTK2 protein tyrosine kinase 2 beta	1937.977	1937.918	0.059	915	931	1	***MKLAQQNAVTSLSEDCK***
PTK2 protein tyrosine kinase 2 beta	2162.067	2162.097	-0.031	40	57	2	ILKVCFYSNSFNPGKNFK
PTK2 protein tyrosine kinase 2 beta	2188.125	2188.12	0.005	226	242	2	***QFRKMIQQTFQQYASLR***
PTK2 protein tyrosine kinase 2 beta	2253.129	2253.175	-0.046	170	188	2	VSEGMALQLGCLELRRFFK
PTK2 protein tyrosine kinase 2 beta	2272.115	2272.13	-0.015	36	54	2	EDVRILKVCFYSNSFNPGK
PTK2 protein tyrosine kinase 2 beta	2280.149	2280.171	-0.022	166	185	2	YASKVSEGMALQLGCLELRR
PTK2 protein tyrosine kinase 2 beta	2559.198	2559.23	-0.032	129	148	2	YDLQIRYLPEDFMESLKEDR
Pyrroline-5-carboxylate reductase-like	917.534	917.519	0.015	10	18	1	RVGFVGAGR
Pyrroline-5-carboxylate reductase-like	1534.886	1534.803	0.083	219	231	1	MLQQEGKHPAQLR
Pyrroline-5-carboxylate reductase-like	1550.837	1550.798	0.039	219	231	1	***MLQQEGKHPAQLR***
Pyrroline-5-carboxylate reductase-like	1841.904	1841.884	0.02	37	52	0	QVLASAPTDNNLCHFR
Pyrroline-5-carboxylate reductase-like	1988.992	1988.947	0.045	123	140	0	***VSPNLPCVVQEGAMVMAR***
Pyrroline-5-carboxylate reductase-like	2001.085	2001.063	0.022	232	250	0	TDVLTPAGTTIHGLHALER
RAB11a, member RAS oncogene family	943.479	943.476	0.003	75	82	0	AITSAYYR
RAB11a, member RAS oncogene family	1079.56	1079.561	-0.001	42	51	0	STIGVEFATR
RAB11a, member RAS oncogene family	1159.573	1159.562	0.011	96	104	0	HLTYENVER
RAB11a, member RAS oncogene family	1273.623	1273.604	0.019	62	72	0	AQIWDTAGQER
RAB11a, member RAS oncogene family	1288.732	1288.739	-0.006	83	95	0	GAVGALLVYDIAK
RAB11a, member RAS oncogene family	2643.32	2643.295	0.025	186	209	0	ENDMSPSNNVVPIHVPPTTENKPK
RAB11a, member RAS oncogene family	2659.315	2659.29	0.025	186	209	0	***ENDMSPSNNVVPIHVPPTTENKPK***
Rab6-interacting protein 2 isoform A	1105.671	1105.57	0.101	431	439	2	SHSKFMKNK
Rab6-interacting protein 2 isoform A	1107.718	1107.595	0.123	535	543	2	***ETMLNKKTK***
Rab6-interacting protein 2 isoform A	1117.682	1117.597	0.085	779	788	0	IAELESLTSR
Rab6-interacting protein 2 isoform A	1134.688	1134.607	0.081	188	196	1	TFWSPELKK
Rab6-interacting protein 2 isoform A	1141.705	1141.609	0.096	580	588	0	IENLQEQLR
Rab6-interacting protein 2 isoform A	1143.71	1143.624	0.086	914	923	1	TQEEVAALKR
Rab6-interacting protein 2 isoform A	1171.761	1171.667	0.094	628	636	2	TIERLKEQR
Rab6-interacting protein 2 isoform A	1171.761	1171.68	0.08	698	707	1	TLEIALEQKK

Protein Name	Measured Mass (Da)	Calculated Mass (Da)	Error	Peptide Start	Peptide End	Missed cleavage	Sequence
Rab6-interacting protein 2 isoform A	1171.761	1171.644	0.117	888	898	0	QEALLAAISEK
Rab6-interacting protein 2 isoform A	1199.727	1199.65	0.077	461	472	0	AAGLQSEIGQVK
Rab6-interacting protein 2 isoform A	1220.728	1220.654	0.074	593	602	2	***QMSSLKERVK***
Rab6-interacting protein 2 isoform A	1243.791	1243.713	0.078	912	922	2	KKTQEEVAALK
Rab6-interacting protein 2 isoform A	1258.805	1258.713	0.092	478	488	1	KDTELLALQTK
Rab6-interacting protein 2 isoform A	1284.789	1284.765	0.024	696	706	1	LKTLEIALEQK
Rab6-interacting protein 2 isoform A	1288.809	1288.687	0.122	440	450	1	VEQLKEELSSK
Rab6-interacting protein 2 isoform A	1305.797	1305.67	0.126	809	819	2	***KKSAQMLEEAR***
Rab6-interacting protein 2 isoform A	1305.797	1305.695	0.101	844	854	2	***VKQELESMKAK***
Rab6-interacting protein 2 isoform A	1413.82	1413.72	0.099	7	20	1	SVGKVEPSSQSPGR
Rab6-interacting protein 2 isoform A	1426.804	1426.76	0.044	877	887	2	***RKHLEEVLEMK***
Rab6-interacting protein 2 isoform A	1441.807	1441.679	0.128	320	332	1	GLSAKATEEDHER
rac GTPase activating protein; GAB-associated CDC42	1134.6	1134.54	0.06	819	829	0	STSAPLTDSEK
rac GTPase activating protein; GAB-associated CDC42	1378.696	1378.808	-0.112	171	182	0	NLAIVWAPNLLR
rac GTPase activating protein; GAB-associated CDC42	1396.745	1396.61	0.135	117	129	0	FSDAVSAATDEER
rac GTPase activating protein; GAB-associated CDC42	1396.745	1396.608	0.137	1197	1208	0	NMSGHHSKPCSR
rac GTPase activating protein; GAB-associated CDC42	1431.787	1431.765	0.022	1237	1249	0	VQSLHAPPPSMIR
rac GTPase activating protein; GAB-associated CDC42	1673.878	1673.774	0.104	357	372	0	SEESLTSLHAVDGDSK
rac GTPase activating protein; GAB-associated CDC42	1885.967	1885.989	-0.022	936	953	0	VLAEQPSAADFVAATLQR
rac GTPase activating protein; GAB-associated CDC42	1952.901	1952.901	0	1284	1299	0	SDYHVTQLQPYFENGR
rac GTPase activating protein; GAB-associated CDC42	1959.938	1960.029	-0.091	695	713	0	MLALALAESAQQASSQTLK
rac GTPase activating protein; GAB-associated CDC42	1983.986	1983.925	0.061	1054	1072	0	AESFPGHSCGFAAPVPPTR
RAD50	970.482	970.571	-0.089	1146	1152	1	IIRDLWR
RAD50	985.444	985.541	-0.098	752	759	1	NRLQSVNR
RAD50	1013.497	1013.493	0.004	63	71	0	GNTFVHDPK
RAD50	1013.497	1013.55	-0.053	344	352	0	AELLVEQGR
RAD50	1045.548	1045.553	-0.005	360	367	1	HQEHIRAR
RAD50	1110.548	1110.516	0.032	99	107	1	***SMLCSQKNK***
RAD50	1208.564	1208.549	0.015	1302	1312	0	CSISSLGSYVH
RAD50	1227.597	1227.668	-0.071	754	763	1	LQSVNRDIQR
RAD50	1227.597	1227.592	0.005	1104	1112	1	YREMMIVMR
RAD50	1340.624	1340.672	-0.048	174	184	1	QKFDEIFSATR
RAD50	1340.624	1340.711	-0.087	257	267	1	EIEHNLSKIMK
RAD50	1351.62	1351.647	-0.027	787	797	0	***VCLTDVTIMER***
RAD50	1354.647	1354.622	0.025	51	62	0	YICTGDFPPGTK
RAD50	1354.647	1354.625	0.022	618	628	1	KEEQLSSYEDK
RAD50	1505.696	1505.751	-0.055	1078	1089	1	QKGYEDEILHFK
RAD50	1548.724	1548.778	-0.055	1127	1139	1	TLDQAIMKFHSMK
RAD50	1553.728	1553.68	0.047	1000	1012	1	***DMGTMRQDIDTQK***
RAD50	1741.807	1741.84	-0.033	1106	1119	1	***EMMIVMRTTELVNK***
RAD50	1741.807	1741.863	-0.056	1153	1166	1	STYRGQDIEYIEIR
RAD50	1769.869	1769.899	-0.03	84	98	1	LQFRDVNGEMVAVHR
RAD50	1809.825	1809.819	0.005	629	644	0	LFDVCGSQDLESDLGR
RAD50	1867.848	1867.847	0.001	521	535	0	***LDQEMEQLNHHTTTR***
RBM-3 (RNA-binding protein 3)	1022.555	1022.562	-0.007	76	84	1	QIRVDHAGK
RBM-3 (RNA-binding protein 3)	1566.712	1566.684	0.027	115	128	0	YDSRPGGYGYGYGR
RBM-3 (RNA-binding protein 3)	2012.923	2012.904	0.019	48	65	0	***GFGFITFTNPEHASDAMR***
RBM-3 (RNA-binding protein 3)	2622.018	2622.027	-0.009	131	153	2	DYSGSQGGYDRYSGGNYRDNYDN
RBM-3 (RNA-binding protein 3)	2865.171	2865.16	0.011	129	153	3	SRDYSGSQGGYDRYSGGNYRDNYDN
Rho GDP dissociation inhibitor (GDI) alpha	948.571	948.539	0.032	47	54	1	YKEALLGR
Rho GDP dissociation inhibitor (GDI) alpha	992.556	992.529	0.028	102	109	1	EGVEYRIK

Protein Name	Measured Mass (Da)	Calculated Mass (Da)	Error	Peptide Start	Peptide End	Missed cleavage	Sequence
Rho GDP dissociation inhibitor (GDI) alpha	1201.637	1201.618	0.019	30	39	0	SIQEIQELDK
Rho GDP dissociation inhibitor (GDI) alpha	1244.556	1244.549	0.007	138	148	0	TDYMVGSYGPR
Rho GDP dissociation inhibitor (GDI) alpha	1453.746	1453.756	-0.01	96	107	1	QSFVLKEGVEYR
Rho GDP dissociation inhibitor (GDI) alpha	1649.915	1649.909	0.005	55	70	0	VAVSADPNVPNVIVTR
Rho GDP dissociation inhibitor (GDI) alpha	1916.946	1916.932	0.014	30	45	1	SIQEIQELDKDDESLR
Rho interacting protein 3	1219.591	1219.644	-0.053	883	892	0	DAYELEVLLR
Rho interacting protein 3	1655.855	1655.764	0.091	193	207	0	***MVYSICPASLGEASR***
Rho interacting protein 3	1803.836	1803.936	-0.1	466	483	0	HVLPASAPDVTSSLPEGK
Rho interacting protein 3	1835.922	1835.93	-0.008	4	18	0	EIISGWLEMLMVYPR
Rho interacting protein 3	1851.925	1851.925	0	4	18	0	***EIISGWLEMLMVYPR***
Rho interacting protein 3	2015.967	2015.894	0.073	43	64	0	***MAVTSSSGGSSGSSSSIPSAEK***
Rho interacting protein 3	2162.081	2161.997	0.084	301	319	0	***AEHMETNMLILTTPSSDTR***
Rho interacting protein 3	1219.591	1219.644	-0.053	883	892	0	DAYELEVLLR
Rho interacting protein 3	1655.855	1655.764	0.091	193	207	0	***MVYSICPASLGEASR***
Rho interacting protein 3	1803.836	1803.936	-0.1	466	483	0	HVLPASAPDVTSSLPEGK
Rho interacting protein 3	1835.922	1835.93	-0.008	4	18	0	EIISGWLEMLMVYPR
Rho interacting protein 3	1851.925	1851.925	0	4	18	0	***EIISGWLEMLMVYPR***
Rho interacting protein 3	2015.967	2015.894	0.073	43	64	0	***MAVTSSSGGSSGSSSSIPSAEK***
Rho interacting protein 3	2162.081	2161.997	0.084	301	319	0	***AEHMETNMLILTTPSSDTR***
Rho-associated protein kinase 2	974.438	974.466	-0.029	1113	1120	0	DSDIEQLR
Rho-associated protein kinase 2	974.438	974.528	-0.09	676	683	1	LTDLEKEK
Rho-associated protein kinase 2	984.587	984.596	-0.009	775	782	1	LNELLKQK
Rho-associated protein kinase 2	1024.507	1024.537	-0.03	113	121	1	ASQKVYAMK
Rho-associated protein kinase 2	1051.562	1051.609	-0.047	117	125	1	VYAMKLLSK
Rho-associated protein kinase 2	1081.546	1081.478	0.068	730	738	1	SEAMKEMEK
Rho-associated protein kinase 2	1081.546	1081.624	-0.078	30	38	1	LEALIRDPR
Rho-associated protein kinase 2	1114.589	1114.67	-0.082	739	747	2	KLLEERSLK
Rho-associated protein kinase 2	1118.539	1118.568	-0.029	573	582	1	TESDTAARLR
Rho-associated protein kinase 2	1130.574	1130.581	-0.007	483	491	0	ELEEEITLR
Rho-associated protein kinase 2	1174.551	1174.619	-0.068	996	1005	1	LKDSQEQLSK
Rho-associated protein kinase 2	1174.551	1174.619	-0.068	998	1007	1	DSQEQLSKLK
Rho-associated protein kinase 2	1209.599	1209.573	0.026	730	739	2	SEAMKEMEKK
Rho-associated protein kinase 2	1214.611	1214.625	-0.014	781	790	1	QKDVLNEDVR
Rho-associated protein kinase 2	1312.672	1312.734	-0.063	750	760	1	VENLLLEAEKR
Rho-associated protein kinase 2	1312.672	1312.691	-0.02	1062	1071	2	***ENRKLHMELK***
Rho-associated protein kinase 2	1316.649	1316.704	-0.055	493	503	1	SVESTLRQLER
Rho-associated protein kinase 2	1348.712	1348.646	0.066	965	976	0	DTTIASLEETNR
Rho-associated protein kinase 2	1348.712	1348.761	-0.049	1163	1173	3	NNTKKFGWVKK
Rho-associated protein kinase 2	1428.814	1428.768	0.046	526	537	2	KRNLENDVNSLK
Rho-associated protein kinase 2	1465.744	1465.832	-0.088	113	125	2	ASQKVYAMKLLSK
Rho-associated protein kinase 2	1503.711	1503.658	0.053	684	695	0	***SNMEIDMTYQLK***
Rho-associated protein kinase 2	1547.793	1547.844	-0.052	470	482	3	CKSINTRLEKTAK
Rho-associated protein kinase 2	1560.817	1560.913	-0.096	1347	1360	2	KIPKKPPAPDPFAR
Rho-associated protein kinase 2	2143.981	2144	-0.019	721	738	2	IYESIEEAKSEAMKEMEK
Rho-associated protein kinase 2	2143.981	2143.997	-0.016	350	365	1	QHPFFKNDQWNWDNIR
Rho-associated protein kinase 2	2273.082	2273.095	-0.013	826	843	2	***QIKQENNHLMEMKMNLEK***
Rho-associated protein kinase 2	2288.114	2288.09	0.024	721	739	3	***IYESIEEAKSEAMKEMEKK***
RIKEN cDNA 1700021E15	972.476	972.56	-0.084	860	868	0	IIELTGTAR
RIKEN cDNA 1700021E15	972.476	972.514	-0.038	1018	1024	1	NWELKQR
RIKEN cDNA 1700021E15	1098.531	1098.614	-0.083	1074	1082	1	LQNAKEQLR
RIKEN cDNA 1700021E15	1112.553	1112.568	-0.015	630	638	1	ICFAFEKAK

Protein Name	Measured Mass (Da)	Calculated Mass (Da)	Error	Peptide Start	Peptide End	Missed cleavage	Sequence
RIKEN cDNA 1700021E15	1139.585	1139.637	-0.052	884	892	1	MEKEIIHLK
RIKEN cDNA 1700021E15	1204.637	1204.611	0.025	704	713	2	ENMKKDEALK
RIKEN cDNA 1700021E15	1268.655	1268.606	0.049	869	878	1	***QAKLEMDQYK***
RIKEN cDNA 1700021E15	1268.655	1268.606	0.049	842	851	1	MEEKYETAIR
RIKEN cDNA 1700021E15	1284.664	1284.714	-0.05	519	529	1	QRLATGIEELR
RIKEN cDNA 1700021E15	1284.664	1284.601	0.063	842	851	1	***MEEKYETAIR***
RIKEN cDNA 1700021E15	1767.835	1767.837	-0.002	899	913	0	***SMQLSQLDMVLDQTK***
RIKEN cDNA 1700021E15	1852.858	1852.923	-0.065	842	856	2	MEEKYETAIREVDLK
RIKEN cDNA 1700021E15	1926.918	1926.888	0.03	1223	1238	0	EAYQIEMISHQENHAK
RIKEN cDNA 1700021E15	1972.955	1972.988	-0.033	602	617	2	NAELEQELMEKNEKIR
RIKEN cDNA 1700021E15	1988.972	1988.983	-0.011	602	617	2	***NAELEQELMEKNEKIR***
RIKEN cDNA 1700021E15	2207.055	2207.161	-0.106	860	878	2	IIELTGTARQAKLEMDQYK
RIKEN cDNA 1700021E15	2288.123	2288.08	0.043	374	392	1	VATQNERLELCQQDIDNSR
RIKEN cDNA 1700021E15	2288.123	2288.146	-0.023	1005	1022	2	***QELIEMDQALKERNWELK***
RIKEN cDNA 1700021E15	2451.146	2451.172	-0.026	893	913	2	***RDGENKSMQLSQLDMVLDQTK***
RIKEN cDNA 4631416I11	2272.104	2272.169	-0.065	149	169	1	NTPLHYAAASGMKACVELLVK
RIKEN cDNA 4631416I11	2330.048	2330.109	-0.061	638	658	0	ETEGGCPDTTFIEDAVHVLLK
RIKEN cDNA 4631416I11	2405.09	2405.141	-0.051	571	590	1	YEVIQHVEEQSKEMTVEAEK
RIKEN cDNA 4631416I11	2421.063	2421.135	-0.073	571	590	1	***YEVIQHVEEQSKEMTVEAEK***
RIKEN cDNA 4631416I11	2454.167	2454.163	0.004	287	309	0	SGVQMPTPPPSGYNAWDTLPSPR
RIKEN cDNA 4631416I11	2549.256	2549.23	0.026	571	591	2	***YEVIQHVEEQSKEMTVEAEKK***
RIKEN cDNA 4631416I11	2775.269	2775.394	-0.125	106	127	2	***LARPVEDDFRRADCLQMILQWK***
RIKEN cDNA 4631416I11	2973.5	2973.602	-0.102	238	263	2	***RLKDMLIVETADMLQAPLFTAEALLR***
RIKEN cDNA 4631416I11	2144.993	2144.988	0.005	1004	1023	1	DGSEGVRDMELVPPEDSVSK
RIKEN cDNA 4631416I11	2162.015	2162.059	-0.045	128	147	2	***GAKLDQGEYERAAIDAVDNK***
RPTPmam4 isoform III	1060.536	1060.522	0.014	1222	1230	0	SMDVLPLDR
RPTPmam4 isoform III	1199.614	1199.62	-0.005	229	237	0	LWLQQWNGR
RPTPmam4 isoform III	1358.729	1358.796	-0.067	455	465	1	GLRPFMTIRLR
RPTPmam4 isoform III	1388.677	1388.74	-0.063	572	585	1	ASTAKGFGPPVTTR
RPTPmam4 isoform III	1445.806	1445.737	0.069	989	1002	0	HPAEHTVGTATLGR
RPTPmam4 isoform III	1523.773	1523.769	0.004	251	264	1	RFSATVSVADTSQR
RPTPmam4 isoform III	1526.822	1526.798	0.024	238	250	1	***DTALMVTRVVNHR***
RPTPmam4 isoform III	1594.81	1594.783	0.027	275	290	0	SDGGSGVSNYAELIVK
RPTPmam4 isoform III	1606.806	1606.784	0.021	1373	1384	2	RLEKWQEQYDGR
RPTPmam4 isoform III	1648.852	1648.824	0.028	690	703	0	TYNGYWNPPLSPLK
RPTPmam4 isoform III	1683.882	1683.825	0.057	351	363	0	LWHLDPDVEYEIR
RPTPmam4 isoform III	1980.961	1980.97	-0.009	223	237	1	WSQHDKLWLQQWNGR
RPTPmam4 isoform III	1987.95	1987.999	-0.049	791	809	1	***EMGPVASTDKPTAKLGTNR***
RPTPmam4 isoform III	2273.124	2273.184	-0.06	176	195	2	GHPGYIAVDEVRVLAHPCRK
RPTPmam4 isoform III	2288.105	2288.105	0	783	804	1	ETQSGAQREMGPVASTDKPTAK
RPTPmam4 isoform III	2573.248	2573.193	0.055	1377	1398	2	WQEQYDGREGRTVVHCLNGGGR
Sac domain-containing inositol phosphatase 3	1312.785	1312.811	-0.026	368	379	0	FGSPIIILNLVK
Sac domain-containing inositol phosphatase 3	1457.785	1457.816	-0.031	101	112	0	FLEGYYIVLITK
Sac domain-containing inositol phosphatase 3	1461.778	1461.786	-0.008	228	239	0	YVWNGELLDIIK
Sac domain-containing inositol phosphatase 3	1767.801	1767.834	-0.033	127	141	0	***IEDTSMIYIPNDSVR***
Sac domain-containing inositol phosphatase 3	1835.859	1835.902	-0.043	245	259	0	DWLLYIIHGFCGQSK
Sac domain-containing inositol phosphatase 3	1851.855	1851.954	-0.099	642	657	0	***HLPLPYDEVICAANLK***
SAPS domain family, member 2 isoform 1	900.481	900.423	0.058	341	348	0	GNMGHLTR
SAPS domain family, member 2 isoform 1	990.524	990.593	-0.069	1	8	0	LQVIMFLK
SAPS domain family, member 2 isoform 1	1541.792	1541.794	-0.002	59	71	0	IVALIHPHQDEDR
SAPS domain family, member 2 isoform 1	1692.805	1692.831	-0.026	145	160	0	VGTEGLVDSFSQGLER

Protein Name	Measured Mass (Da)	Calculated Mass (Da)	Error	Peptide Start	Peptide End	Missed cleavage	Sequence
SAPS domain family, member 2 isoform 1	1769.784	1769.792	-0.008	381	394	0	WESFVEETLMETNR
SAPS domain family, member 2 isoform 1	1851.796	1851.839	-0.043	657	672	0	TFGPTSPCAWNVCVTR
secreted embryonic phosphatase	972.456	972.51	-0.054	241	249	1	QAGTRLDGR
secreted embryonic phosphatase	1114.618	1114.634	-0.016	33	43	2	KAAEALDAAKK
secreted embryonic phosphatase	2145.042	2145.008	0.034	223	240	2	***KFMFPKGTPDQEYPTDTK***
secreted embryonic phosphatase	2288.138	2288.258	-0.12	53	74	1	NLVILMGDGMGVSTVTATRILK
secreted embryonic phosphatase	2559.186	2559.24	-0.054	323	344	1	GFYLFVEGGRIDHGHHETVAYR
secreted embryonic phosphatase	2808.364	2808.342	0.022	75	99	1	***GQQQGHLGPETQLAMDRFPHMALSK***
secreted embryonic phosphatase	2894.366	2894.502	-0.136	203	228	2	DISTQLISNMDIDVILGGGRKFMFPK
Sepiapterin reductase (SPR)	1193.698	1193.651	0.047	86	95	0	ELPRPEGLQR
Sepiapterin reductase (SPR)	1344.811	1344.743	0.068	31	43	0	***LLSPGSVMLVSAR***
Sepiapterin reductase (SPR)	1568.905	1568.851	0.053	64	79	0	VVLAAADLGTEAGVQR
Sepiapterin reductase (SPR)	1735.892	1735.844	0.047	179	193	0	***DMLYQVLAAEEPSVR***
Sepiapterin reductase (SPR)	1865.933	1865.842	0.09	1	18	0	MEADGLGCAVCVLTGASR
Sepiapterin reductase (SPR)	2003.041	2002.977	0.064	194	211	0	***VLSYAPGPLDNDMQQLAR***
Sepiapterin reductase (SPR)	2034.088	2034.02	0.068	176	193	1	***AARDMLYQVLAAEEPSVR***
serine/arginine repetitive matrix 2	982.525	982.446	0.079	504	512	0	SHSPSSPER
serine/arginine repetitive matrix 2	992.517	992.503	0.014	1705	1713	0	QPSPQPSPR
serine/arginine repetitive matrix 2	1146.589	1146.588	0.001	117	127	0	SSSPVTELTAR
serine/arginine repetitive matrix 2	1242.689	1242.547	0.142	621	632	0	AQSGTDSSPEHK
serine/arginine repetitive matrix 2	1276.751	1276.604	0.147	1455	1466	0	SPVPSAFSDQSR
serine/arginine repetitive matrix 2	1336.788	1336.684	0.104	1347	1360	0	SAHGTAPVNIAGSR
serine/arginine repetitive matrix 2	1356.765	1356.681	0.084	1264	1277	0	***TPAAAAAMNLASPR***
serine/arginine repetitive matrix 2	1380.83	1380.736	0.094	1278	1291	0	TAVAPSAVNLADPR
serine/arginine repetitive matrix 2	1556.927	1556.805	0.122	1211	1224	0	***MSQVPAPVPLMSLR***
SNF2 histone linker PHD RING helicase isoform A	1045.52	1045.503	0.017	1083	1091	1	DGRLEEEAK
SNF2 histone linker PHD RING helicase isoform A	1156.568	1156.646	-0.078	1294	1303	1	AILSFARSHR
SNF2 histone linker PHD RING helicase isoform A	1156.568	1156.54	0.028	508	516	1	NYKEDVFDK
SNF2 histone linker PHD RING helicase isoform A	1190.555	1190.586	-0.031	1092	1100	1	QLREHYMSK
SNF2 histone linker PHD RING helicase isoform A	1208.561	1208.615	-0.054	1153	1162	1	VRNEISSNYK
SNF2 histone linker PHD RING helicase isoform A	1309.598	1309.597	0.001	1227	1236	0	LPLNCCVFCK
SNF2 histone linker PHD RING helicase isoform A	1309.598	1309.618	-0.02	1163	1173	1	***QQTDKLSMSEK***
SNF2 histone linker PHD RING helicase isoform A	1416.697	1416.845	-0.148	956	967	1	RTVSSILYPLLR
SNF2 histone linker PHD RING helicase isoform A	1424.672	1424.766	-0.094	1552	1563	1	IKTFQENLSAFK
SNF2 histone linker PHD RING helicase isoform A	1432.713	1432.777	-0.064	439	451	1	VMILTAVKEMNGK
SNF2 histone linker PHD RING helicase isoform A	1438.686	1438.742	-0.056	232	242	2	***FNQLMKRVMEK***
SNF2 histone linker PHD RING helicase isoform A	1442.682	1442.747	-0.065	1083	1094	2	DGRLEEEAKQLR
SNF2 histone linker PHD RING helicase isoform A	1454.691	1454.737	-0.046	232	242	2	***FNQLMKRVMEK***
SNF2 histone linker PHD RING helicase isoform A	1464.676	1464.767	-0.091	439	451	1	***VMILTAVKEMNGK***
SNF2 histone linker PHD RING helicase isoform A	1484.671	1484.711	-0.04	219	231	2	LDFMSDAGSRMKK
SNF2 histone linker PHD RING helicase isoform A	1507.739	1507.703	0.036	420	431	1	EIQDTEYEPKEK
SNF2 histone linker PHD RING helicase isoform A	1522.749	1522.744	0.005	1281	1293	1	***GLWAISETERSMK***
SNF2 histone linker PHD RING helicase isoform A	1553.726	1553.731	-0.005	1155	1167	1	NEISSNYKQQTDK
SNF2 histone linker PHD RING helicase isoform A	1830.878	1831.024	-0.146	453	468	1	GVSILSIYKYVSSIFR
SNF2 histone linker PHD RING helicase isoform A	1889.853	1889.93	-0.077	1086	1100	2	LEEEAKQLREHYMSK
SNF2 histone linker PHD RING helicase isoform A	2046.981	2047.082	-0.101	878	893	2	***KNPQHLYSFIAKIMWR***
somatostatin receptor 1	1255.605	1255.628	-0.023	122	131	0	HWPFGALLCR
somatostatin receptor 1	2143.02	2142.985	0.035	362	380	0	AYSVEDFQPENLESGGVFR
somatostatin receptor 1	2232.998	2232.991	0.007	198	218	0	TAANSDGTVACNMLMPEPAQR
somatostatin receptor 1	2249.001	2248.986	0.014	198	218	0	***TAANSDGTVACNMLMPEPAQR***
somatostatin receptor 1	2688.262	2688.244	0.018	337	359	0	***ILCLSWMDNAAEEPVDYYATALK***

Protein Name	Measured Mass (Da)	Calculated Mass (Da)	Error	Peptide Start	Peptide End	Missed cleavage	Sequence
Sorting nexin 13	1121.545	1121.596	-0.051	458	467	0	VTVDDYLVAK
Sorting nexin 13	1171.585	1171.598	-0.013	161	170	0	IVDDFGTHLR
Sorting nexin 13	1427.713	1427.772	-0.059	411	423	0	VTAQQQLEVLSGR
Sorting nexin 13	1520.784	1520.808	-0.024	612	624	0	ITEQFENLSSILK
Sorting nexin 13	1564.76	1564.824	-0.064	775	787	0	VMLLLMDEVFDLK
Sorting nexin 13	2986.518	2986.597	-0.08	257	281	0	GILLPLINQLSDPDYINQYVIWMIR
sorting nexin 19	1174.611	1174.663	-0.052	684	694	0	MVVSAIVDTLK
sorting nexin 19	1197.641	1197.515	0.126	843	851	0	***WLCTESMQK***
sorting nexin 19	1219.584	1219.674	-0.09	674	683	1	KPFMVSRIDK
sorting nexin 19	1638.818	1638.734	0.084	617	630	0	***LFPDLPFGNMDSDR***
sorting nexin 19	1712.844	1712.866	-0.022	864	877	0	WLEVQVANLTCPQR
sorting nexin 19	1762.867	1762.964	-0.098	684	699	1	***MVVSAIVDTLKTAFPR***
sorting nexin 19	1787.906	1787.941	-0.035	782	797	1	IQPAEVPDKDPQQVPK
sorting nexin 19	1835.923	1835.883	0.04	548	563	0	EHSGTGFHPYTLYTVK
sorting nexin 19	1899.92	1900.019	-0.099	97	111	1	***QLEQEINRTIQMIIR***
sorting nexin 19	1955.964	1956.028	-0.064	105	119	1	***TIQMIIRDFVLSWYR***
spermidine synthase	1052.588	1052.565	0.023	48	55	0	YQDILVFR
spermidine synthase	1149.627	1149.618	0.009	286	295	0	AAFVLPEFTR
spermidine synthase	1224.714	1224.718	-0.004	97	109	0	VLIIGGGDGGVLR
spermidine synthase	1277.721	1277.713	0.008	286	296	1	AAFVLPEFTRK
spermidine synthase	1295.73	1295.698	0.032	46	55	1	SRYQDILVFR
spermidine synthase	1352.83	1352.813	0.017	96	109	1	KVLIIGGGDGGVLR
Spink5 protein/RIKEN cDNA 2310065D10	972.439	972.492	-0.053	506	513	1	NGPLRCTR
Spink5 protein/RIKEN cDNA 2310065D10	1191.59	1191.584	0.006	757	766	2	RNTETNKSDK
Spink5 protein/RIKEN cDNA 2310065D10	1255.605	1255.567	0.037	356	366	1	RGSEESETYAK
Spink5 protein/RIKEN cDNA 2310065D10	1639.763	1639.812	-0.049	555	568	1	EVKVDCSEYLALSK
Spink5 protein/RIKEN cDNA 2310065D10	1739.78	1739.844	-0.064	418	431	3	VKREAAKEMCSEFR
Spink5 protein/RIKEN cDNA 2310065D10	2144.988	2144.898	0.09	106	125	1	KDGDFICPSDTSSVCGTDGK
Spink5 protein/RIKEN cDNA 2310065D10	2144.988	2145.037	-0.049	377	395	2	NGQLYCTRENAPIRGPDGK
Spink5 protein/RIKEN cDNA 2310065D10	2153.053	2152.943	0.11	758	774	3	NTETNKSDKEDKCHEYR
Spink5 protein/RIKEN cDNA 2310065D10	2158.972	2159.056	-0.084	775	792	2	SMQLDGRLICTRENDPVR
Spink5 protein/RIKEN cDNA 2310065D10	2162.028	2162.053	-0.025	308	326	2	NGTLFCTRENDPIRGLDGK
Spink5 protein/RIKEN cDNA 2310065D10	2169.051	2168.992	0.059	667	683	2	CVMCKELLQKEMEETNK
Spink5 protein/RIKEN cDNA 2310065D10	2232.998	2233.031	-0.033	260	277	2	***CALCAEIFMRQFTEEKGK***
Spink5 protein/RIKEN cDNA 2310065D10	2245.005	2245.039	-0.034	436	455	2	NGMLMCTRENDPVVGPDGKR
Spink5 protein/RIKEN cDNA 2310065D10	2249.001	2248.92	0.081	925	944	2	STKEGDPEFSSSSRDSDMCK
Spink5 protein/RIKEN cDNA 2310065D10	2268.975	2268.984	-0.009	764	781	3	***SDKEDKCHEYRSMQLDGR***
Spink5 protein/RIKEN cDNA 2310065D10	2288.106	2288.027	0.079	724	743	3	GPDGAMHGNKCAMCKERLEK
Spink5 protein/RIKEN cDNA 2310065D10	2487.028	2487.098	-0.071	525	543	2	MYKNACFMCWAFFQQEAKK
Spink5 protein/RIKEN cDNA 2310065D10	2561.25	2561.206	0.044	260	280	3	***CALCAEIFMRQFTEEKGKAEK***
Spink5 protein/RIKEN cDNA 2310065D10	2569.198	2569.153	0.045	25	46	2	***GNQDPCMKFQAQMKNGTLTCPK***
Spink5 protein/RIKEN cDNA 2310065D10	2865.411	2865.329	0.082	528	551	2	NACFMCWAFFQQEAKKSGAGFRPK
Stablin1 protein	1333.651	1333.702	-0.051	496	507	0	WQLPPPLPGDSK
Stablin1 protein	1520.765	1520.819	-0.054	509	522	0	TVGQILASTEVFTR
Stablin1 protein	1678.789	1678.871	-0.082	446	459	0	WWTLAGQEVTITFK
Stablin1 protein	1972.97	1973.024	-0.054	596	613	0	VLTMANQVLTVNISEEGR
Stablin1 protein	1988.994	1989.019	-0.025	596	613	0	***VLTMANQVLTVNISEEGR***
Stathmin	972.506	972.498	0.008	54	61	1	LEAAEERR
Stathmin	1387.779	1387.745	0.034	15	27	0	ASGQAFELILSPR
Stathmin	1526.872	1526.797	0.075	28	41	1	SKESVPDFPLSPPK
Stathmin	1543.885	1543.846	0.039	14	27	1	RASGQAFELILSPR

Protein Name	Measured Mass (Da)	Calculated Mass (Da)	Error	Peptide Start	Peptide End	Missed cleavage	Sequence
Stathmin	1567.942	1567.86	0.082	30	43	2	ESVPDFPLSPPKKK
Stathmin	1602.914	1602.872	0.042	15	29	1	ASGQAFELILSPRSK
Stathmin	2000.055	2000.042	0.013	44	60	2	DLSLEEIQKKLEAAEER
Stromal antigen 2	925.525	925.454	0.071	907	914	0	DGIEFAFK
Stromal antigen 2	2143.995	2143.935	0.06	977	997	0	***NSLLAGGDDDTMSVISGMSSR***
Stromal antigen 2	2224.105	2224.076	0.029	698	715	0	VFCQICQHYLTNVNTTVK
Stromal antigen 2	2343.057	2343.175	-0.118	947	964	2	***RTVYVYLEKFMTFQMSLR***
Stromal antigen 2	2343.057	2343.175	-0.118	948	965	2	***TVYVYLEKFMTFQMSLRR***
Stromal antigen 2	2484.137	2484.185	-0.048	566	585	1	TYHALCNEEFTIFNRVDISR
Stromal antigen 2	2511.135	2511.172	-0.037	202	221	2	***RKELQENQDEIENMMNAIFK***
Stromal antigen 2	2644.211	2644.206	0.005	977	1002	1	***NSLLAGGDDDTMSVISGMSSRGSTVR***
Stromal antigen 2	2664.234	2664.203	0.03	1113	1135	1	IEDLNEGMDFDTMDIDLPPSKNR
Stromal antigen 2	2722.246	2722.209	0.037	425	447	0	***DWECMNSLLLEEPLSGEEALTDR***
Stromal antigen 2	2785.264	2785.373	-0.109	849	871	0	TLILSLQQLFNEMIQENGYNFDR
Stromal antigen 2	2828.265	2828.385	-0.121	1038	1061	1	***EQTLHTPVMMQTPQLTSTIMREPK***
Stromal antigen 2	2843.362	2843.338	0.024	977	1004	2	***NSLLAGGDDDTMSVISGMSSRGSTVRSK***
Stromal antigen 2	2890.34	2890.373	-0.033	204	227	1	ELQENQDEIENMMNAIFKGVFVHR
Stromal antigen 2	2975.515	2975.392	0.123	1137	1162	1	***ERTELKPDFFDPASIMDESVLGVSMF***
Synaptonemal complex protein 1	974.46	974.503	-0.043	571	578	1	DELESVRK
Synaptonemal complex protein 1	984.503	984.607	-0.105	136	143	2	KIIEAQRK
Synaptonemal complex protein 1	1087.561	1087.587	-0.026	300	308	1	TKLQDENLK
Synaptonemal complex protein 1	1157.571	1157.615	-0.044	128	136	2	ENKLQENRK
Synaptonemal complex protein 1	1228.74	1228.702	0.038	443	452	2	LLDEKKQVEK
Synaptonemal complex protein 1	1272.548	1272.692	-0.144	783	792	2	KQLEIEKEEK
Synaptonemal complex protein 1	1459.715	1459.803	-0.088	366	378	1	AKTTHSFVVTELK
Synaptonemal complex protein 1	1506.801	1506.774	0.026	948	961	0	QTPLSLSTPASFMK
Synaptonemal complex protein 1	1524.742	1524.778	-0.036	474	486	0	EVHDLQEQVTVTK
Synaptonemal complex protein 1	1530.708	1530.824	-0.116	118	130	2	VSIESELKQKENK
Synaptonemal complex protein 1	1549.688	1549.798	-0.11	742	754	1	IVEERDSELGLYK
Synaptonemal complex protein 1	1566.712	1566.829	-0.117	713	724	2	LQKEIDLRCQHK
Synaptonemal complex protein 1	1580.707	1580.805	-0.098	735	746	2	HKHQYDKIVEER
Synaptonemal complex protein 1	1592.712	1592.84	-0.128	647	660	1	SSAEIKQLNAYEIK
Synaptonemal complex protein 1	2012.923	2013.026	-0.103	309	325	2	ELSEKKDHLTSELEDIK
syntaxin binding protein 5-like	1334.766	1334.792	-0.026	488	498	3	LKTSKVFEKQK
syntaxin binding protein 5-like	1480.769	1480.795	-0.026	670	682	0	TVLLSMGTIDLYR
syntaxin binding protein 5-like	1510.756	1510.798	-0.042	545	557	0	HEVTTEIVSLEVR
syntaxin binding protein 5-like	1541.771	1541.791	-0.02	333	345	1	***ACRRPSLTIMHGK***
syntaxin binding protein 5-like	1562.765	1562.754	0.011	880	892	0	TGSLMQPPYEVWR
syntaxin binding protein 5-like	1588.759	1588.806	-0.047	683	695	2	SSDLYQRQPRSPR
syntaxin binding protein 5-like	1791.845	1791.886	-0.041	805	819	1	***EAITALYFMESFARK***
testicular protein	974.497	974.503	-0.006	607	614	1	DELESVRK
testicular protein	985.499	985.58	-0.081	732	740	1	LLGEVEKAK
testicular protein	1197.614	1197.592	0.022	752	760	1	EIDLRCQHK
testicular protein	1219.576	1219.667	-0.091	926	936	2	YIPTGGSNKKR
testicular protein	1289.673	1289.628	0.045	393	403	1	EAQMEELNKAK
testicular protein	1344.707	1344.724	-0.017	700	710	1	LELELESTKQR
testicular protein	1426.745	1426.73	0.015	350	361	1	KDHLTSELEDIK
testicular protein	1583.801	1583.918	-0.117	9	21	1	LTVPFHVVFIKER
testicular protein	1656.824	1656.825	-0.001	602	614	2	***EMHLRDELESVRK***
testicular protein	1718.848	1718.814	0.034	757	770	1	***CQHKIAEMVALMEK***
testicular protein	1781.922	1781.915	0.007	508	522	1	EKEVHDLQEQVTVTK

Protein Name	Measured Mass (Da)	Calculated Mass (Da)	Error	Peptide Start	Peptide End	Missed cleavage	Sequence
testicular protein	1789.896	1789.789	0.107	22	39	1	***TRATGAEPDNGPGGSMEK***
testicular protein	1798.938	1799.003	-0.065	732	748	2	LLGEVEKAKATVDEAVK
testicular protein	1835.92	1835.908	0.012	615	629	2	EFIQQGDEVKCKLDK
testicular protein	1851.877	1851.939	-0.062	312	326	2	ENKMKDLTFLLEESR
testicular protein	1994.973	1994.964	0.009	546	562	1	***NTELTASCDMLLLENKK***
testicular protein	2012.986	2013.026	-0.04	345	361	2	ELSEKKDHLTSELEDIK
testicular protein	2021.989	2022.092	-0.103	591	606	2	LLKQIENLEEKEMHLR
testicular protein	2055.03	2055.07	-0.04	984	1002	1	***QTPLSLSTPASFMKFGSLK***
testicular protein	2126.063	2126.026	0.036	868	887	2	***TTPSQNISRLSSSMDSGKSK***
testicular protein	2255.148	2255.163	-0.015	460	478	2	NNKEVELEELKNILAEDQK
Thrombospondin type-1 domain-containing protein 7A precursor	974.423	974.437	-0.014	414	421	0	ECPALEEK
Thrombospondin type-1 domain-containing protein 7A precursor	1014.546	1014.466	0.08	221	229	0	TCGSGLQHR
Thrombospondin type-1 domain-containing protein 7A precursor	1134.598	1134.472	0.126	1128	1137	0	DNCGEGVQTR
Thrombospondin type-1 domain-containing protein 7A precursor	1431.803	1431.688	0.115	461	474	0	ANQTALCGGGVQTR
Thrombospondin type-1 domain-containing protein 7A precursor	1441.651	1441.698	-0.047	328	338	0	YWDIQIGYQTR
Thrombospondin type-1 domain-containing protein 7A precursor	1522.704	1522.595	0.109	81	94	0	CMGDDCGPGGIQTR
Thrombospondin type-1 domain-containing protein 7A precursor	1769.861	1769.847	0.014	630	645	0	DAIFPIPVACDAPCPK
Thrombospondin type-1 domain-containing protein 7A precursor	2239.159	2239.101	0.058	764	781	0	CPESLRPETVRPCLLPCR
Tmcc1 protein	1068.581	1068.59	-0.009	113	121	2	RGTSLHSRR
Tmcc1 protein	1097.67	1097.605	0.065	114	123	2	GTSLHSRRGK
Tmcc1 protein	1128.701	1128.65	0.051	324	333	0	SAQTILQLQK
Tmcc1 protein	1171.671	1171.624	0.047	111	120	2	MKRGTSLHSR
Tmcc1 protein	1176.662	1176.734	-0.072	101	110	1	VLQQIRVPPK
Tmcc1 protein	1176.662	1176.598	0.064	23	32	1	QTESEQKLSK
Tmcc1 protein	1253.752	1253.706	0.046	113	123	3	RGTSLHSRRGK
Tmcc1 protein	1592.911	1592.893	0.018	107	120	3	VPPKMKRGTSLHSR
Topoisomerase (DNA) II beta	944.5	944.517	-0.017	288	296	0	LDETGVALK
Topoisomerase (DNA) II beta	974.443	974.43	0.013	450	459	0	LDDANDAGGK
Topoisomerase (DNA) II beta	1002.396	1002.429	-0.033	1406	1414	0	DEYAFSSGK
Topoisomerase (DNA) II beta	1013.553	1013.586	-0.034	1147	1154	1	VEELIKQR
Topoisomerase (DNA) II beta	1114.608	1114.616	-0.008	1081	1089	0	DLIQMLVQR
Topoisomerase (DNA) II beta	1741.846	1741.866	-0.02	981	996	1	***MTEEKLAQAEAAGLHK***
Topoisomerase (DNA) II beta	1746.883	1746.791	0.092	545	559	0	***IMIMTDQDQDGSHIK***
Topoisomerase (DNA) II beta	1900.984	1901.067	-0.083	560	575	0	GLLINFIHHNWPSLLK
Topoisomerase (DNA) II beta	1910.036	1910	0.036	316	333	1	GFQQISFVNSIATTKGGR
Topoisomerase (DNA) II beta	1910.036	1909.953	0.083	887	902	1	RMLEGLDPHPMLPNYK
Topoisomerase (DNA) II beta	1914.97	1914.972	-0.002	1021	1035	1	YETVQDILKEFFDLR
Topoisomerase (DNA) II beta	2332.049	2332.106	-0.057	1384	1405	1	VKASPITNDGEDEFVPSDGLDK
Topoisomerase (DNA) II beta	2758.3	2758.33	-0.03	997	1019	1	***VFKLQTTLTCNSMVLFDHMGCLK***
TPA: testase-7	933.494	933.445	0.049	491	498	1	ADGIRCSR
TPA: testase-7	1098.682	1098.621	0.061	1	10	1	***MTGAKVLVHK***
TPA: testase-7	1110.733	1110.658	0.075	71	79	1	***HIITMKPKK***
TPA: testase-7	1399.843	1399.812	0.031	6	16	2	***VLVHKRNMFLK***
TPA: testase-7	1705.757	1705.685	0.072	524	536	1	***SADEICYMEMNRR***
TPA: testase-7	1837.83	1837.892	-0.062	454	468	2	NCQFLKAGTVCRQEK
TPA: testase-7	1857.877	1857.781	0.096	499	512	2	GGYCYKMECQRHNR
TPA: testase-7	1916.893	1916.828	0.064	522	536	2	SRSADEICYMEMNRR
TPA: testase-7	1916.893	1916.828	0.064	521	535	2	RSRSADEICYMEMNR
Tpr	974.415	974.503	-0.088	738	745	0	EITSLQER
Tpr	1002.412	1002.52	-0.108	60	67	1	RLSQSQER
Tpr	1002.412	1002.498	-0.086	118	125	1	EELEAEKR

Protein Name	Measured Mass (Da)	Calculated Mass (Da)	Error	Peptide Start	Peptide End	Missed cleavage	Sequence
Tpr	1114.568	1114.609	-0.041	835	843	1	QRLNSQIEK
Tpr	1403.849	1403.761	0.088	148	159	2	LNEKLKESNTTK
Tpr	1445.883	1445.79	0.093	450	463	1	AVASLSAKLEQAMK
Tpr	1522.894	1522.871	0.023	253	265	2	HVEDLLTKLKEAK
Tpr	1647.876	1647.813	0.063	261	274	2	LKEAKEQQASMEEK
Tpr	1724.918	1724.876	0.042	1085	1100	0	***ELMLHAADVEALQAAK***
Tpr	1815.063	1814.955	0.108	230	244	2	KEEVLRLEEQMNGLK
Tpr	1834.837	1834.851	-0.014	1120	1134	1	AESQLLECKASWEER
Tpr	1836.903	1836.968	-0.066	979	993	2	EKQVTEEVHKNIEVR
Tpr	1943.065	1943.031	0.033	349	365	2	LEKELENANDLLSATKR
Tpr	2272.146	2272.147	-0.001	1101	1119	2	EQVSKMTSIRQHLEETTQK
Tpr	2288.149	2288.142	0.007	1101	1119	2	***EQVSKMTSIRQHLEETTQK***
Tpr	2328.175	2328.198	-0.023	1170	1191	1	VVTSMKDAVQAPLNVSLNEEGK
Tpr	946.49	946.508	-0.018	20	27	1	STQNKLEK
Tpr	946.49	946.515	-0.025	341	348	1	EMLEKIGK
Tpr	960.475	960.451	0.024	98	105	0	ELETAQDR
Tpr	1287.61	1287.641	-0.031	1014	1023	2	EKQELQDDKR
Tpr	1326.645	1326.75	-0.105	80	90	1	LELEKLNNQVK
Tpr	1332.693	1332.731	-0.038	341	351	2	***EMLEKIGKLEK***
Tpr	1335.653	1335.652	0	440	449	2	QREEYERAQK
Tpr	1445.838	1445.79	0.048	450	463	1	AVASLSAKLEQAMK
Tpr	1537.718	1537.809	-0.091	879	891	0	QLDTEINLHLNTK
Tpr	1556.758	1556.826	-0.068	798	810	1	LSQQRESLLAEQR
Tpr	1593.776	1593.773	0.003	336	348	2	***DQMEKEMLEKIGK***
Tpr	1609.798	1609.768	0.03	336	348	2	***DQMEKEMLEKIGK***
Tpr	1632.75	1632.838	-0.088	1025	1038	1	AIESMEQQLSELKK
Tpr	1632.75	1632.838	-0.088	1024	1037	1	KAIESMEQQLSELK
Tpr	1682.794	1682.847	-0.053	1176	1191	0	DAVQAPLNVSLNEEGK
Tpr	1686.788	1686.86	-0.072	231	244	1	EEVLRLEEQMNGLK
Tpr	1686.788	1686.853	-0.065	464	477	2	EIQRLQEDTDKANK
Tpr	1698.803	1698.889	-0.086	943	956	2	QAEEQVNDLKERLK
Tpr	1701.799	1701.911	-0.113	60	73	2	RLSQSQERLVTETR
Tpr	1715.804	1715.934	-0.13	789	802	2	EMLKLSEVRLSQQR
Tpr	1730.83	1730.825	0.005	1067	1081	1	DCQEQAKIAVEAQNK
Tpr	1802.804	1802.872	-0.068	46	59	1	FKVESEQQYFEIEK
Tpr	1829.865	1829.936	-0.071	685	699	2	EKIDSEKLQNEQLEK
transaldolase 1	980.512	980.511	0.002	231	239	0	TIVMGASFR
transaldolase 1	1132.625	1132.624	0.001	315	324	1	FAADAIKLER
transaldolase 1	1228.71	1228.702	0.008	259	269	1	LLGELLKDNSK
transaldolase 1	1251.64	1251.628	0.012	111	121	1	LSFDKDAMVAR
transaldolase 1	1267.641	1267.623	0.018	111	121	1	***LSFDKDAMVAR***
transaldolase 1	1317.683	1317.67	0.012	9	19	1	QRMESALDQLK
transaldolase 1	1333.679	1333.665	0.014	9	19	1	***QRMESALDQLK***
transaldolase 1	1391.726	1391.711	0.015	246	258	0	ALAGCDFLTISPK
transaldolase 1	1438.757	1438.756	0	193	204	1	ILDWHVANTDKK
transaldolase 1	1498.706	1498.687	0.019	296	307	0	WLHNEDQMAVEK
transaldolase 1	1514.698	1514.682	0.017	296	307	0	***WLHNEDQMAVEK***
transaldolase 1	1670.767	1670.81	-0.043	278	292	1	AAQTSDSEKIHLDEK
triosephosphate isomerase	953.476	953.476	0	7	14	0	FFVGGNWK
triosephosphate isomerase	1457.691	1457.714	-0.023	101	113	0	HVFGESDELIGQK
triosephosphate isomerase	1539.75	1539.776	-0.026	6	18	2	KFFVGGNWKMNGR

Protein Name	Measured Mass (Da)	Calculated Mass (Da)	Error	Peptide Start	Peptide End	Missed cleavage	Sequence
triosephosphate isomerase	1539.75	1539.776	-0.026	7	19	2	FFVGGNWKMNGRK
triosephosphate isomerase	1662.758	1662.828	-0.07	70	85	0	***VTNGPFTGEISPGMIK***
triosephosphate isomerase	1683.913	1683.866	0.047	6	19	3	***KFFVGGNWKMNGRK***
triosephosphate isomerase	1683.913	1683.866	0.047	7	20	3	***FFVGGNWKMNGRKK***
triosephosphate isomerase	1730.86	1730.869	-0.009	191	206	1	GWLKSNVNDGVAQSTR
triosephosphate isomerase	1734.878	1734.9	-0.022	176	190	1	TATPQQAQEVHEKLR
triosephosphate isomerase	2096.039	2096.055	-0.016	1	18	3	MAPTRKFFVGGNWKMNGR
TUFM protein, Tu translation elongation factor, mitochondrial	1184.625	1184.614	0.011	316	327	0	AEAGDNLGALVR
TUFM protein, Tu translation elongation factor, mitochondrial	1276.674	1276.648	0.026	301	311	0	***TVVTGIEMFHK***
TUFM protein, Tu translation elongation factor, mitochondrial	1571.891	1571.855	0.036	239	252	0	LLDAVDTYIPVPTR
TUFM protein, Tu translation elongation factor, mitochondrial	1669.879	1669.874	0.005	312	327	1	SLERAEAGDNLGALVR
TUFM protein, Tu translation elongation factor, mitochondrial	1672.851	1672.853	-0.002	105	120	0	GITINAAHVEYSTAAR
TUFM protein, Tu translation elongation factor, mitochondrial	1766.837	1766.847	-0.01	89	102	2	FKKYEEIDNAPEER
TUFM protein, Tu translation elongation factor, mitochondrial	1781.868	1781.883	-0.015	286	300	2	KGDECELLGHNKNIR
TUFM protein, Tu translation elongation factor, mitochondrial	1808.924	1808.927	-0.004	54	70	0	DKPHVNVGTIGHVDHGK
ubiquitin fusion degradation 1 like	1122.601	1122.582	0.019	256	264	1	RGIPNYEFK
ubiquitin fusion degradation 1 like	1310.701	1310.661	0.04	290	301	0	FIAFSGEGQSLR
ubiquitin fusion degradation 1 like	1335.719	1335.689	0.03	227	239	2	AFSGSGNRLDGKK
ubiquitin fusion degradation 1 like	1438.816	1438.756	0.06	290	302	1	FIAFSGEGQSLRK
ubiquitin fusion degradation 1 like	1455.819	1455.775	0.044	46	58	0	***IIMPPSALDQLSR***
ubiquitin fusion degradation 1 like	1647.834	1647.919	-0.085	240	255	1	KGVEPSPSPIKPGDIK
ubiquitin fusion degradation 1 like	1675.947	1675.925	0.022	241	256	1	GVEPSPSPIKPGDIKR
ubiquitin fusion degradation 1 like	1697.901	1697.912	-0.011	43	58	1	***GGKIIMPPSALDQLSR***
ubiquitin fusion degradation 1 like	1803.98	1804.02	-0.04	240	256	2	KGVEPSPSPIKPGDIKR
ubiquitin-conjugating enzyme E2N	969.521	969.539	-0.018	95	102	0	WSPALQIR
ubiquitin-conjugating enzyme E2N	1035.601	1035.632	-0.031	15	24	0	LLAEPVPGIK
ubiquitin-conjugating enzyme E2N	1351.648	1351.67	-0.022	142	152	1	AWTRLYAMNNI
ubiquitin-conjugating enzyme E2N	1549.841	1549.882	-0.041	11	24	1	ETQRLLAEPVPGIK
ubiquitin-conjugating enzyme E2N	1746.888	1746.889	-0.001	54	68	0	LELFLPEEYPMAAPK
ubiquitin-conjugating enzyme E2N	2195.068	2195.068	0	34	53	0	YFHVVIAGPQDSPFEGGTFK
UMP/CMP kinase	963.521	963.517	0.003	89	96	0	FLIDGFPR
UMP/CMP kinase	1205.648	1205.655	-0.007	87	96	1	NKFLIDGFPR
UMP/CMP kinase	1478.771	1478.751	0.02	27	39	0	YGYTHLSAGELLR
UMP/CMP kinase	1848.936	1848.982	-0.046	174	190	2	KIDASKSVDEVFGEVVK
UMP/CMP kinase	1878.912	1878.922	-0.01	27	42	1	YGYTHLSAGELLRDER
UMP/CMP kinase	1895.944	1895.951	-0.007	180	196	2	SVDEVFGEVVKIFDKEG
UMP/CMP kinase	2348.155	2348.211	-0.056	23	42	2	IVEKYGYTHLSAGELLRDER
UMP/CMP kinase	2613.334	2613.36	-0.026	152	173	1	***IQTYLESTKPIIDLYEEMGKVK***
Ureb1 protein	1737.902	1737.933	-0.031	292	306	0	QLAAFLEGFYEIIPK
Ureb1 protein	1753.882	1753.92	-0.038	258	273	0	DLKPNGANILVTEENK
Ureb1 protein	1839.871	1839.866	0.005	8	24	0	EPSSMHISSSLPPDTQK
Ureb1 protein	1887.952	1887.921	0.031	428	444	0	HMLLLAIQECSEGFGLA
Ureb1 protein	1903.944	1903.916	0.028	428	444	0	***HMLLLAIQECSEGFGLA***
Ureb1 protein	1964.956	1964.983	-0.027	116	133	0	LYIVFEGEEGQDAGGLLR
Valosin-containing protein p97/p47 complex-interacting protein p135	1381.693	1381.661	0.031	412	423	0	LVAAMEEVFMDK
Valosin-containing protein p97/p47 complex-interacting protein p135	1548.838	1548.775	0.063	806	817	0	EFNIPPYLQCIR
Valosin-containing protein p97/p47 complex-interacting protein p135	1565.76	1565.742	0.018	1108	1121	0	LQEMVSSIQASMDK
Valosin-containing protein p97/p47 complex-interacting protein p135	1647.769	1647.859	-0.09	239	251	1	ENLKQHFQQHLAR
Valosin-containing protein p97/p47 complex-interacting protein p135	1658.777	1658.785	-0.008	1125	1139	1	DQSAEQAPSDLSQRK
Valosin-containing protein p97/p47 complex-interacting protein p135	1723.812	1723.812	0	550	565	1	GDGSIVYLDGDRTNSR
Valosin-containing protein p97/p47 complex-interacting protein p135	1851.878	1851.924	-0.046	313	330	0	SSGDYSATFLPGLIPAEK

Protein Name	Measured Mass (Da)	Calculated Mass (Da)	Error	Peptide Start	Peptide End	Missed cleavage	Sequence
Valosin-containing protein p97/p47 complex-interacting protein p135	1942.93	1942.989	-0.059	408	423	1	***YLLRLVAAMEEVFMDK***
Valosin-containing protein p97/p47 complex-interacting protein p135	1950.942	1950.921	0.021	818	834	1	***YGFPPKELMPPQAGMEK***
Valosin-containing protein p97/p47 complex-interacting protein p135	1971.968	1971.986	-0.018	1108	1124	1	LQEMVSSIQASMDKHLR
Valosin-containing protein p97/p47 complex-interacting protein p135	1981.968	1981.953	0.015	912	928	0	***GLPHMFQQGGVFYNIMK***
Valosin-containing protein p97/p47 complex-interacting protein p135	1997.939	1997.948	-0.009	912	928	0	***GLPHMFQQGGVFYNIMK***
Valosin-containing protein p97/p47 complex-interacting protein p135	2009.944	2009.955	-0.011	657	674	1	KNPDDYTPVNIDGAHAQR
vav 2 oncogene	1082.609	1082.608	0.001	115	124	0	LSLHSIAQSK
vav 2 oncogene	1125.651	1125.727	-0.076	331	339	1	VLKYHLLLK
vav 2 oncogene	1142.647	1142.608	0.039	434	442	2	RKGYSYELK
vav 2 oncogene	1142.647	1142.529	0.118	815	823	1	YNFAARDMR
vav 2 oncogene	1587.735	1587.738	-0.003	452	464	2	***MTDDPMHNKDIKK***
vav 2 oncogene	1659.724	1659.774	-0.051	839	853	1	IGGDQGWWKGETNGR
vav 2 oncogene	1677.75	1677.839	-0.089	211	224	1	TLEDIEKNYMGPLR
vav 2 oncogene	1680.748	1680.727	0.021	478	490	1	***QGFQFFCKTEDMK***
vav 2 oncogene	1707.76	1707.86	-0.1	306	319	2	VEECTLRVQDGKFK
vav 2 oncogene	1735.728	1735.822	-0.094	194	207	1	RSCCLLEIQETEAK
vav 2 oncogene	1740.776	1740.872	-0.096	815	828	2	YNFAARDMRELSLR
vav 2 oncogene	1836.794	1836.828	-0.034	478	491	2	***QGFQFFCKTEDMKR***
vimentin	1114.607	1114.561	0.046	85	93	0	VELQELNDR
vimentin	1443.74	1443.699	0.041	31	44	0	SLYSSSPGGAYVTR
vimentin	1494.795	1494.778	0.017	17	30	0	TYSLGSALRPSTSR
vimentin	1775.854	1775.854	0	275	290	1	FADLSEAANRNNDALR
vimentin	1823.898	1823.883	0.015	126	139	2	***LGDLYEEEMRELRR***
vimentin	1837.886	1837.952	-0.067	405	420	1	ETNLESLPLVDTHSKR
vimentin	1990.974	1990.981	-0.007	273	290	2	SKFADLSEAANRNNDALR
vimentin	2313.079	2313.09	-0.011	254	272	1	QQYESVAAKNLQEAEEWYK
vimentin	2376.111	2376.158	-0.047	302	322	1	QVQSLTCEVDALKGTNESLER
vimentin	2496.213	2496.249	-0.036	59	80	1	LLQDSVDFSLADAINTEFKNTR
Wbscr1 alternative spliced product	943.48	943.465	0.015	155	163	1	***RAGPPMGSR***
Wbscr1 alternative spliced product	956.541	956.519	0.022	164	171	1	FRDGPPLR
Wbscr1 alternative spliced product	956.541	956.49	0.051	147	155	1	GGSRPGDRR
Wbscr1 alternative spliced product	1114.619	1114.609	0.01	110	119	1	SLRVDIAEGR
Wbscr1 alternative spliced product	1119.543	1119.542	0.001	130	139	2	KGGPDDRGYR
Wbscr1 alternative spliced product	1138.584	1138.588	-0.004	120	129	2	KQDKGGFGFR
Wbscr1 alternative spliced product	1138.584	1138.588	-0.004	121	130	2	QDKGGFGFRK
Wbscr1 alternative spliced product	1200.567	1200.563	0.004	11	22	1	AYSSFGGGRGSR
Wbscr1 alternative spliced product	1242.639	1242.704	-0.065	110	120	2	SLRVDIAEGRK
Wbscr1 alternative spliced product	1392.679	1392.688	-0.009	97	109	0	EALTYDGALLGDR
Wbscr1 alternative spliced product	1779.836	1779.847	-0.011	164	178	2	***FRDGPPLRGSNMDFR***
Wbscr1 alternative spliced product	1981.856	1981.912	-0.057	81	96	1	FKGFCYVEFDEVDSLK
zinc finger and BTB domain containing 41 homolog	953.52	953.54	-0.02	158	165	1	SNLTVHRK
zinc finger and BTB domain containing 41 homolog	1142.66	1142.63	0.03	371	379	2	HDHLTKHKK
zinc finger and BTB domain containing 41 homolog	1432.785	1432.778	0.007	154	165	2	YSTKSNLTVHRK
zinc finger and BTB domain containing 41 homolog	1668.767	1668.795	-0.029	214	226	3	TKSESWKCDICKK
zinc finger and BTB domain containing 41 homolog	1668.767	1668.795	-0.029	213	225	3	KTKSESWKCDICK
zinc finger and BTB domain containing 41 homolog	1768.787	1768.761	0.026	380	394	1	IHSGEKAHQCEECGK
zinc finger and BTB domain containing 41 homolog	1770.785	1770.828	-0.043	177	190	2	EHKCPYCNKLHASK
zinc finger and BTB domain containing 41 homolog	1946.761	1946.799	-0.038	352	366	2	VHHDDKRYECDECGK
Zinc finger DHHC domain-containing protein 13	1104.653	1104.581	0.072	205	214	0	FNPSLSVVDK
Zinc finger DHHC domain-containing protein 13	1110.674	1110.596	0.078	262	270	0	NQLISHMLR
Zinc finger DHHC domain-containing protein 13	1122.638	1122.534	0.104	394	403	0	TWATDPGFTK

Protein Name	Measured Mass (Da)	Calculated Mass (Da)	Error	Peptide Start	Peptide End	Missed cleavage	Sequence
Zinc finger DHHC domain-containing protein 13	1172.759	1172.64	0.119	236	246	0	LLEAGSSLDIR
Zinc finger DHHC domain-containing protein 13	1269.795	1269.732	0.063	193	204	0	VIGPEPTGFLLK
Zinc finger DHHC domain-containing protein 13	1304.774	1304.627	0.147	250	261	0	***GETPLDMALQSK***
Zinc finger MYM-type protein 3	1066.558	1066.592	-0.034	731	738	0	LLETIHWR
Zinc finger MYM-type protein 3	1079.537	1079.51	0.027	1342	1350	0	SMLESMLNR
Zinc finger MYM-type protein 3	1521.756	1521.75	0.006	289	301	2	***SPRMSLRSSMAQR***
Zinc finger MYM-type protein 3	1656.759	1656.756	0.003	1074	1087	1	CWVQSKYANGETSK
Zinc finger MYM-type protein 3	1811.835	1811.764	0.071	302	317	2	***AGRSSMGTKMSCAHCR***
Zinc finger MYM-type protein 3	1837.856	1837.87	-0.014	1299	1312	1	FYEFYLSKCPESLR
Zinc finger MYM-type protein 3	1987.927	1987.919	0.008	986	1003	0	MANVLDEPGQDLEADFPK
Zinc finger MYM-type protein 3	1992.934	1992.96	-0.026	789	806	1	VENNHTVRTPDENGNLGK
Zinc finger MYM-type protein 3	2022.945	2022.953	-0.009	1325	1341	0	SCIAESPLWYSVIPMDR
Zinc finger MYM-type protein 3	2032.972	2032.913	0.059	564	579	1	VDRTVYQFCSPSCWTK
Zinc finger MYM-type protein 3	2085.992	2086.067	-0.075	536	553	2	VKQAGLTGPPRPCSFCRR
Zinc finger protein 100	985.518	985.545	-0.027	398	405	1	AHAQRLYK
Zinc finger protein 100	1655.743	1655.831	-0.088	761	773	2	TFHQRSCLSKHQK
Zinc finger protein 100	1707.746	1707.77	-0.024	830	844	0	IHTGEKPYTCGTCGK
Zinc finger protein 100	1740.802	1740.861	-0.059	562	575	1	HNKIHTIEGLYECK
Zinc finger protein 100	1774.781	1774.871	-0.09	367	380	3	HQKTPTNAKCFRCK
Zinc finger protein 100	1787.783	1787.848	-0.065	543	556	2	HFGCAKCKETFIYK
Zinc finger protein 100	1793.746	1793.739	0.007	424	438	1	KSHFPSAACECQGCR
Zinc finger protein 100	1793.746	1793.739	0.007	425	439	1	SHFPSAACECQGCRK
Zinc finger protein 100	1804.784	1804.822	-0.038	802	816	0	IHTGEKPYVCQECGK
Zinc finger protein 100	1839.828	1839.875	-0.047	379	394	2	CKKCGETFSGAFHLAK
Zinc finger protein 100	1844.776	1844.873	-0.097	382	397	1	CGETFSGAFHLAKHQR
Zinc finger protein 100	1972.869	1972.968	-0.099	381	397	2	KCGETFSGAFHLAKHQR
zinc finger protein 445	977.478	977.5	-0.022	675	682	0	SSYIIHMK
zinc finger protein 445	977.478	977.54	-0.062	515	522	1	LHQKTHSK
zinc finger protein 445	985.482	985.436	0.046	410	417	0	SSHHYNNK
zinc finger protein 445	990.467	990.539	-0.072	389	396	2	CVSVSRKR
zinc finger protein 445	1002.456	1002.488	-0.032	566	573	0	SYAIEHQR
zinc finger protein 445	1215.589	1215.573	0.016	935	944	2	CSTCGKTFKK
zinc finger protein 445	1283.595	1283.627	-0.032	451	460	1	KSWHAHPEHR
zinc finger protein 445	1437.696	1437.772	-0.076	602	612	2	LHHKEVYKQEK
zinc finger protein 445	1474.73	1474.704	0.025	60	72	0	YHESSGPLETLSR
zinc finger protein 445	1498.735	1498.764	-0.029	857	869	1	DFVGIHARSVDQR
zinc finger protein 445	1525.735	1525.664	0.071	743	753	1	EKPYQCRECEK
zinc finger protein 445	1530.734	1530.744	-0.01	406	417	2	NFRKSSHHYNNK
zinc finger protein 445	1583.74	1583.7	0.04	931	943	2	ACHKCSTCGKTFK
zinc finger protein 445	1599.77	1599.698	0.072	461	473	0	QPSYSEEGLFQCR
zinc finger protein 445	1638.793	1638.874	-0.081	726	738	1	AFHNRSFLLIHER
zinc finger protein 445	1638.793	1638.757	0.036	561	573	1	NFSCKSYAIEHQR
zinc finger protein 445	1703.82	1703.86	-0.04	832	844	2	SFDRNYRLVNHQR
zinc finger protein 445	1837.85	1837.873	-0.023	532	547	0	NALTCSLDVSHHLTDR
zinc finger protein 445	1843.822	1843.87	-0.048	633	647	2	TFPCQNCGKTFTQKK
zinc finger protein 445	1976.921	1976.935	-0.014	445	460	2	YGETSRKSWHAHPEHR
Zmym2 protein	984.528	984.549	-0.021	541	549	0	IPATVEDLK
Zmym2 protein	1098.632	1098.582	0.05	115	124	0	QVGSHPSFLK
Zmym2 protein	1152.691	1152.629	0.062	321	329	1	LLYKQDFAR
Zmym2 protein	1199.698	1199.675	0.022	541	551	1	IPATVEDLKSK
Zmym2 protein	1541.784	1541.818	-0.034	698	710	0	QLDEDLLVLDELK

Protein Name	Measured Mass (Da)	Calculated Mass (Da)	Error	Peptide Start	Peptide End	Missed cleavage	Sequence
Zmym2 protein	1656.783	1656.811	-0.028	312	324	1	RPFCSEGCKLLYK
Zmym2 protein	1768.81	1768.848	-0.038	441	457	1	MTGSAPPPSPTPNKEMK
Zmym2 protein	1787.848	1787.76	0.088	410	423	0	FYCQQNEPNMTTQK
Zmym2 protein	1818.838	1818.77	0.068	53	66	1	NMTHKLCSDHCFNR
Zmym2 protein	1968.829	1968.87	-0.041	128	143	1	***DHMQDSFLMQPEKYGK***
Zmym2 protein	1984.836	1984.865	-0.029	128	143	1	***DHMQDSFLMQPEKYGK***
Zmym2 protein	1989.869	1989.895	-0.026	424	440	1	GPENLHYDQGCQTSRTK
